# Metal consumption of a middle-range society in the late 3^rd^ millennium BC Anatolia: A new socioeconomic approach

**DOI:** 10.1371/journal.pone.0269189

**Published:** 2022-06-03

**Authors:** Gonca Dardeniz, Tayfun Yıldırım

**Affiliations:** 1 Department of Protohistory and Near Eastern Archaeology, Faculty of Letters, Istanbul University, Istanbul, Turkey; 2 Department of Protohistory and Near Eastern Archaeology, Faculty of Language History and Geography, Ankara University, Ankara, Turkey; University at Buffalo - The State University of New York, UNITED STATES

## Abstract

This article discusses the socioeconomic dynamics of metal consumption patterns in the 3^rd^ millennium BC north-central Anatolian site of Resuloğlu (Çorum, Turkey). The socio-political structure of the site confirms a nonstate, socially complex community with a range of hierarchical and heterarchical expressions. This study presents the results of archaeological, compositional (n = 307), and isotopic (n = 45) analyses of the complete metal collection of Resuloğlu uncovered through two decades of systematic excavations with a well-established chronology. The elemental compositions of metal objects obtained with pXRF combined with lead isotope analysis denote a high diversity in alloy types and sources. The compositional analysis highlights the consumption of various binary and ternary alloys for different object types. The lead isotope ratios confirm the use of both in proximity to metallic sources and access to macro-regional trade extending from the Black Sea coast towards the Taurus Mountain range. The site appears as a part of linkages whereby goods and valuables were exchanged within decentralized networks of middle-range societies. The diversity in metal consumption suggests group-driven choices and networks rather than top-down control of social elites. This allows us to confront the conventional approach to the role of metals as the primary motivator for social complexity and inequality in all parts of the 3^rd^ millennium BC Anatolia.

## Introduction

Archaeology of the 3^rd^ millennium BC (i.e., early bronze age) Anatolia suffers from over-ambitious readings of archaeological and analytical data to relate metal production and consumption to social inequality. The production and consumption of metals, specifically the alloy of copper and tin (hereafter bronze), are equated to hierarchical complex societies and political hierarchy. The publications have flooded with terms like elite, prestige, luxury, exotic, or strategic, even though there is little or no evidence to support such terminology. Wealth objects and prestige goods, which were defined as products only accessible by the elites via long-distance trade or special craft products produced under the control of elite patrons [1: p. 10], have been used in ways improper to their anthropological definitions. Valuable metal objects were equalized to prestige goods, without confirmation of the presence of elites.

White and Hamilton [2: p. 49–90] suggest that a centralized structure was not always necessary for production, and metals do not always lead to social inequality [2: p. 50–90]. This new model to interpret metallurgical practices in nonstate, decentralized societies offers a framework for the 3^rd^ millennium BC Anatolian metallurgy.

The use of bronze has still widely accepted as one of the primary indices of complexity and advancement of Anatolian bronze age societies [e.g., 3]. This study hypothesizes that metal production and consumption patterns in the 3^rd^ millennium BC Anatolian middle-range societies have not necessarily led to social inequality in every settlement. We argue that north-central Anatolia displays different lines of vertical and horizontal hierarchical relations and that diversity in socioeconomic systems has been overlooked.

Excavations at Resuloğlu (Çorum, Turkey), conducted between 2003–2019, uncovered both a settlement and a cemetery. The community of Resuloğlu represents primarily the end-users of metal products. The site provides us with the opportunity to discuss metal consumption and mobilization of commodities to obtain wealth and sustain control at the village level located in an environmentally diverse landscape. Resuloğlu offers a unique study area to reconsider the mainstream concept of the isolated and homogeneous character of Anatolian highland communities during the late 3^rd^ millennium BC as well as to examine the role of metals in a middle-range society.

The significance of this study relies on the systematic assessment of Resuloğlu’s complete metal corpus. There are two main objectives: 1) to lay out detailed archaeological and analytical evidence on the metals, and 2) to position the data in the broader socio-economic context of the diversified trans-egalitarian communities of the 3^rd^ millennium BC north-central Anatolia. This study drives data from multidisciplinary methods and interprets the information in the frame of diverse pathways of social complexity and inequality in middle-range societies. This approach presents important implications to understand the metal economics of nonstate societies during the late 3^rd^ millennium BC in north-central Anatolia.

## The archaeological context: Social inequality in the 3^rd^ millennium BC north-central Anatolian middle-range societies

The present state of research on the 3^rd^ millennium BC Anatolian archaeology has a series of major problems, among which is a lack of secure chronologies and a dearth of well-excavated and published sites [4]. North-central Anatolia is no exception. The first half of the 3^rd^ millennium BC has left a little archaeological trace in the region. The latter half of the millennium on the contrary is documented through a rich material culture composed mainly of ceramics and metals. The majority of the material corpus from the region has been recovered from grave contexts, whereas evidence from domestic contexts is thin. Most of the sites are dated approximately to 2500/2400–2100/2050 BC through relative chronologies. The regional chronology is heavily dependent on ceramics and metal typologies and has several handicaps, along with the challenges related to burial contexts.

**Fig 1:**
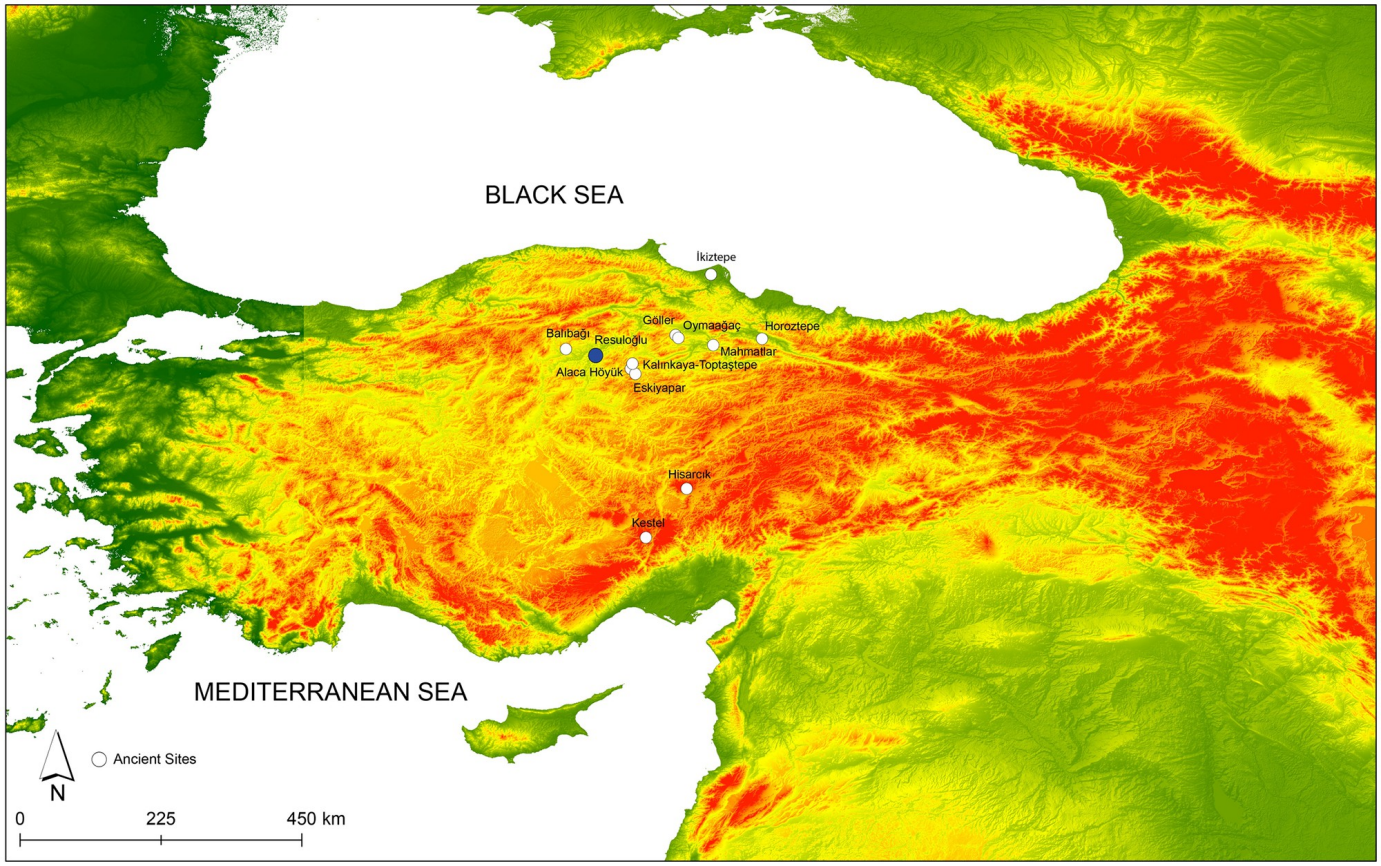
Map of the sites frequently referenced in the article. Resuloğlu is labeled in blue (map created by D. Yazıcı and Dardeniz).

The majority of the late 3^rd^ millennium BC archaeological sites located in north-central Anatolia are cemeteries such as Alaca Höyük (Çorum), Horoztepe (Tokat), Kalınkaya-Toptaştepe (Çorum), Balıbağı (Çankırı), Göller (Amasya), and Oymaağaç (Amasya) [5; 6 with references cited therein]. Yet the settlements associated with those cemeteries have mostly remained unidentified (Fig 1). While grave goods overarch the domestic material corpus, they could be representatives of some of the daily and utilitarian material corpora. A good example of how the material culture of a settlement has been represented in the cemetery area comes from the contemporaneous western Anatolian settlement of Demircihöyük with its coexisting cemetery of Sarıket. The Sarıket cemetery has pithoi from domestic contexts that have been used for burials, and utilitarian ceramics were found as grave goods [7].

In north-central Anatolia, associated settlements and cemetery areas are little known. Horoztepe, Balıbağı, and Kalınkaya-Toptaştepe [8] provided scattered architectural features of settlement areas; however, there is no clear evidence. Following this, more than half of the sites have been exposed during illegal activities causing a complete loss of contextual information. These major problems require further refining of the regional chronology, where possible, though any attribution to archaeological context should be done with caution.

The rich metal corpus of north-central Anatolia has attracted scholarly interest since the publication of metal artifacts from the 13 so-called ‘royal/princely graves’ of Alaca Höyük [9–12]. Mortuary traditions came into view also through funerary festivities such as specifically aligned cattle skulls and hooves on the shaft graves [e.g., 13]. Most of the discussion abounds about the symbolism and chemical composition of the extravagant metal objects such as sun-discs, zoomorphic and anthropomorphic figurines, jewelry, implements, and weapons made out of gold, silver, electrum, copper and its alloys, and even iron. Alas, only elaborate artifacts were given attention for archaeological and archaeometric analysis, preventing us from assessing a comprehensive understanding of corpora. Research focusing on the qualitative and quantitative characteristics of metal grave goods has overshadowed their social and behavioral context.

How were the north-central Anatolian societies organized during the late 3^rd^ millennium BC? Was there really a ‘royalty’ to have the Alaca Höyük graves called after them? Were these groups all small-scale tribal and local [14, 15], or were they all based on elite-controlled, long-distance trade [16]? Could there be another way to evidence variety in societal evolution, which allows both options to be true? For example, could the social complexity of the region contain egalitarian groups with a range of inequality and hierarchical societies with egalitarian elements coexisting among these nonstate societies [17–20]?

The existing material evidence confirms the prosperity of the late 3^rd^ millennium BC communities in north-central Anatolia. The richness of goods in graves or hoards at Alaca Höyük, Horoztepe, and Eskiyapar has been mostly explained as elite groups controlling production and trade. The archival or sacrificial values of such ostentatious metals and their legitimacy through consumption of unique items have been discussed as important elements of value systems [21–23]. Arguments relating metals to elites are mostly based on the presence of fully flourishing trading activity in the early 2^nd^ millennium BC and the existence of certain nonlocal artifacts in Anatolia such as Syrian bottles, ivory, or lapis lazuli objects [4]. However, the illiteracy of Anatolia during the 3^rd^ millennium BC prevents us from reconstructing the exact nature of this trade.

There is also a difference in societal types among the 3^rd^ millennium BC communities. Some sites were considered as more prosperous (e.g., Alaca Höyük, Eskiyapar, Mahmatlar, Horoztepe) than others (e.g., Balıbağı, Kalınkaya-Toptaştepe). Prosperity-related arguments were based on the relation between the production and consumption of bronze and centralized, hierarchical complex societies [e.g., 13, 24, 25]. However, not much has been hypothesized related to a political economy sponsoring this wealth and controlling the material flow. This gap in archaeological knowledge is especially true regarding small-scale, noncentralized village communities with some expressions of status inequality in the region, such as at Resuloğlu.

The concept of middle-range societies appears particularly useful to examine evolutionary societal types of the 3^rd^ millennium BC north-central Anatolian communities. Egalitarian, noncentralized groups, formerly described as tribes, show a range of hierarchy and inequality [18, 26]. Centralized groups and even states demonstrate an egalitarian, heterarchical pattern [18, 27]. Middle-range societies, however, embrace diverse societal types, ranging between mobile bands and bureaucratic states [28]. This concept explains the social inequality of societies formerly defined as tribes or chiefdoms by showing that complexity does not show a linear pattern but has diversity among nonstate societies [27].

In the north-central Anatolian 3^rd^ millennium BC, chiefdoms with leading elites controlling metal production and circulation have been accepted as the default societal type. Discussions abound around elites, prestige goods, control, and surplus. Yet not much has been explored regarding non-elite egalitarian groups that show elements of social inequality. In this study, we argue that there is variety in social inequality and hierarchical relations in the region during the second half of the 3^rd^ millennium BC by deriving data from analytical methods. Resuloğlu, formerly described as chiefdom [5, 29, 30], demonstrates certain egalitarian and heterarchical elements that lead us to describe it properly as a middle-range society.

## Resuloğlu: Settlement, cemetery, social structure

Resuloğlu is located in the Delice Valley of north-central Anatolia, at the northern edge of the modern city of Çorum and within the borders of Uğurludağ. The site is close to modern Çankırı and Ankara (Fig 1). It is a suite of sites consisting of settlements recorded as 1) northwest mound yielding late 4^th^ millennium BC and a few late 3^rd^ millennium BC sherds; 2) southeast mound yielding late 3^rd^ millennium BC deposits and a thin layer of 1^st^ millennium BC; 3) north mound yielding fragmentary 3^rd^ millennium BC evidence; 4) Resuloğlu II mound yielding fragmentary remains of the 1^st^ millennium BC, and 5) the cemetery yielding 3^rd^ millennium BC burials.

**Fig 2:**
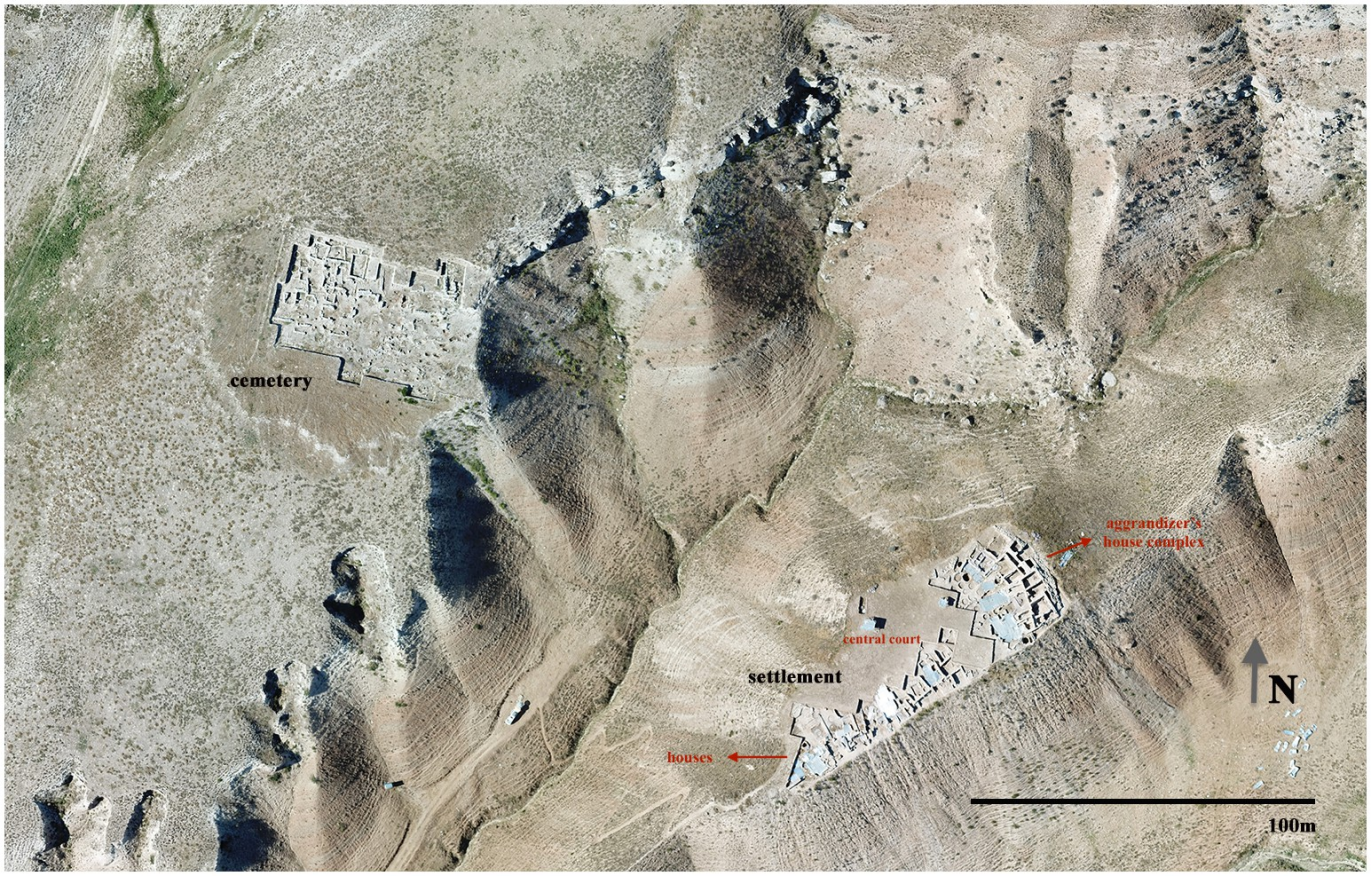
The late 3^rd^ millennium BC settlement and cemetery of Resuloğlu (©Resuloğlu excavations archive).

This suite of sites covers a long period from the late 4^th^ to the 1^st^ millennia BC, but any estimation of the territorial extent of Resuloğlu at a certain period seems difficult. Its greatest size was reached during the early 1^st^ millennium BC and is estimated at 5 ha. The interruption in chronology and the fragmentary nature of archaeological evidence at the 4^th^ millennium BC northwest mound and 1^st^ millennium BC layers of the southeast mound prompted intensive research at the 3^rd^ millennium BC layers of the southeast mound and the cemetery, which only covers the latter half of the period. Regarding the completion of the data, this research focuses only on this settlement and its contemporary cemetery (Fig 2). Therefore, what is referred hereafter as Resuloğlu is essentially the late 3^rd^ millennium BC levels of the southeast mound.

The settlement dates to ca. 2500/2400–2100/2050 BC with relative chronology. Radiocarbon samples collected from different rooms and silos verify this interval (Table 1).

**Table 1:** Radiocarbon results of charcoal and carbonized grain samples from the Resuloğlu settlement. The analysis has been conducted at ETH Zurich and the Sarayköy Atomic Energy Institute in Ankara.

Sample number	Sample type	^14^C result–1σ cal. BC	^14^C result–2σ cal. BC
ETH-42014 DK-10/101	Charcoal	2470–2290	2470–2340
ZT 34. RO.12 Room 1	Carbonized grains	2286–2042	2455–1975
Room K (village-head’s house complex)	Charcoal	2473–2235	2570–2195

**Fig 3:**
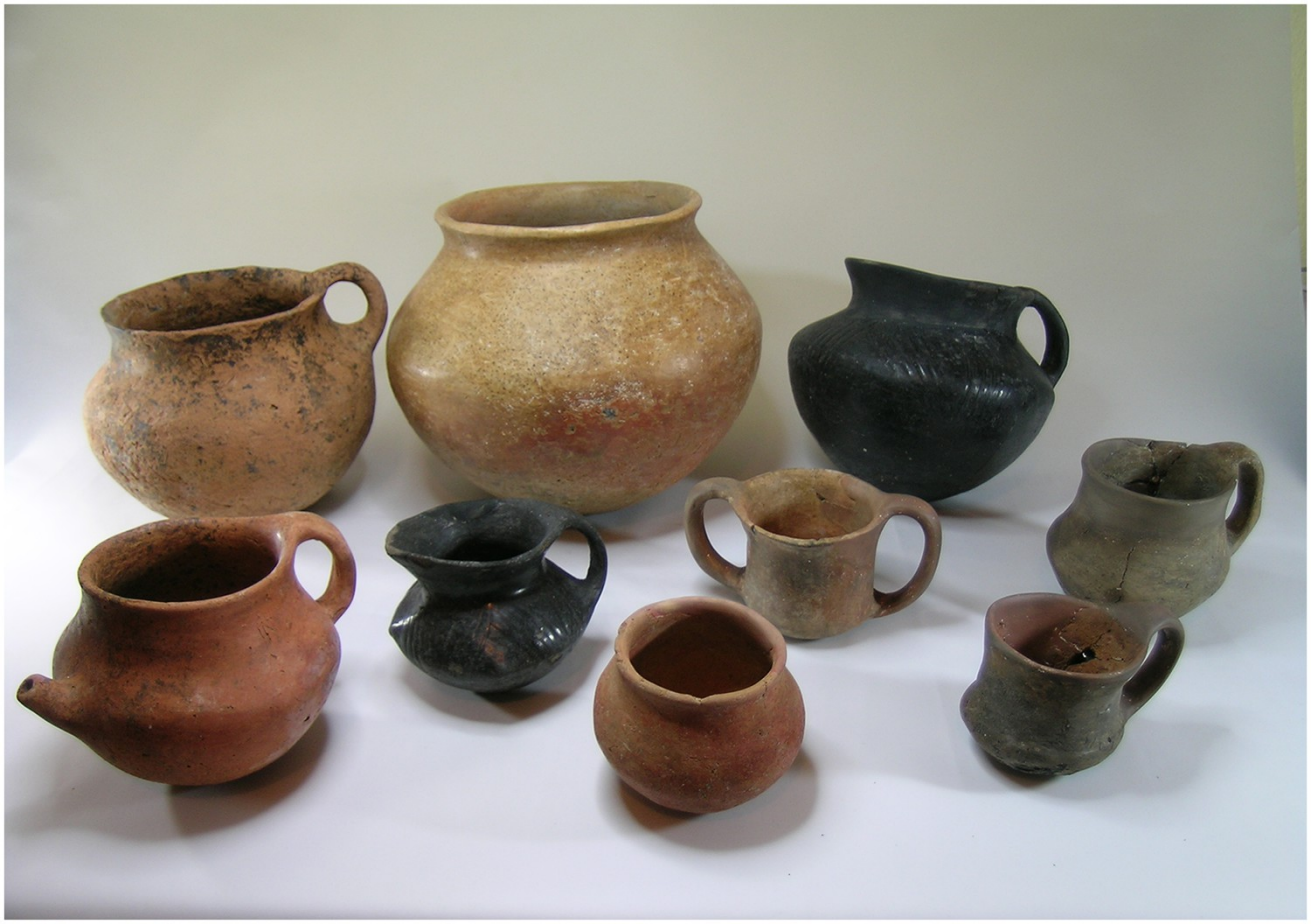
Examples from the local 3^rd^ millennium BC ceramics from Resuloğlu (©Resuloğlu excavations archive).

Two sub-phases, broadly identified as a late and an early phase, are distinguished based on ceramic and material evidence (Fig 3). Radiocarbon results from the later phase confirm the dating of 2200–2150 BC. The cemetery area is contemporaneous with the settlement based on the material evidence recovered as parts of burial gifts. Based on the relative and absolute dating evidence, we can confidently propose a maximum occupational history of 400 years to the late 3^rd^ millennium levels of Resuloğlu.

### The settlement

Resuloğlu lies on the flat plain of a small hilltop in the hilly landscape of the central Pontides. The site overlooks the Delice River and Valley near the Kızılırmak River, the ancient Halys. The hilltop position provides the site with control over the valley from a secure standpoint. Additionally, the location connects well with both inner central Anatolia to the south and the Black Sea to the north. It also gives access towards Ankara at the west via a range of pathways. While this hilltop location presents a strategic advantage over the landscape and networks, it limits the size of the settlement to 0.35 ha [29: p. 587]. The cemetery covers 0.26 ha and is located on an opposite ridge with a separation of approximately 100–120 m today. The cemetery and the settlement might have been once connected. However, due to heavy erosion in this badland area, a cleft currently separates them.

**Fig 4:**
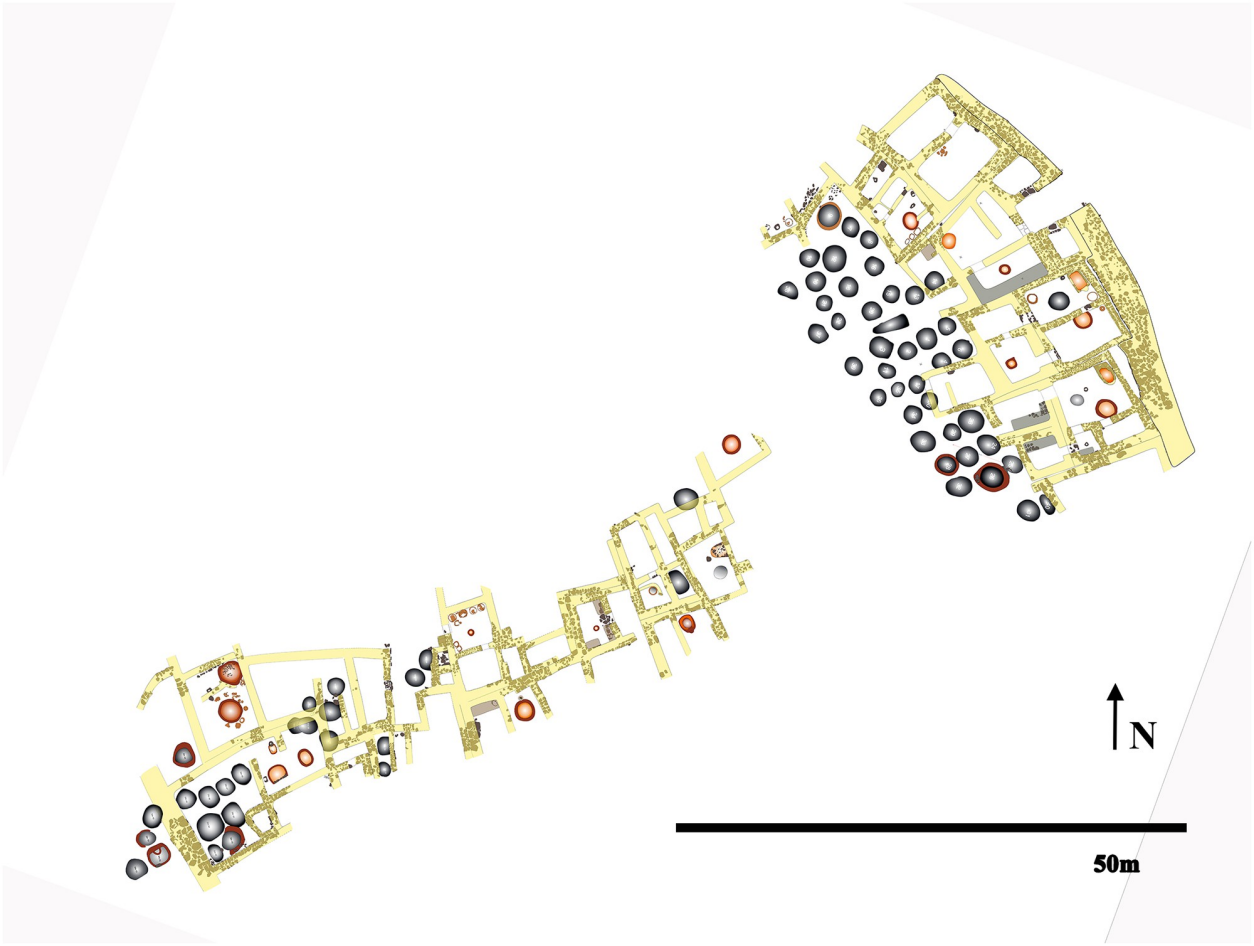
Architectural plan of the Resuloğlu settlement (©Resuloğlu excavations archive).

The settlement provides invaluable information to investigate the site’s social complexity. Fortification walls and domestic architecture with three- to four-roomed houses that formed one- or two-story houses were found (Fig 4). At the southern end of the settlement, excavations yielded a 22-room house complex; with its own fortification and 30 silos of approximately 80 tons of storage [29, 31]. The materials found in this complex did not differ from the other houses. The excavation director Yıldırım [30] defined this sector as the residence of a chief; the presence of an aggrandizing individual based on the physical separation of the sector from the rest of the site. The labor invested in building fortification walls for the settlement and the multi-roomed complex along with massive silos should be encountered as a local-group level organization.

The material culture uncovered in the domestic structures includes local pottery, utilitarian tools of stone and bone, animal bones, and a few metal objects (Fig 3). Even though archaeological evidence suggests that textile production was part of the household economy, agricultural production stands as the pillar of Resuloğlu’s economy. A significant architectural feature is a total of 64 circular silos with a capacity of 220 tons of grain [32] uncovered in association with or around the domestic houses (Fig 2). Stamp seals made of local stones (with only one example made of arsenical copper) were commonly found on the silo floors indicative of a certain level of control and ownership.

### The cemetery

**Fig 5:**
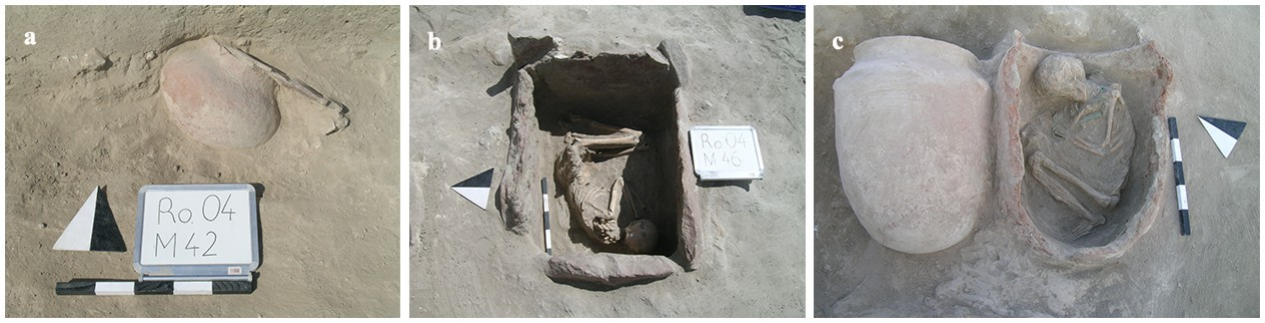
Examples from burial practices in the Resuloğlu cemetery: a) jar grave, b) cist grave, c) pithos grave (©Resuloğlu excavations archive).

The cemetery consisted of 288 graves in four burial types: pithos, cist, jar, and simple inhumation (Fig 5). The majority of the graves were pithoi (Fig 5c). Burial gifts included ceramics, metal items, and beads made in various media [33, 34]. Funeral banquets appeared as a rare but known practice [13]. Cattle skulls or hooves left inside and outside of the graves showed similarities with the well-known examples from the graves at Alaca Höyük.

Types of burial gifts are similar to those found in domestic contexts at the settlement. Similar goods are widely known throughout the Halys Basin [33: p.8, Fig 11; 34]. The majority of the graves contain metal artifacts. The metal grave goods display significant variation in terms of artifact typology. Jewelry such as bracelets, anklets, earrings, hair rings, beads, and torques are frequent. Pins are the most common type. Almost every burial contains at least one pin, possibly used to bind a cerement. Some burials were richer in numbers of metal objects, and some contain rare items like cups or daggers.

### Social structure: Where are the elites?

Resuloğlu’s village-head must be understood as an individual seeking power over desirable resources [35]. The capacity of the silos in this complex confirms this suggestion. Recent research predicted the total population of the settlement at 115 people [29: p.587], whose grain consumption would be approximately 40 tons [36, 37]. The existence of silos with a total capacity of 220 tons indicates that the population had stored almost an extra 180 tons of grain. It is possible that all the silos might not have been filled to full capacity every year due to natural fluctuations (i.e., precipitation) or other environmental perturbations (e.g., salinization). While these risks stand as reasons for a decrease in the production capacity, good seasons might well end with better yields of harvest. In a worst-case scenario, Resuloğlu must have had at least 100 tons of grain, more than twice that they needed for exchange, confirming one of the subsistence economies as agriculture.

Resuloğlu demonstrates a nonstate, agricultural society with an expression of hierarchy and egalitarianism that confirms its middle-range society concept. The archaeological and architectural remains at the settlement area support this. The existence of a separate residential area indicates a certain level of group hierarchy. However, defining the group settling in this private quarter as elites would be an overstatement. Silos in common areas, similarity in domestic house plans, and equality of material (e.g., stone tools, ceramics) in the house contexts designate a certain level of heterarchy. Archaeological evidence from the cemetery will support this.

Social differentiation has its roots in economically based power [38: p.56]. At Resuloğlu, the primary subsistence is derived from agriculture, textile weaving, and husbandry. The household consumption of metals is limited to 12 utilitarian tools; the majority of tools were made of stone. The overarching consumption of metals as grave goods indicates that the surplus of staple commodities must have been exchanged or traded for metals, most likely to a certain extent under the control of the village-head. The variety in types and composition of metal grave goods demonstrates the extent of this give-and-take or trade system.

Taking the diversity of metal artifacts (e.g., axes, daggers, cups, beads, etc.) as a proxy of the varying personal status of individuals, such differences would imply a certain degree of social differentiation. For example, the only mace head from the site was recovered in a pithos grave belonging to an elderly female [39]. The rare occurrence of funerary banquets involving cattle skulls or hooves demonstrates the use of display in the burial rituals, which could be interpreted as a way to emphasize social status [40]. Still, there is no solid evidence to assign certain burials to a leader or socially high-ranked persona. There is a certain degree of inequality; however, associating the metal-rich graves with the existence of elites would not fit the archaeological evidence from the domestic contexts either.

## Research context and materials

The existing literature on the north-central Anatolian archaeometallurgy shows no doubt about the quality and quantity of metal artifacts in the region signaling the wealth and advanced technological skills of the ancient crafts. However, prestigious, symbolic, or exceptional artifacts have given priority to scientific analysis mostly overlooking utilitarian objects. Additionally, not much has been proposed regarding the socioeconomic system supporting such accumulation.

The existence of tin (>1 wt%), occasionally as high as ca. 20 wt%, on the so-called prestigious and symbolic corpora of metal grave goods, has fueled debates on the sources of tin [41, 42, with references]. Since the 1980s, this venue of debate–known as the ‘tin problem’ in the literature–has questioned whether tin was available in Anatolia. Today, the presence of Anatolian tin is unquestionable. Cassiterite, the tin oxide mineral (SnO_2_), is available in Kestel (Niğde) and Hisarcık (Kayseri) as minor occurrences. These should have been available to ancient miners for a long time [42–45]. While Kestel’s tin was exploited and used during the 3^rd^ millennium BC in its associated mining settlement of Göltepe [42, 43], archaeological evidence is pending for the systematic exploitation and use of Hisarcık’s tin.

The ostentatious metal corpora of the region have also prompted discussions on the identity of the craftspeople who were skillfully manufacturing such unique pieces. Local and regional schools for metalwork have been suggested for this preliterate period of Anatolia [46, 47]. Certain zones with rich polymetallic sources were proposed as suitable for extensive metal production [24]. Hattians, the local population of the Halys Basin during the second half of the 3^rd^ millennium BC, have been suggested as skillful artisans of metalwork. Any discussions of the context of metal production or specialization will be inadequate, due to the archaeological context of these final products as grave goods [48: p. 154–155; 49]. Thus, this study does not propose any ideas in the context of metal production and specialization.

The systematic archaeological and archaeometric research conducted at Resuloğlu is unique in the sense of non-selective sampling. The first aim is to understand the metals used, alloy compositions, and the likely provenance of metals. Secondly, the investigation targets to detect any possible variations between the metal choices for the cemetery and the settlement. The third objective is to trace metal flow through trading networks. The ultimate goal of this study is to illuminate the life stories of metals in their socio-economic context.

**Fig 6:**
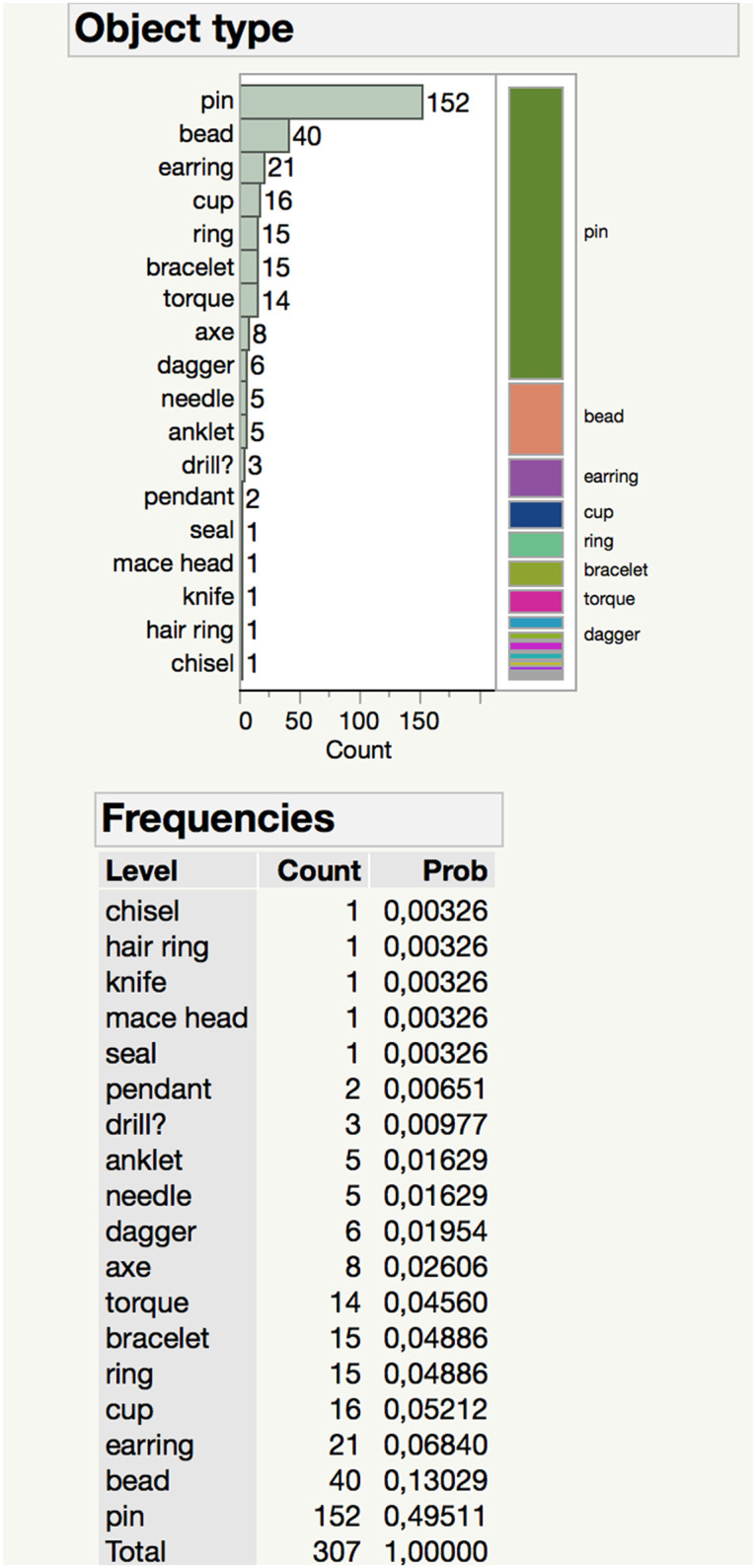
Distribution of artifacts according to typologies.

Almost two decades of excavations at Resuloğlu have yielded approximately 400 metal objects and fragments. While beads constitute the majority of the assemblage, their fragile nature prevented conducting analytical research on many of them. 18 different artifact groups consisting of 152 pins, 40 beads, 21 earrings, 16 cups, 15 rings, 15 bracelets, 14 torques, 8 axes, 6 daggers, 5 needles, 5 anklets, 3 drills, 2 pendants, a seal, a mace head, a knife, a hair ring, and a chisel were analyzed in this study (Fig 6). The detailed information on the samples is listed in Table 2. All necessary permits were obtained for the described study, which complied with all relevant regulations from the Turkish Ministry of Culture and Tourism and the Çorum Museum.

**Table 2:** The inventory numbers (excavation and/or museum numbers), object types, analysis point(s), typologies (where applicable), weighs, and alloy types of the Resuloğlu metal artifacts. (nd: not determined or inventory number not assigned; nm: weight not measured).

Obj.	Excavation number	Museum Inventory number	Object type	Context	Analysis point	Typology (where applicable)/Notes	Weigh	Alloy type
**1**	2017	nd	seal	settlement	body	square, geometric design	nm	Cu-As
**2**	6_15_2004	7516	pin	cemetery	body		6.75	Cu-Pb
**3**	8_31_2005	7703	dagger	cemetery	blade		77.9	Cu-Sn
**4**	10_10_2003	7405	pin	cemetery	head, shaft		18.8	Cu-As
**5**	10_19_2003	7414	bracelet	cemetery	body		127.3	Cu-As-Sn
**6**	10_20_2003	7415	bracelet	cemetery	body		122.6	Cu-Sn
**7**	Ro.06/Etd.30	1064_1	bracelet	cemetery	body		117.95	Cu
**8**	Ro.06/Etd.30	1064_2	bracelet	cemetery	body		122.9	Cu-Sn
**9**	14_1_2008	8655	pin	cemetery	head, shaft		12.1	Cu-As
**10**	14_2_2008	8656	pin	cemetery	head, shaft		4.1	Cu-As
**11**	14_2_2009	8908	pin	cemetery	head, shaft		22.8	Cu-Sn
**12**	14_3_2008	8657	pin	cemetery	head, shaft		22.5	Cu-Sn
**13**	14_5_2009	8911_1	bead	cemetery	body	tubular	nm	Cu-Sn
**14**	14_5_2009	8911_2	bead	cemetery	body	tubular	nm	Cu-Sn
**15**	14_6_2008	8660_1	bead	cemetery	body	disc	nm	Cu-Sn
**16**	14_6_2008	8660_2	bead	cemetery	body	disc	nm	Cu-Sn
**17**	14_6_2008	8660_3	bead	cemetery	body	tubular	nm	Cu-Sn
**18**	18_1_2007	8614_1	earring	cemetery	body		total 2.15	Cu-Ag-Au-Sb
**19**	18_1_2007	8614_2	earring	cemetery	body		total 2.15	Cu-Ag-Au
**20**	18_12_2007	8625	earring	cemetery	gold leaf coating	stone with Au coating	nm	Au-Ag
**21**	18_25_2007	8638	pin	cemetery	head, shaft		16.25	Cu-As-Sn
**22**	18_26_2007	8639	pin	cemetery	head, shaft		7.95	Cu-Sn
**23**	18_3_2007	8616	pin	cemetery	head		13.8	Cu-Sn
**24**	18_3_2007	8616	pin	cemetery	shaft		13.8	Cu-Sn-Pb
**25**	18_4_2007	8617	pin	cemetery	head, shaft		6.3	Cu-As
**26**	6_11_2004	7512_1	bead	cemetery	body		nm	Cu-Sn
**27**	6_11_2004	7512_2	bead	cemetery	body		nm	Cu-Sn
**28**	6_12_2004	7513	pin	cemetery	head		17.35	Cu
**29**	6_12_2004	7513	pin	cemetery	shaft		17.35	Cu-As-Sn
**30**	6_17_2004	7518	pin	cemetery	head, shaft		10.7	Cu-Sn
**31**	6_18_2004	7519	pin	cemetery	head, shaft		17.2	Cu-Sn
**32**	6_21_2004	7522_1	bead	cemetery	body	disc	nm	Cu-Sn
**33**	6_21_2004	7522_2	bead	cemetery	body	disc	nm	Cu-Sn
**34**	6_24_2004	7525	pin	cemetery	head, shaft		13.75	Cu-Sn
**35**	6_26_2004	7527	pin	cemetery	head		12.8	Cu-Sn
**36**	6_26_2004	7527	pin	cemetery	shaft		12.8	Cu
**37**	6_4_2004	7505_1	bead	cemetery	body	disc	nm	Cu-Sn
**38**	6_4_2004	7505_2	bead	cemetery	body	disc	nm	Cu-Sn
**39**	6_4_2004	7505_3	bead	cemetery	body	tubular	nm	Cu-Sn
**40**	6_4_2004	7505_4	bead	cemetery	body	disc	nm	Cu-Sn
**41**	6_8_2004	7509	pin	cemetery	head, shaft		15.05	Cu-As
**42**	6_20_2004	7521	pin	cemetery	head, shaft		11.2	Cu-Sn
**43**	6_29_2004	7530	axe	cemetery	body, blade		174.7	Cu-As
**44**	nd	7602	pin	cemetery	head, shaft		nm	Cu-Sn
**45**	8_10_2005	7682	pin	cemetery	head, shaft		16.3	Cu-Sn
**46**	8_12_2005	7684	pin	cemetery	head, shaft		9.45	Cu-As
**47**	9_13_2006	7897_1	earring	cemetery	head		2	Cu-Ag-Au
**48**	9_13_2006	7897_2	earring	cemetery	head		2	Cu-Ag-Au
**49**	9_16_2006	7900	pin	cemetery	head		36.9	Cu-As-Sn
**50**	9_18_2006	7902	cup	cemetery	body		42	Cu-Ag
**51**	9_24_2006	7908_1	bead	cemetery	body	swastika	nm	Au-Ag-As
**52**	9_24_2006	7908_2	bead	cemetery	body	tubular	nm	Cu-Ag-Au
**53**	9_24_2006	7908_3	bead	cemetery	body	swastika	nm	Au-Ag-As
**54**	9_24_2006	7908_4	bead	cemetery	body	tubular	nm	Cu-Ag-Au
**55**	8_21_2005	7693	pin	cemetery	head, shaft		8.8	Cu-As
**56**	8_25_2005	7697_1	anklet	cemetery	body		15.6	Cu-Sn
**57**	8_25_2005	7697_2	anklet	cemetery	body		26.05	Cu-Sn-Pb
**58**	8_27_2005	7699_1	ring	cemetery	body		total 4.70	Cu-Ag
**59**	8_27_2005	7699_2	ring	cemetery	body		total 4.70	Cu-Ag
**60**	8_29_2005	7701	torque	cemetery	body		11.1	Cu-As-Ag
**61**	8_32_2005	7704_1	earring	cemetery	head	earring_1	total 5.60	Cu-Ag
**62**	8_32_2005	7704_2	earring	cemetery	head	earring_2	total 5.60	Cu-Ag
**63**	8_8_2005	7680_1	bead	cemetery	body	tubular	nm	Cu-Sn
**64**	8_8_2005	7680_2	bead	cemetery	body	tubular	nm	Cu-Sn
**65**	8_9_2005	7681	pin	cemetery	head		8.4	Cu-As
**66**	8_9_2005	7681	pin	cemetery	shaft		8.4	Cu-Sn
**67**	9_17_2006	7901	mace head	cemetery	body		178.95	Cu
**68**	9_26_2006	7910	cup	cemetery	body		121.45	Cu-Sn
**69**	9_6_2011	9104	pin	settlement	head		6.95	Cu-As
**70**	9_6_2011	9104	pin	settlement	shaft		6.95	Cu
**71**	9_7_2011	9105	pin	cemetery	head		6.25	Cu
**72**	9_7_2011	9105	pin	cemetery	shaft		6.25	Cu-As
**73**	9_8_2006	7892	pin	cemetery	head		13.65	Cu-As
**74**	9_8_2006	7892	pin	cemetery	shaft		13.65	Cu-As-Sn
**75**	9_8_2011	9107	pin	settlement	shaft		6.6	Cu-As
**76**	nd	Etd_1	axe	cemetery	blade, ridge		>300	Cu-Sn
**77**	Ro.05/Etd.3	Etd_1002	pin	cemetery	head, shaft		4.35	Cu-As
**78**	Ro.05/Etd.4	Etd_1003	pin	cemetery	shaft		8.75	Cu-Sn
**79**	Ro.05/Etd.5	Etd_1004	pin	cemetery	shaft		8.6	Cu-Sn
**80**	Ro.05/Etd.6	Etd_1005	pin	cemetery	shaft		5.6	Cu-As
**81**	Ro.05/Etd.7	Etd_1006	cup	cemetery	body	fragment	17.85	Cu-Pb
**82**	Ro.05/Etd.8	Etd_1007	needle	cemetery	shaft		4.1	Cu-Sn
**83**	Ro.05/Etd.9	Etd_1008	torque	cemetery	body		15.25	Cu-Sn
**84**	Ro.05/Etd.10	Etd_1009	pin	cemetery	shaft		2.05	Cu-As
**85**	Ro.05/Etd.11	Etd_1011	ring	cemetery	body		nm	Cu-Ag
**86**	Ro.05/Etd.14	Etd_1013	pin	cemetery	shaft		14.3	Cu-Sn
**87**	Ro.05/Etd.16	Etd_1015	pin	cemetery	shaft		7.3	Cu-Sn
**88**	Ro.05/Etd.18	Etd_1017	torque	cemetery	body		6.25	Cu-Sn
**89**	Ro.05/Etd.19	Etd_1018	earring	cemetery	gold leaf coating		nm	Cu-As-Ag-Au
**90**	Ro.05/Etd.21	Etd_1020	bead	cemetery	body	nm	nm	Cu-Sn
**91**	Ro.05/Etd.22	Etd_1021	ring	cemetery	body		18.8	Cu-Sn
**92**	Ro.05/Etd.23	Etd_1022	cup	cemetery	body	fragment	35.5	Cu-Sn
**93**	Ro.05/Etd.24	Etd_1023	pin	cemetery	shaft		22.1	Cu-Sn
**94**	Ro.05/Etd.26	Etd_1025	torque	cemetery	body		97.25	Cu-Sn
**95**	Ro.05/Etd.27	Etd_1026	ring	cemetery	body		75.95	Cu-Sn
**96**	Ro.05/Etd.28	Etd_1027_1	bracelet	cemetery	body		79.95	Cu-Sn
**97**	Ro.05/Etd.28	Etd_1027_2	bracelet	cemetery	body		63.9	Cu-Sn
**98**	Ro.05/Etd.29	Etd_1028	pin	cemetery	shaft		7.2	Cu-Sn
**99**	Ro.05/Etd.31	Etd_1030	torque	cemetery	body		66.7	Cu-Sn
**100**	Ro.05/Etd.32	Etd_1031	dagger	cemetery	blade		10.15	Cu-As
**101**	Ro.05/Etd.34	Etd_1033	pin	cemetery	shaft		2.8	Cu
**102**	Ro.07/Etd.1	Etd_1079	torque	cemetery	body		47.25	Cu-Sn
**103**	Ro.07/Etd.2	Etd_1080_1	bracelet	cemetery	body		39.75	Cu
**104**	Ro.07/Etd.2	Etd_1080_2	bracelet	cemetery	body		47.3	Cu-Sn
**105**	Ro.07/Etd.3	Etd_1081	bead	cemetery	body	tubular	nm	Cu-Sn
**106**	Ro.07/Etd.4	Etd_1082	axe	cemetery	blade		9.1	Cu-As
**107**	Ro.07/Etd.5	Etd_1083	ring	cemetery	body		2.65	Cu-As-Ag
**108**	Ro.07/Etd.6	Etd_1084	pin	cemetery	shaft		9.1	Cu-As
**109**	Ro.07/Etd.7	Etd_1085	pin	cemetery	head		7.5	Cu-Sn
**110**	Ro.07/Etd.8	Etd_1086	bead	cemetery	body	tubular	nm	Cu-As
**111**	Ro.07/Etd.9	Etd_1087	earring	cemetery	head		4.0	Cu-Sn
**112**	Ro.07/Etd.10	Etd_1088_1	earring	cemetery	head		total 2.85	Cu-Sn
**113**	Ro.07/Etd.10	Etd_1088_2	earring	cemetery	head		total 2.85	Cu-As-Sb
**114**	Ro.07/Etd.11	Etd_1089	pin	cemetery	shaft		4.55	Cu-Sn
**115**	Ro.07/Etd.13	Etd_1091	pin	cemetery	shaft		17.8	Cu-Sn
**116**	Ro.07/Etd.14	Etd_1092	pin	cemetery	head		22.85	Cu-Sn
**117**	Ro.07/Etd.15	Etd_1093	pin	cemetery	shaft		11.15	Cu-Sn
**118**	Ro.07/Etd.16	Etd_1094	pin	cemetery	shaft		4.7	Cu-Sn
**119**	Ro.07/Etd.17	Etd_1095	pin	cemetery	shaft		10.3	Cu-Sn
**120**	Ro.07/Etd.18	Etd_1096_1	bead	cemetery	body	tubular	nm	Cu-Sn
**121**	Ro.07/Etd.18	Etd_1096_2	bead	cemetery	body	tubular	nm	Cu-Sn
**122**	Ro.07/Etd.18	Etd_1096_3	bead	cemetery	body	ring	nm	Cu-Sb
**123**	Ro.07/Etd.18	Etd_1096_4	bead	cemetery	body	disc	nm	Cu-Sn
**124**	Ro.07/Etd.19	Etd_1097	pin	cemetery	head		22.25	Cu-Sn
**125**	Ro.07/Etd.20	Etd_1098	pin	cemetery	head	vase- headed	31.0	Cu-Sn
**126**	Ro.07/Etd.21	Etd_1099	pin	cemetery	head		26.85	Cu-Sn
**127**	M132	Etd_11	pin	cemetery	head		15.75	Cu-Sn
**128**	Ro.07/Etd.22	Etd_1100	cup	cemetery	body	fragment		Cu-Sn
**129**	Ro.07/Etd.23	Etd_1101	bracelet	cemetery	body		29.9	Cu-Sn
**130**	Ro.07/Etd.24	Etd_1102	bracelet	cemetery	body		10.55	Cu
**131**	Ro.07/Etd.25	Etd_1103	anklet	cemetery	body		125.4	Cu-Sn
**132**	Ro.07/Etd.27	Etd_1105	pin	cemetery	shaft		13.4	Cu-Sn
**133**	Ro.07/Etd.28	Etd_1106	pin	cemetery	head, shaft		16.85	Cu-Sn
**134**	Ro.07/Etd.29	Etd_1107	pin	cemetery	shaft		14.2	Cu-Sn
**135**	Ro.07/Etd.30	Etd_1108	pin	cemetery	shaft, head		11.8	Cu-As-Sn
**136**	Ro.07/Etd.32	Etd_1110	ring	cemetery	body		58.3	Cu-Sn
**137**	Ro.07/Etd.33	Etd_1111	axe	cemetery	body		147.9	Cu-As
**138**	Ro.07/Etd.34	Etd_1112	dagger	cemetery	blade		>300	Cu-Sn
**139**	Ro.07/Etd.35	Etd_1113	bead	cemetery	body	tubular	total of all beads in this inventory 4.45	Cu-Sn
**140**	Ro.07/Etd.36	Etd_1114	pin	cemetery	head		6.85	Cu-Sn
**141**	Ro.07/Etd.37	Etd_1115	torque	cemetery	body		30.2	Cu-Sn
**142**	Ro.07/Etd.38	Etd_1116	pin	cemetery	shaft		11.85	Cu-Sn
**143**	Ro.07/Etd.39	Etd_1117	pin	cemetery	head		2.65	Cu-As
**144**	Ro.07/Etd.40	Etd_1118	pin	cemetery	head		6.25	Cu-As
**145**	Ro.07/Etd.41	Etd_1119	bead	cemetery	body	tubular	nm	Cu-Sn
**146**	Ro.07/Etd.42	Etd_1120	pin	cemetery	shaft		12.65	Cu-Sn
**147**	Ro.07/Etd.43	Etd_1121	bracelet	cemetery	body		51.2	Cu-As-Sn
**148**	Ro.08/Etd.1	Etd_1126_1	anklet	cemetery	body		196.05	Cu-Sn
**149**	Ro.08/Etd.1	Etd_1126_2	anklet	cemetery	body		185.0	Cu-Sn
**150**	Ro.08/Etd.2	Etd_1127_1	bracelet	cemetery	body		54.5	Cu-Sn
**151**	Ro.08/Etd.2	Etd_1127_2	bracelet	cemetery	body		51.95	Cu-Sn
**152**	Ro.08/Etd.3	Etd_1128_1	pin	cemetery	head		total of 10 pins in this inventory 292.65	Cu
**153**	Ro.08/Etd.3	Etd_1128_1	pin	cemetery	shaft		total of 10 pins in this inventory 292.65	Cu-Sn
**154**	Ro.08/Etd.3	Etd_1128_2	pin	cemetery	shaft		total of 10 pins in this inventory 292.65	Cu-Sn
**155**	Ro.08/Etd.3	Etd_1128_3	pin	cemetery	shaft		total of 10 pins in this inventory 292.65	Cu-Sn
**156**	Ro.08/Etd.4	Etd_1129	pin	cemetery	head		5.35	Cu-As
**157**	Ro.08/Etd.5	Etd_1130	needle	cemetery	shaft		1.9	Cu-As
**158**	Ro.08/Etd.6	Etd_1131	pin	cemetery	head, shaft		6.25	Cu-Sn
**159**	Ro.08/Etd.7	Etd_1132	pin	cemetery	shaft		7.95	Cu-Sn
**160**	Ro.08/Etd.8	Etd_1133	pin	cemetery	shaft		7.8	Cu-Sn
**161**	Ro.08/Etd.9	Etd_1134	pin	cemetery	shaft		3.7	Cu-As
**162**	Ro.08/Etd.10	Etd_1135	pin	cemetery	head, shaft		9.05	Cu-Sn
**163**	Ro.08/Etd.11	Etd_1136	pin	cemetery	head, shaft		13.55	Cu-Sn
**164**	Ro.08/Etd.12	Etd_1137	pin	cemetery	head, shaft		19.2	Cu-Sn
**165**	Ro.08/Etd.13	Etd_1138	torque	cemetery	body		19.65	Cu-Sn
**166**	Ro.08/Etd.14	Etd_1139	dagger	cemetery	blade		25.95	Cu-Sn
**167**	Ro.08/Etd.15	Etd_1140	knife	cemetery	blade		20.15	Cu-Sn
**168**	Ro.08/Etd.16	Etd_1141	axe	cemetery	blade		120.1	Cu-As
**169**	Ro.08/Etd.17	Etd_1142	pin	cemetery	head		14.4	Cu-Sn
**170**	Ro.08/Etd.18	Etd_1143	cup	cemetery	body		48.55	Cu-Sn
**171**	Ro.08/Etd.19	Etd_1144	pin	cemetery	shaft		19.1	Cu-Sn
**172**	Ro.08/Etd.20	Etd_1145	pin	cemetery	shaft		6.5	Cu-Sn
**173**	Ro.08/Etd.21	Etd_1146	pin	cemetery	head		7.7	Cu-As
**174**	Ro.08/Etd.22	Etd_1147	pin	cemetery	shaft		3.7	Cu-As
**175**	Ro.08/Etd.23	Etd_1148	torque	cemetery	body		25.5	Cu-Sn
**176**	Ro.08/Etd.24	Etd_1149	torque	cemetery	body		40.5	Cu-Sn
**177**	Ro.08/Etd.25	Etd_1150	earring	cemetery	head		2.9	Cu
**178**	Ro.08/Etd.26	Etd_1151_1	ring	cemetery	body		total 2,3	Ag
**179**	Ro.08/Etd.26	Etd_1151_2	ring	cemetery	body		total 2.3	Cu-Sn
**180**	Ro.08/Etd.27	Etd_1152	ring	cemetery	body		5.25	Cu-As
**181**	Ro.08/Etd.28	Etd_1153_1	bead	cemetery	body	disc	total of all beads in this inventory 40.75	Cu-Sn
**182**	Ro.08/Etd.28	Etd_1153_2	bead	cemetery	body	barrel	total of all beads in this inventory 40.75	Cu-Sn
**183**	Ro.08/Etd.28	Etd_1153_3	bead	cemetery	body	tubular	total of all beads in this inventory 40.75	Cu-Sn
**184**	Ro.08/Etd.29	Etd_1154	bead	cemetery	body	disc	nm	Cu-Sn
**185**	Ro.08/Etd.30	Etd_1155	earring	cemetery	head		5.35	Cu-As-Ag-Au
**186**	Ro.08/Etd.30	Etd_1155	earring	cemetery	shaft		5.35	Cu-As-Au
**187**	Ro.08/Etd.31	Etd_1156	bead	cemetery	body	Ur-type	nm	Cu-Ag-Au
**188**	Ro.08/Etd.47	Etd_1172	drill?	cemetery	shaft		4.55	Cu-As
**189**	Ro.08/Etd.48	Etd_1173	cup	cemetery	body		26.75	Cu-Sn
**190**	Ro.09/Etd.21	Etd_1194	earring	cemetery	head		2.8	Au-As
**191**	Ro.09/Etd.22	Etd_1195	earring	cemetery	gold leaf coating		0.1	Cu-As-Ag-Au
**192**	Ro.09/Etd.23	Etd_1196	pin	cemetery	shaft		18.7	Cu-As-Sn
**193**	Ro.09/Etd.25	Etd_1198	pin	cemetery	head		15.0	Cu-Sn
**194**	Ro.09/Etd.26	Etd_1199	pin	cemetery	head		8.4	Cu-As
**195**	Ro.06/M137	Etd_12	pin	cemetery	shaft		3.6	Cu
**196**	Ro.09/Etd.27	Etd_1200	pin	cemetery	head		21.55	Cu-Sn
**197**	Ro.09/Etd.28	Etd_1201	pin	cemetery	head		6.35	Cu-Sn
**198**	Ro.09/Etd.29	Etd_1202	pin	cemetery	shaft		9.65	Cu-Sn
**199**	Ro.09/Etd.30	Etd_1203	pin	cemetery	shaft		11.9	Cu-Sn
**200**	Ro.09/Etd.31	Etd_1204	torque	cemetery	shaft, this has Ca		14.9	Cu
**201**	Ro.09/Etd.32	Etd_1205	pin	cemetery	shaft		10.9	Cu-Sn
**202**	Ro.09/Etd.33	Etd_1206_1	pin	cemetery	shaft		total 45.75	Cu-Sn
**203**	Ro.09/Etd.33	Etd_1206_2	torque	cemetery	body		total 45.75	Cu-Sn-Pb
**204**	Ro.09/Etd.34	Etd_1207	pin	cemetery	shaft		11.9	Cu-Sn
**205**	Ro.09/Etd.35	Etd_1208	dagger	cemetery	body		10.65	Cu-As
**206**	Ro.09/Etd.36	Etd_1209	pin	cemetery	shaft		10.75	Cu-As
**207**	Ro.09/Etd.38	Etd_1211	ring	cemetery	shaft		1.15	Cu-As
**208**	Ro.09/Etd.39	Etd_1212	pin	cemetery	shaft		3.15	Cu-As-Sn
**209**	Ro.09/Etd.40	Etd_1213	pin	cemetery	shaft		4.0	Cu-As-Sn
**210**	Ro.09/Etd.41	Etd_1214	needle	cemetery	shaft		2.6	Cu-As
**211**	Ro.09/Etd.42	Etd_1215	needle	cemetery	shaft		1.7	Cu-As
**212**	Ro.09/Etd.43	Etd_1216	bead	cemetery	shaft	disc	1.25	Cu-As-Sn
**213**	Ro.09/Etd.45	Etd_1218	pin	cemetery	shaft		10.05	Cu-Sn
**214**	Ro.09/Etd.46	Etd_1219	needle	cemetery	shaft		1.9	Cu-Sn
**215**	Ro.09/Etd.63	Etd_1235	dagger	cemetery	blade		48.75	Cu-As-Sn
**216**	Ro.09/Etd.64	Etd_1236	cup	cemetery	body		31.0	Cu-Sn
**217**	Ro.09/Etd.65	Etd_1237	cup	cemetery	body		nm	Cu-Sn
**218**	Ro.09/Etd.66	Etd_1238	pin	cemetery	shaft		7.3	Cu-Sn
**219**	Ro.06/M138	Etd_13	pin	cemetery	shaft		32.30	Cu-Sn
**220**	Ro.06/M138	Etd_14	pin	cemetery	shaft		26.2	Cu-Sn
**221**	Ro.06/M138	Etd_15	pin	cemetery	shaft		9.35	Cu-Sn
**222**	Ro.06/M38	Etd_16	bead	cemetery	body	disc	nm	Cu-Sn
**223**	Ro.06/M140	Etd_17	pin	cemetery	shaft		9.75	Cu
**224**	Ro.06/M140	Etd_18	pin	cemetery	shaft, head		14.0	Cu-As-Sn
**225**	Ro.06/M120	Etd_2	pin	cemetery	head		10.45	Cu-Sn
**226**	Ro.06/M142	Etd_20	torque	cemetery	body		36.35	Cu-Sn
**227**	nd	Etd_2003_1	cup	cemetery	body	fragment	nm; partially oxidized	Cu-As
**228**	nd	Etd_2003_11	pin	cemetery	shaft		nm; partially oxidized	Cu-Sn
**229**	nd	Etd_2003_12	pin	cemetery	shaft		nm; partially oxidized	Cu-Sn
**230**	nd	Etd_2003_14	pin	cemetery	shaft		nm; partially oxidized	Cu-Sn-Pb
**231**	nd	Etd_2003_2	pin	cemetery	shaft		nm; partially oxidized	Cu-Sn
**232**	nd	Etd_2003_4	pin	cemetery	shaft		nm; partially oxidized	Cu-As
**233**	nd	Etd_2003_5	cup	cemetery	body	fragment	nm; partially oxidized	Cu-Sn
**234**	nd	Etd_2003_6	pin	cemetery	shaft		nm; partially oxidized	Cu-Sn
**235**	nd	Etd_2003_7	pin	cemetery	shaft		nm; partially oxidized	Cu-Sn
**236**	nd	Etd_2003_8	pin	cemetery	shaft		nm; partially oxidized	Cu-Sn
**237**	Ro.06/M124	Etd_21	ring	cemetery	body		4.1	Cu-Sn
**238**	Ro.06/M143	Etd_23	pin	cemetery	shaft		14.25	Cu-Sn
**239**	nd	Etd_24	pin	cemetery	head		38.5	Cu-Sn
**240**	Ro.06/M143	Etd_25	pin	cemetery	head		16.0	Cu-Sn-Sb
**241**	Ro.06/M144	Etd_26	pin	cemetery	head		5.4	Cu-As
**242**	nd	Etd_27	bead	cemetery		fragment	nm	Cu-As-Ag-Au
**243**	Ro.06/M145	Etd_28	pin	cemetery	head		7.9	Cu-Sn
**244**	Ro.06/M145	Etd_29	pin	cemetery	head		21.6	Cu-Sn
**245**	Ro.06/M120	Etd_3	pin	cemetery	head		5.5	Cu-Sn
**246**	Ro.06/M147	Etd_31	pin	cemetery	shaft		9.0	Cu-Sn
**247**	Ro.06/M147	Etd_32	cup	cemetery	body	fragment	52.75	Cu-As
**248**	nd	Etd_35	bead	cemetery	body	tubular	not measured	Cu-Sn
**249**	Ro.06/M154	Etd_37	axe	cemetery	blade		101.8	Cu-Sn
**250**	Ro.06/M126	Etd_4	pin	cemetery	shaft	vase-headed	18.3	Cu-Sn
**251**	M126	Etd_41	cup	cemetery	body	fragment	17.85	Cu-As
**252**	Ro.06/M126	Etd_5	pin	cemetery	head	vase-headed	16.7	Cu
**253**	Ro.06/M121	Etd_6	pin	cemetery	shaft		16.75	Cu-As-Sn
**254**	Ro.06/M129, Etd_7	Etd_1041	pin	cemetery	shaft		14.25	Cu
**255**	Ro.06/M129, Etd_9	Etd_1043	pin	cemetery	shaft		3.4	Cu-As
**256**	Ro.04/Etd.2	Etd_980	hair ring	cemetery	body		2.15	Cu-Ag
**257**	Ro.04/Etd.3	Etd_981	pin	cemetery	shaft		11.15	Cu-Sn
**258**	Ro.04/Etd.4	Etd_982	pin	cemetery	shaft		2.45	Cu-As
**259**	Ro.04/Etd.5	Etd_983	ring	cemetery	body		19.1	Cu-Sn
**260**	Ro.04/Etd.6	Etd_984	axe	cemetery	blade		34.63	Cu-As
**261**	Ro.04/Etd.7	Etd_985	pin	cemetery	shaft		7.3	Cu-Sn
**262**	Ro.04/Etd.8	Etd_986	pin	cemetery	head, shaft		11.6	Cu-As
**263**	Ro.04/Etd.10	Etd_988	pin	cemetery	shaft		2.45	Cu-Sn
**264**	Ro.04/Etd.11	Etd_989	pin	cemetery	head, shaft		20.45	Cu-Sn
**265**	Ro.04/Etd.12	Etd_990	earring	cemetery	gold leaf coating	lead core coated with Au	1.75	Cu-Au
**266**	Ro.04/Etd.13	Etd_991	pin	cemetery	shaft		1.65	Cu-As-Sn
**267**	Ro.04/Etd.14	Etd_992	cup	cemetery	body		29.25	Cu-Sn
**268**	Ro.04/Etd.15	Etd_993	pin	cemetery	shaft		49.3	Cu-Sn
**269**	Ro.04/Etd.16	Etd_994	bracelet	cemetery	body		20.5	Cu-Sn
**270**	Ro.04/Etd.17	Etd_995	pin	cemetery	shaft		25.03	Cu-Sn
**271**	Ro.04/Etd.18	Etd_996	pin	cemetery	shaft		22.8	Cu-Sn
**272**	Ro.04/Etd.20	Etd_998	torque	cemetery	body		32.8	Cu-Sn
**273**	Ro.04/Etd.21	Etd_999	cup	cemetery	body		69.0	Cu-Sn
**274**	M28	nd	cup	cemetery	body	lead cup	>300.0	Pb
**275**	Mo.04_M70	nd	ring	cemetery	body		nm	Cu
**276**	Mo.04_M80	nd	ring	cemetery	body		2.1	Cu-Sn
**277**	Mo.04_M82	nd	bead	cemetery	body	disc	0.55	Cu-Ag
**278**	Ro06_19_M140	nd	bracelet	cemetery	body		49.65	Cu-As
**279**	Ro.11_8	9104	pin	cemetery	head		nm	Cu
**280**	Ro.11_8	9104	pin	cemetery	shaft		nm	Cu-Sb
**281**	Ro.11_10	9106	pin	cemetery	head		nm	Cu
**282**	Ro.11_10	9106	pin	cemetery	shaft		nm	Cu-As
**283**	nd	Etd_1243	pin	cemetery	head		nm	Cu-Sb
**284**	nd	Etd_1243	pin	cemetery	shaft		nm	Cu-As
**285**	nd	Etd_1244	pin	cemetery	head, shaft		nm	Cu-Sb
**286**	Ro.15_22	9651	axe	settlement	blade		nm	Cu-As
**287**	Ro.10_6	Etd_1248	pin	cemetery	head		nm	Cu-Sb
**288**	Ro.10_6	Etd_1248	pin	cemetery	shaft		nm	Cu
**289**	Ro.10_1	Etd_1260	pin	cemetery	head		nm	Cu-As
**290**	Ro.10_1	Etd_1260	pin	cemetery	shaft		nm	Cu-As-Sb
**291**	Ro.10_2	Etd_1261	pin	cemetery	head, shaft		nm	Cu-As
**292**	Ro.11_8	Etd_1276	pin	settlement	head, shaft		nm	Cu-Sb
**293**	Ro.11_22	Etd_1290	pin	settlement	head, shaft		nm	Cu
**294**	Ro.11_28	Etd_1303	pin	settlement	head, shaft		nm	Cu
**295**	Ro.11_29	Etd_1304	pin	settlement	head, shaft		nm	Cu-As
**296**	Ro.11_30	Etd_1305_1	earring	cemetery	head		nm	Cu
**297**	Ro.11_30	Etd_1305_1	earring	cemetery	shaft		nm	Cu-Sb
**298**	Ro.11_30	Etd_1305_2	earring	cemetery	head		nm	Cu
**299**	Ro.11_30	Etd_1305_2	earring	cemetery	shaft		nm	Cu-Sn-Sb
**300**	Ro.11_31	Etd_1306_1	bead	cemetery	body		nm	Cu
**301**	Ro.11_31	Etd_1306_2	bead	cemetery	body		nm	Cu
**302**	Ro.11_31	Etd_1306_3	pendant	cemetery	body		nm	Cu
**303**	Ro.11_31	Etd_1306_4	pendant	cemetery	body		nm	Cu-Sn
**304**	Ro.12/7	Etd_1320	drill?	settlement	body	with wooden handle	nm	Cu-As
**305**	Ro.12/71	Etd_1356	drill?	settlement	body		nm	Cu
**306**	Ro.12/72	Etd_1357	chisel	settlement	body		nm	Cu-As
**307**	Ro.12/81	Etd_1362	pin	settlement	head, shaft		nm	Cu

## Analytical methodology

### pXRF analysis

Over the last decade, the use of pXRF has become increasingly common in the field of archaeology for the study of various types of artifacts and materials, as this analysis is effective in terms of both time and cost. This method is beneficial for museum-housed objects in countries like Turkey where permits are restricted and controlled, and tight regulations exist regarding the movement of artifacts. The analyses of the Resuloğlu artifacts were conducted between 2016 and 2018 in the Çorum Museum. The rules and regulations of the Turkish Ministry of Culture and Tourism were followed precisely during museum studies. This included but was not limited to a restriction on sampling from complete pieces or conducting surface cleaning on certain objects, especially on precious metals like gold.

While pXRF is an effective way for non-invasive analyses of museum artifacts, it has undeniable handicaps [e.g., 50–52]. It is a surface-analysis technique and can be used to analyze only to a depth of approximately 0.05mm, depending on the target element and matrix of the artifact. The technique cannot, therefore, provide bulk compositions for the objects. Confirmation of the bulk analysis or metallurgy of the artifacts through destructive methods or metallography was not possible, due to limitations in the allocation of museum-based research permits. The surface analysis might also read elevated values of tin due to surface segregation. To minimize this problem, all surfaces were mechanically cleaned in the Çorum Museum.

A total of 307 objects with appropriate surface conditions were analyzed by using a portable Bruker Tracer SD-IV pXRF spectrometer. Analyses were conducted on a stand to minimize any instability caused by the vibration of the instrument. Out of the 400 metals and metal fragments, only 307 were analyzed. Almost 25% of the complete collection was not analyzed due to severe corrosion.

The X-ray tube type used was a rhodium target. 40kV and 15.80μA were selected under air (non-vacuum), with a titanium-aluminum filter of 25μ titanium in layer 1 and 300μ aluminum in layer 2. This is the automatic filter specification of the instrument in filter number one and is the standard setting of the instrument used for metal analysis. The instrument was calibrated according to copper alloy standards BCR 691 A, C, D, and BAM 211. The analysis time was adjusted to 180 seconds. Each reading was repeated three times to obtain an average. The majority of the artifacts were analyzed at least from two different spots.

Chemical compositions are treated as decisive for alloy types. Elemental composition of greater than 1 wt% is accepted as an alloy (bronze, arsenical copper, etc.). Lead is an exception for which the limit is set as 5 wt%. These limits are used here are in concordance with similar studies [e.g., 5, 6, 53], hence allowing us to present a common ground for comparisons. All percentages referred here are given wt%. The average of the compositional results with analysis points is provided in S1 Table. This methodology follows similar studies from the region [6, 53].

### Lead Isotope Analysis (LIA)

The use of lead isotope analysis allows researchers to discover from which ore sources the copper in an artifact has been derived [54, 55]. This helps to reconstruct certain trade and exchange networks, thus indicating socio-economic linkages. In this study, 42 samples were taken from 40 different specimens. Samples with compositional results were prioritized for isotopic analysis. However, 12 samples lack pXRF results due to the level of surface corrosion.

**Fig 7:**
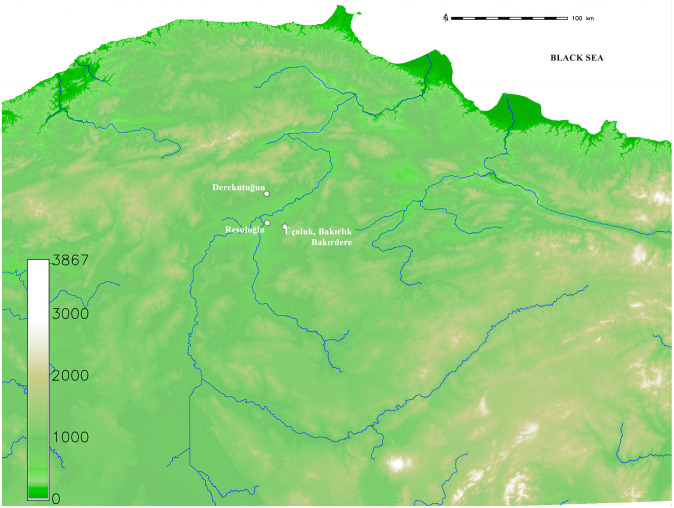
Map showing the research area of the Delice Valley Survey (image courtesy of Bülent Arıkan).

The lead isotope ratios measured on the Resuloğlu metals were then compared with the extensive database of ores compiled from the literature [e.g., 56–62]. The data was then correlated with samples from the Delice Valley Survey (hereafter DVS) project conducted in the region between 2016–2018 by a team of archaeologists, geologists, and geomorphologists (Fig 7). The DVS focused specifically on ore sampling at the eastern zone of the Delive River for two reasons: 1) the proximity of these sources to Resuloğlu, and 2) to complement the isotopic data already available for the northern Anatolian ore deposits [56]. Seeliger et al.’s [56] research focusing on the north Anatolian ores together with excavations at Derekutuğun cover the western part of the river where the Derekutuğun copper deposits are located [62].

Yalçın and İpek [62] have argued Derekutuğun as the ultimate copper source for the 3^rd^ millennium BC settlements in north-central Anatolia and beyond. Their technological deterministic approach is based on the fact that early societies were using Derekutuğun due to its massive capacity, easy availability, and closeness to ancient settlements. While there is no doubt about the rich reserves of Derekutuğun, their argument does not discuss cultural choices and priorities, thus is inadequate to explain the socio-economic systems of past societies. Consequently, the DVS prioritized the understudied small sources around the region to expand the data and to test the hypothesis of whether Derekutuğun has been the source of copper to all Resuloğlu copper-based metals. Accordingly, five native copper and malachite samples from Killik Tepe, Öksen Deresi, Bakırçay, and Üçoluk Deresi at the Karaevliya village were sampled to expand the available lead isotope dataset (S2 and S3 Figs).

Lead isotope ratio analyses were carried out at the Middle East Technical University Radiogenic Isotope Laboratory using a Thermo-Fischer Triton TI TIMS in static multi-collection mode. The procedure, published in Batmaz et al. [63: p. 408–411], was followed with slight changes. All weighing, chemical dissolution, and chromatographic procedures were carried out using ultrapure water and chemicals in the Class 100 cleanroom. Each sample weighed approximately 100 mg and was transferred to high-purity PFA (perfluoroalkoxy copolymer resin) vials. The samples were completely dissolved in 4 ml 14 HNO_3_ on a 160°C hotplate for two days. After being dried on a hotplate, the samples were dissolved in 4 ml of 6N HNO_3_ for one day. They were then evaporated on a hotplate before being dissolved again in 1 ml 2N HCl.

The lead was separated from the dissolved archaeological metal using Bio-Rad AG1-X8, 100–200 mesh anion-exchange resin in 1 ml columns. A total of 2 M HCl and 0.8 M HBr were used during the chromatographic filtration, and then lead was collected using 6M HCl. The sample was loaded on re-filaments using silica gel and 0.005 N H_3_PO_4_, and measured in static mode between 1200–1350°C. Analytical uncertainties were given at 2-sigma. The measurement accuracy was controlled with frequent measurements of the NIST SRM 981 lead wire standard reference material alternating with the course of the sample measurements. The NIST SRM 981 standard was measured as 16.938, 15.493, and 36.708 (n = 10) for ^206^Pb/^204^Pb, ^207^Pb/^204^ Pb, and ^208^Pb/^204^Pb respectively. By using NBS values, necessary corrections were made. The analytical accuracy was consistently around or less than 0.1%, which is sufficient for the determination of provenance [61, 64]. The ^206^Pb/^204^Pb, ^207^Pb/^204^Pb, ^208^Pb/^204^Pb, ^207^Pb/^206^Pb, ^208^Pb/^206^Pb, and ^208^Pb/^207^Pb ratios and standard errors for Resuloğlu metal samples and five new ore deposits are listed in S2 Table.

## Results

### pXRF analysis

pXRF analysis allows us to expose surface compositional analysis and to have a basic understanding of questions related to alloying choices. Nonetheless, in archaeometallurgical research, it is hard to draw a line between intentional and unintentional alloying. Relevant literature presents variable distinctions between the deliberate and accidental use of alloys [65: p. 114–115; 66: p. 62]. For this study, an elemental composition greater than 1 wt% is accepted as an alloy except for lead whose limit is set as 5 wt%. These limits used are in concordance with similar studies [6, 53], hence allowing us to present a common ground for comparisons. All the percentages presented in this study are weight percentages. The artifacts are referred to using the museum inventory numbers as stated in Table 2 and S1 Table.

**Fig 8:**
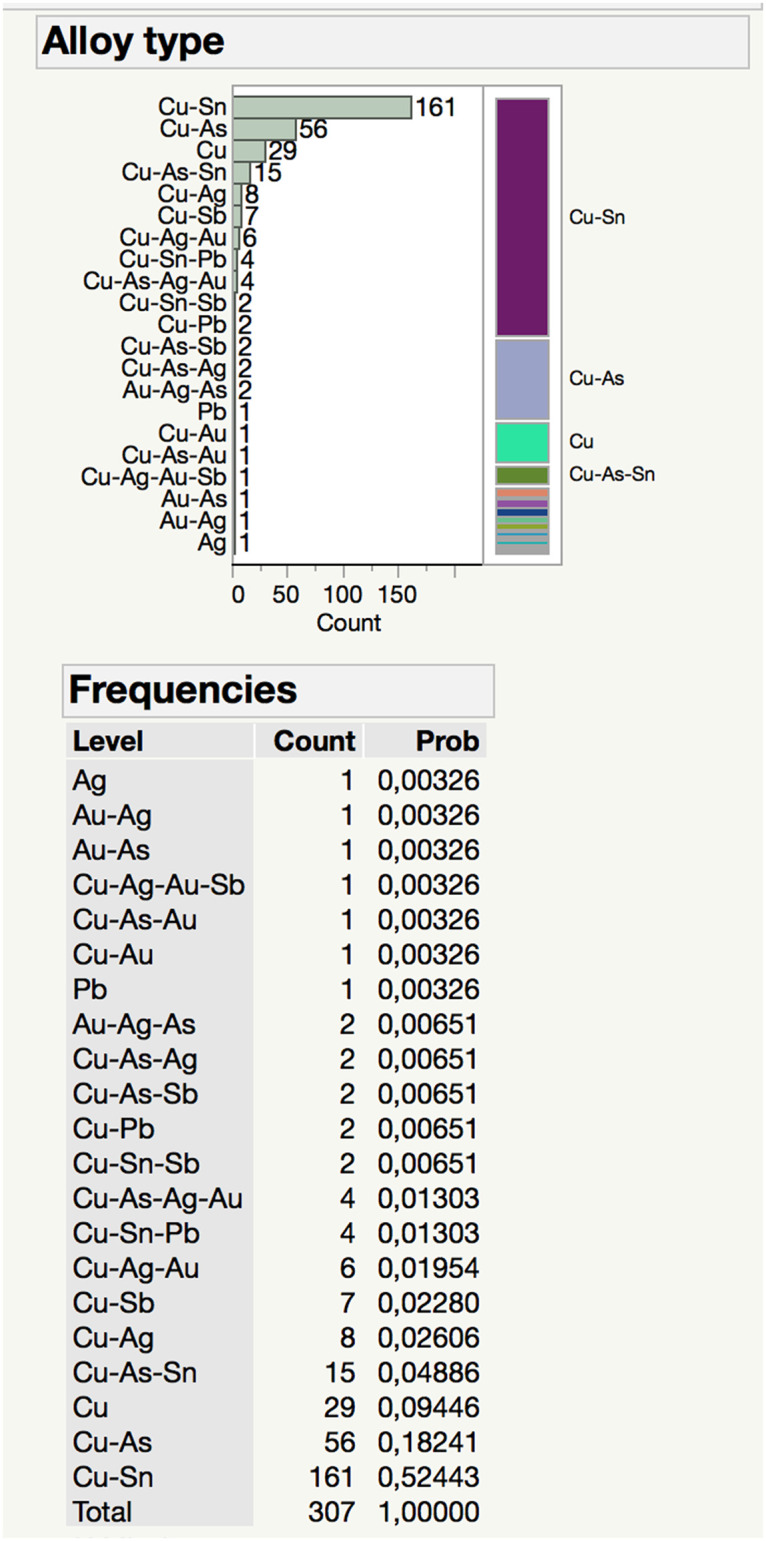
Distribution of metal and alloy types in the analyzed metal corpus of Resuloğlu.

The total of 307 objects from 18 different artifact types shows an unanticipated variety for the use of metals in the Resuloğlu assemblage. Eighteen different alloying practices along with the use of unalloyed copper, silver, and lead are identified (Fig 6). Bronze identified on 161 objects constitutes the most common alloy type. This is followed by arsenical copper alloy documented on 56 objects. Unalloyed copper is the third major group recognized in 29 artifacts. Unalloyed lead and silver are represented by one artifact each. The ternary alloy of Cu-As-Sn is detected on 15 pieces. The other alloying practices documented are Cu-Ag (8), Cu-Sb (7), Cu-Ag-Au (6), Cu-Sn-Pb (4), Cu-As-Ag-Au (4), Cu-Sn-Sb (2), Cu-As-Ag (2), Au-Ag-As (2), Cu-Au (1), Cu-As-Au (1), Cu-Ag-Au-Sb (1), Au-As (1), and Au-Ag (1) (Fig 8).

**Fig 9:**
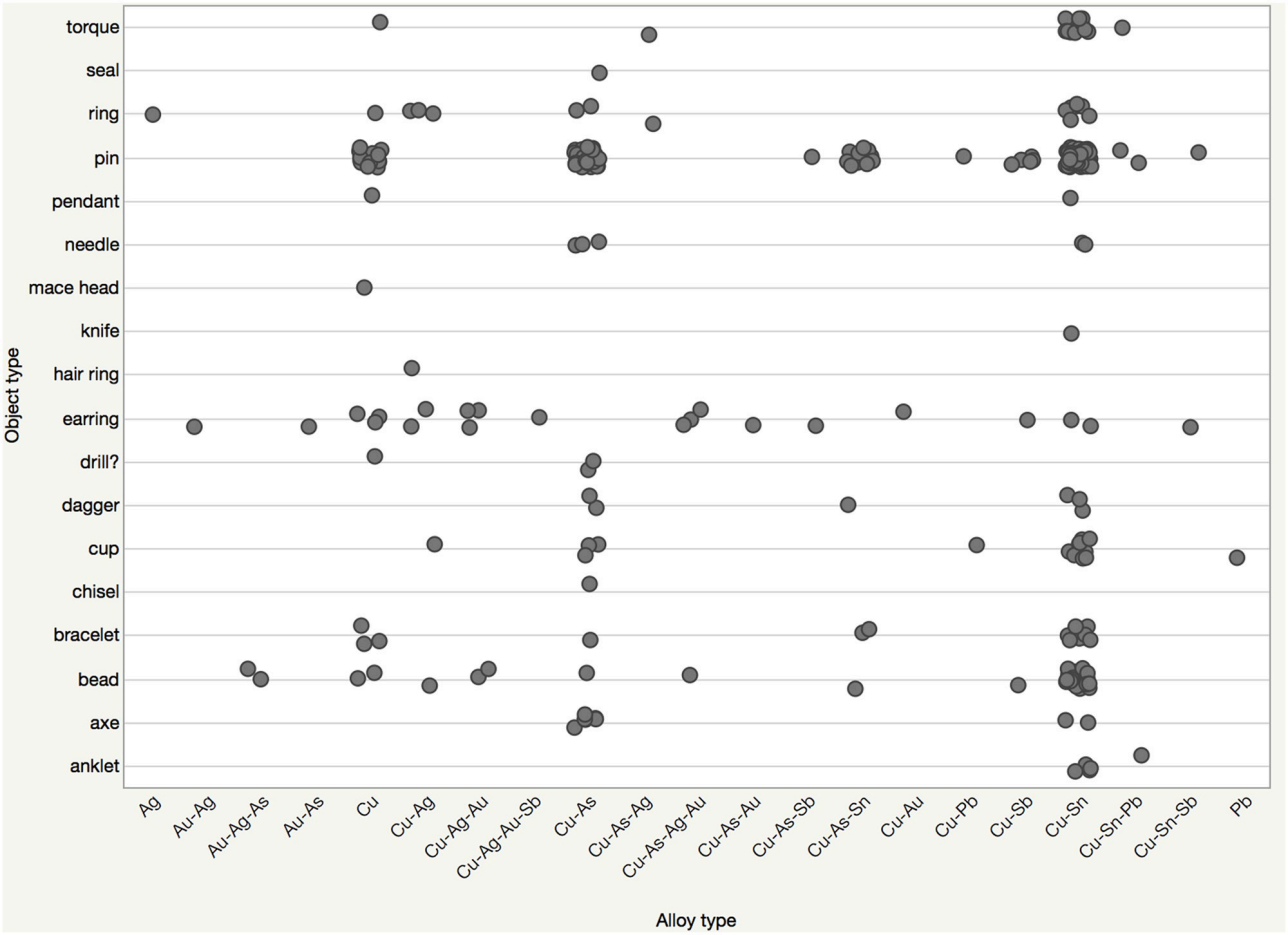
Object type versus alloy type among the Resuloğlu metal corpus.

A statistically reliable relation is not confirmed between object type and alloy type (Fig 9). Still, certain artifact types favor particular alloys. For example, 28 out of 40 of the beads are made of bronze. Similarly, pins, anklets, torques, and bracelets also favor bronze, but examples made of other alloys were identified. Anklets are made of either bronze (4) or Cu-Sn-Pb (1) alloys. While the majority of the bracelets are made of bronze, unalloyed copper (3), arsenical copper (1), and Cu-As-Sn (2) examples were also identified. The only chisel (Etd_1357) of the assemblage is arsenical copper containing 97.57% copper and 1.5% arsenic. The only metal seal was found in the settlement and is arsenical copper with 9.25% arsenic.

Sixteen cups examined in the assemblage display five different compositions: Cu-Ag (1), Cu-Pb (1), arsenical copper (3), bronze (10), and lead (1). The Cu-Ag cup (7902) weighs 42 gr and consists of 65.7% copper and 25.37% silver. The Cu-Pb cup fragment (Etd_1006) contains 93.6% copper and 5.65% lead and weighs 17.85 gr. The only unalloyed lead artifact is also a cup from a burial context (M28) containing 94.7% lead. Drills are made of unalloyed copper (1) and arsenical copper (2). Needles are either made of copper alloyed with arsenic (3) or tin (2).

Among the implements, six of the eight axes are arsenical copper. The remaining two are bronze. Daggers were manufactured from bronze (3), arsenical copper (2), and Cu-As-Sn (1). The only knife (Etd_1140) analyzed in the assemblage is bronze with 9.39% tin. The only mace head found at Resuloğlu (7901) weighs 178.95 gr and contains 98.94% copper with trace amounts of arsenic and antimony.

**Fig 10:**
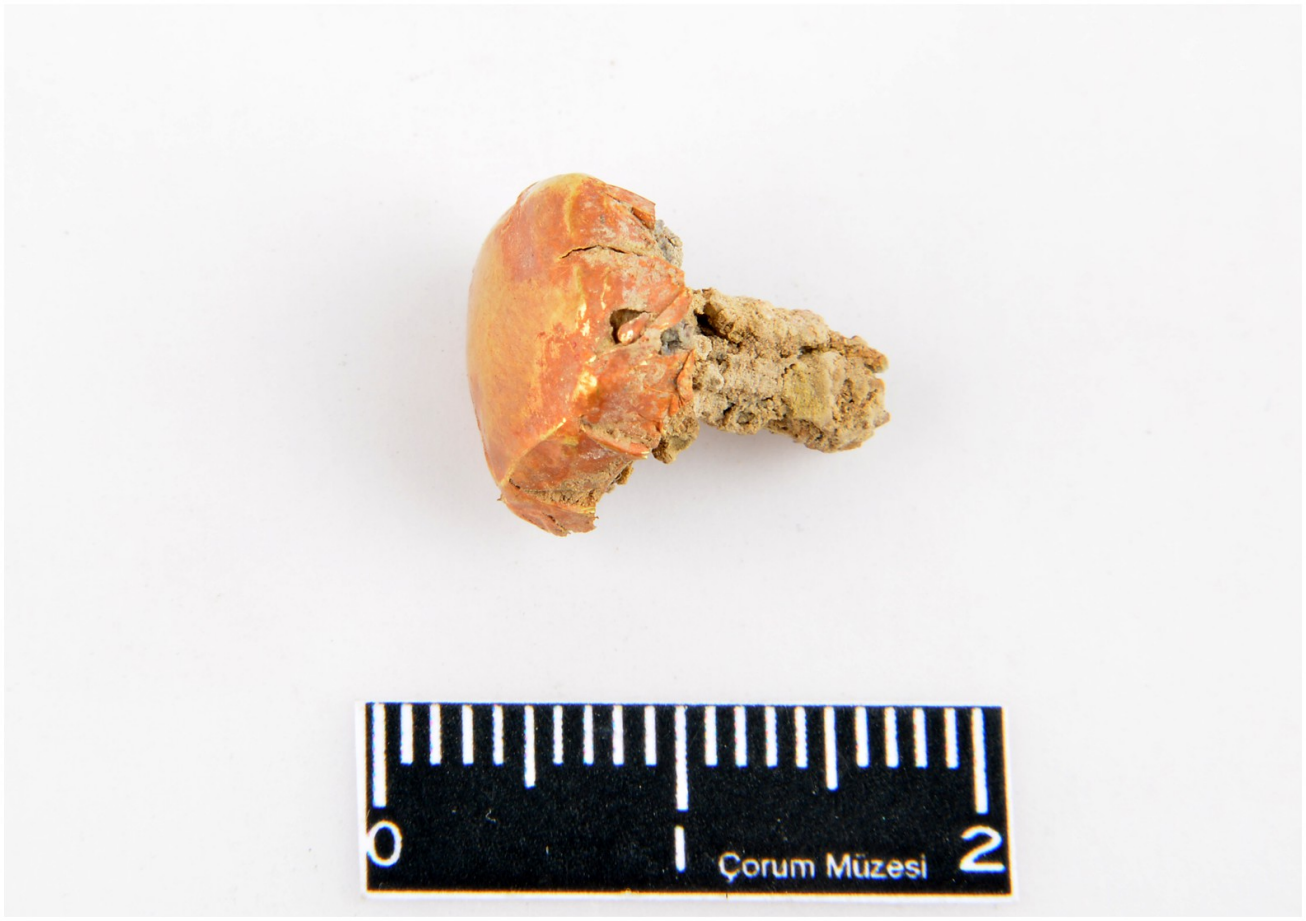
Lead-cored, copper-gold (Cu-Au) leaflet-coated earring with the inventory number Etd 990 (©Resuloğlu excavations archive).

Various alloys include gold but no pure gold objects were detected. An earring (8625) made of stone is coated with a thin leaf of Au-Ag alloy containing 95.36% gold and 3.47% silver. A lead earring (Etd_990) is coated similarly with a golden-colored leaf, which is determined as a Cu-Au alloy of 3.91% copper and 85.92% gold. The 2.52% lead detected on the object must be due to the core (Fig 10).

One earring (Etd_1194) contains 2.09% arsenic and 94.23% gold. The ternary alloy of Au-Ag-As is determined in two beads (7908_1, 7908_3). These swastika-shaped beads contain similar percentages of gold (93.23% and 93.35%), silver (1.47% and 1.4%), and arsenic (1.25% and 1.46%) (S3 Fig). Cu-Ag-Au and Cu-As-Ag-Au alloys are identified on beads and earrings. An earring (8614_1) containing 27.3% copper, 51.96% silver, 16.6% gold, and 4.22% antimony makes the object unique not only for Resuloğlu but also for the regional contemporaneous assemblages [6].

The only silver object is a ring (Etd_1151_1) containing 41.51% silver. The total weight percentage of the object is below one hundred due to chlorine ions of the silver oxidation, which cannot be measured via pXRF. The only hair ring (Etd_980) in the collection is made of Cu-Ag with 61.68% copper and 21.61% silver. Cu-As-Ag alloy is detected on a ring (Etd_1083) and a torque (7701).

Antimonial copper objects, dated as early as the 4^th^ millennium BC, are known from the region [6]. In Resuloğlu antimony-bearing alloys include antimonial copper (bead, earring, pin), Cu-Sn-Sb (earring and pin), Cu-Ag-Au-Sb (earring), and Cu-As-Sb (earring and pin). Only one bead (Etd_1096_3) has been found as antimonial copper. This suggests a preference for antimony-bearing alloys for the production of pins and earrings.

The variety in the alloy types of the cemetery is not noticeable in the settlement. There are only 12 metal artifacts found in the settlement: a seal, a few pins, an axe (9651), a chisel (Etd_1357), and two drills (Etd_1320, Etd_1356). The most common alloy in domestic contexts is arsenical copper (axe, seal, chisel, and pins) followed by unalloyed copper. The only antimonial copper pin (Etd_1276) is from the settlement. The metal consumption at the settlement is not dense. The assemblage of the settlement mostly consists of utilitarian objects; the object types and alloys are not as diverse as in the cemetery. The only metal seal from the site was recovered at the settlement on the floor of a silo. No seals were found in the cemetery.

Bronze appears as the most common alloy in Resuloğlu. Bronze objects seem to be reserved for burials and not recovered from the domestic contexts. This demonstrates the intentional consumption of tin-bearing objects as burial goods. If tin-containing artifacts had been used at the settlement, they must have been removed and/or recycled during or after the settlement’s lifetime [e.g., 52].

While all tin-bearing alloys are accumulated at the cemetery, the settlement’s metal corpus relies on arsenical and unalloyed copper. The polymetallic sources containing copper, arsenic, and antimony were available within proximity to Resuloğlu (i.e., 10–100km) [6]. The availability and accessibility of minor deposits, most of which are unknown to modern scholars due to a lack of systematic surveys and data accusation, should have sufficed for small communities like Resuloğlu to exploit [67]. Besides, the patchy distribution of minor metallic deposits must have been difficult to control for any ruling group.

The 3^rd^ millennium BC north-central Anatolian sites offer a partial answer to whether the local settlers exploited local metal sources. There is not enough regional, well-stratified data to settle this. Nonetheless, it would be logical to argue that villages in the region periodically sent groups to mine and smelt ores for extensive seasonal mining [68: p. 137].

Tin is not locally available. The closest known tin sources to the region are in the Taurus Mountains and at Hisarcık [42–44]. This suggests that the reserved consumption of rare metals must have arrived at Resuloğlu via an exchange system. This argument applies also to gold and silver objects and their alloys, which have also been encountered only in the burial contexts.

The pXRF results demonstrate the difference in metal consumption between the settlement and the cemetery. The majority of the metals were left as burial gifts, whereas stone tools and implements appear commonly in use in the settlement. The low number of metal objects from the settlement supports this situation in the domestic areas. All of the metal objects at the settlement were found in regular houses, whereas the village-head’s residence did not yield any metals. This indicates that at Resuloğlu, the use of metal tools, the existence of bronze items, or the ownership of metals were not prime markers of the leading segments.

Metallographic examinations were not possible at Resuloğlu due to permit limitations. Thus, it is not plausible to contribute to metal production and working techniques. Additionally, no archaeological context relevant to metal production was uncovered at the site. Accordingly, our data validate that the settlers of the site had fairly good access to various types of metal items made of different alloy types. The lead isotope results support this suggestion.

### Lead isotope analysis

Lead isotope ratios are a frequently applied method to determine the possible ore sources of metal artifacts. The positive matches in lead isotope ratios indicate the source from which the metal could have come [69, with references]. Isotopic studies have been conducted to a lesser extent in Turkey due to the limitations of excavation and research budgets. Regarding this, the sample set presented in this study constitutes of a unique set to evaluate the flow of metals during the 3^rd^ millennium BC in north-central Anatolia.

The classification of metal goods as local or as an import has led the way to different scenarios for trade networks expanding from Mesopotamia to central Anatolia. For north-central Anatolia, the majority of scholars privilege local Hatti metalworking and metal schools [e.g., 24, 30, 47, 70]. According to such technologically deterministic models, the local Hattian craftspeople of north-central Anatolia were exploiting and processing the available sources such as the Pontides. These skilled local metalsmiths have been pointed out as the producers of the metal caches, yet the literature has a limited discussion of metallurgical production and activity zones. This is partially due to the continuous occupation of the region and the continuous exploitation of its metallic sources, as well as the high density of forestation near the Black Sea coast, which has hindered archaeological excavations and surveys.

The occurrence of mineral deposits in north-central Anatolia is far too numerous to even catalog here. While the metallic sources in the region are rich, it should not always be concluded that they were used as available sources. Behavioral and societal parameters such as choices of past communities might shape the use of proximate sources in a way that might not be driven purely by technology or function. Additionally, the archaeological evidence regarding the ultimate use of nearby sources is thin. However, metal assemblages of middle-range societies in north-central Anatolia have not been systematically sampled and analyzed to propose a new model that explains metal production and flow. Resuloğlu’s metal corpus is unique in that it provides the biggest lead isotope data from a single site.

**Fig 11:**
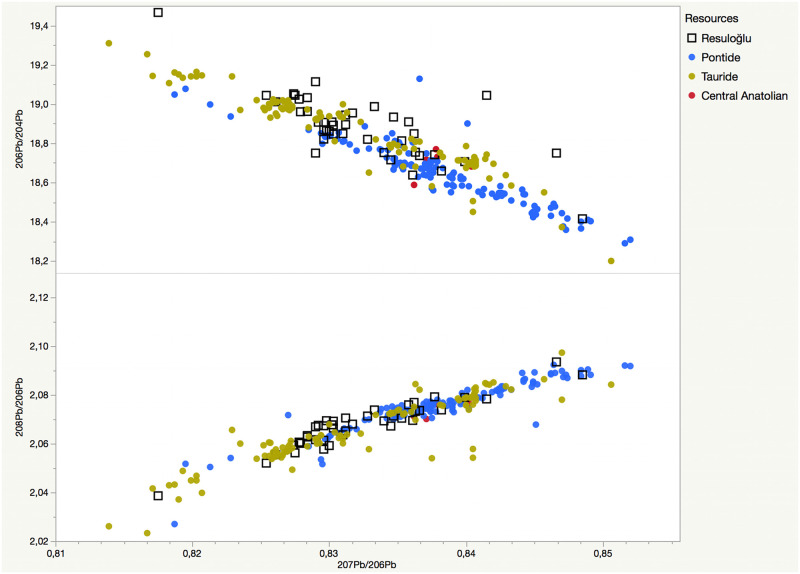
Lead isotope ratio binary graphs displaying ^207^Pb/^206^Pb vs. ^208^Pb/^206^Pb and ^206^Pb/^204^Pb of analyzed Resuloğlu metal samples compared to the published ore data from the Tauride and Pontide, and central Anatolia.

**Fig 12:**
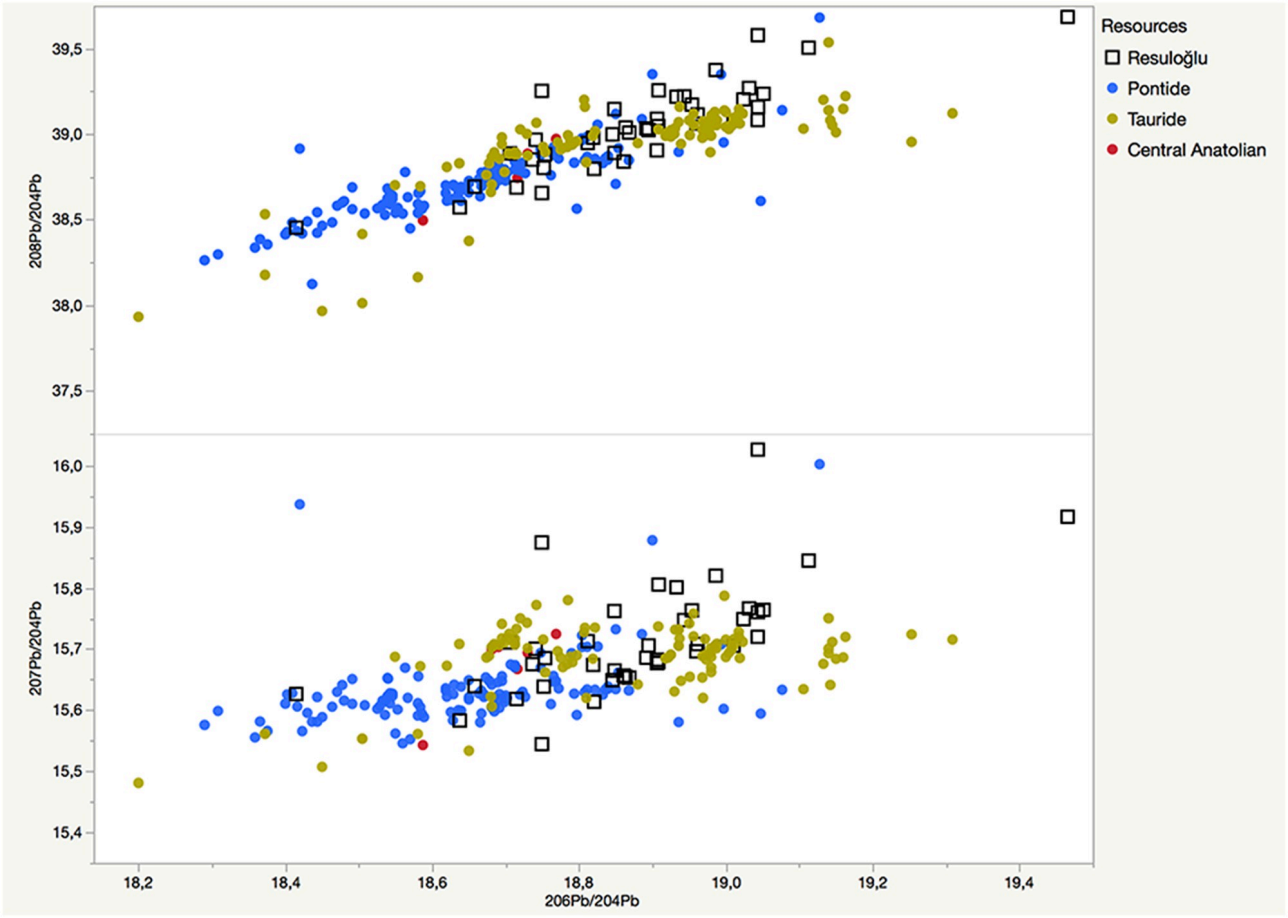
Lead isotope ratio binary graphs displaying ^206^Pb/^204^Pb vs. ^207^Pb/^204^Pb and ^208^Pb/^204^Pb of analyzed Resuloğlu metal samples compared to the published ore data from the Tauride and Pontide, and central Anatolia.

The lead isotope results were compared to data on available ore from the Caucasus, Iran, Cyprus, and Anatolia. Immediately apparent in the data set are the two separate groups of objects superimposed with the Anatolian ore sources. Thus, the Caucasus, Iran, and Cyprus data are eliminated from the following figures and discussion. Fig 11 presents ^207^Pb/^206^Pb versus ^208^Pb/^206^Pb and ^206^Pb/^204^Pb groupings for Resuloğlu metals compared with published ore data from the Tauride and Pontide, and central Anatolia. Fig 12 demonstrates ^206^Pb/^204^Pb versus ^207^Pb/^204^Pb and ^208^Pb/^204^Pb graphs. In both diagrams, the lead isotope ratios of Resuloğlu metals cluster at the Tauride and Pontide ores in almost equal numbers.

The lead isotope data is further grouped into six by using P. de Jesus’ model [24, 71], which broadly draws copper exploitation zones in prehistoric Anatolia based on certain artifact typologies and copper sources. De Jesus grouped the copper exploitation zones in prehistoric Anatolia into six plausible production zones: 1) Küre group refers to ores from Kastamonu, i.e, western Black Sea coast; 2) Yapraklı group includes sources from Ankara, Çankırı, Çorum, Amasya, and Yozgat area, i.e., central Black Sea and north-central Anatolian zone; 3) Pontic group involves ores of the Sivas and Tokat region; 4) Giresun-Trabzon group refers to the area spanning Ordu, Trabzon, and Gümüşhane; 5) Murgul-Kuvarshan group involves the western edge of the polymetallic sources of Erzurum, Kars, and Artvin. i.e., eastern Black Sea coast; and 6) Ergani group refers to the metallic ores of the Elazığ- Diyarbakir area. This zoning is particularly useful when site and resource distributions are concerned. Additionally, it presents a basic sense of resource accumulation. However, the borders of each zone should be considered hypothetical.

This study builds on this classification by also adding the Taurus Mountain range into the discussion. According to the lead isotope results, Bolkardağ-Aladağ-Niğde massif in the central Taurus range appears as a plausible provenance for a significant number of the samples. Table 3 lists the possible provenance of the metal objects from Resuloğlu as source groups and specifics of resource locations such as Ankara ores as part of the Yapraklı group or Bolkardağ-Aladağ as part of the central Taurus zone.

**Table 3:** Suggested provenance of the Resuloğlu metals based on the lead isotope analysis.

*Lab_code*	*Museum_No*	*Alloy type*	*Object Type*	*Source (Groups)*	*Specifics of the Source*
23913_01_Pb	M28	Pb	lead cup	Yapraklı	Ankara
23913_02_Pb	Etd 1016	nd	pin	Küre	Kastamonu
23913_03_Pb	Etd 14	nd	pin	Yapraklı	Amasya
23913_04_Pb	Etd 1109	nd	pin	Central Taurus	Aladağ
23913_05_Pb	Etd 1002	Cu-As	pin	Pontic	Sivas
23913_06_Pb	Etd 1112	Cu-Sn	dagger	Yapraklı	Ankara
23913_07_Pb	Etd 1100	Cu-Sn	cup	Central Taurus	Aladağ
23913_08_Pb	Etd 1143	Cu-Sn	cup	Central Taurus	Bolkardağ
23913_09_Pb	Etd 1237	Cu-Sn	cup	Central Taurus	Aladağ
23913_10_Pb	Etd 1195	Cu-As-Ag-Au	earring	Pontic	Sivas
23913_11_Pb	Etd 1206	Cu-Sn-Pb	torque	Central Taurus	Aladağ
23913_12_Pb	Etd 1238	Cu-Sn	pin	Central Taurus	Aladağ
23913_13_Pb	Etd 999	Cu-Sn	cup	Pontic	Yozgat
23913_14_Pb	M70	Cu	pin	Central Taurus	Aladağ
23913_15_Pb	Etd 982	Cu-As	pin	Central Taurus	Aladağ
23913_16_Pb	2003/11	Cu-Sn	cup	Central Taurus	Aladağ
23913_17_Pb	2003/4	Cu-As	earring	Yapraklı	Ankara
23913_18_Pb	2003/1	Cu-As	cup	Pontic	Sivas_Tokat, Öksen
23913_19_Pb	Etd 992	Cu-Sn	cup	Central Taurus	Bolkardağ
23913_19_Pb (2)	Etd 992	Cu-Sn	cup	Central Taurus	Bolkardağ
23913_20_Pb	Etd 1017	Cu-Sn	torque	Central Taurus	Bolkardağ
23913_20_Pb (2)	Etd 1017	Cu-Sn	torque	Central Taurus	Bolkardağ
23913_21_Pb	Etd 1031	Cu-As	dagger	Küre	Kastamonu
23913_22_Pb	Etd 1025	Cu-Sn	torque	Central Taurus	Bolkardağ
23913_23_Pb	Etd 39	Cu-As	pin	Yapraklı	Ankara
23913_24_Pb	Etd 998	Cu-Sn	torque	Pontic	Sivas
23913_25_Pb	2005/5	Cu-Sn	bead	Central Taurus	Bolkardağ
23913_26_Pb	2003/13, M30	nd	bead	Yapraklı	Ankara
23913_27_Pb	2003/13, M26	nd	bead	Yapraklı	Amasya
23913_28_Pb	Etd 38	nd	pin	Central Taurus	Aladağ
23913_29_Pb	Etd 1041	Cu	pin	Central Taurus	Bolkardağ
23913_30_Pb	2003/08, M9	Cu-Sn	pin	Yapraklı	Ankara (Üçoluk)
23913_31_Pb	2003/10, M28	Cu-As	pin	Yapraklı	Ankara (Üçoluk)
23913_32_Pb	2003/09, M26	nd	pin	Central Taurus	Bolkardağ
23913_33_Pb	Etd 1014	nd	pin	Yapraklı	Ankara/Amasya
23913_34_Pb	Etd 1024	nd	bead	Yapraklı	Üçoluk
23913_35_Pb	Etd 16	Cu-Sn	bead	Pontic	Sivas
23913_36_Pb	Etd 4	Cu-Sn	pin	Central Taurus	Aladağ
23913_37_Pb	Etd 37	Cu-Sn	axe	Central Taurus	Aladağ
23913_38_Pb	Etd 32	Cu-As	cup	Pontic	Sivas_Tokat, Öksen
23913_39_Pb	Etd 1011	Cu-Ag	ring	Central Taurus	Aladağ
23913_40_Pb	Etd 1022	Cu-Sn	cup	Central Taurus	Bolkardağ

The lead isotope analysis results display a diverse use and complex pattern of metal consumption. Accordingly, Resuloğlu has not only accessed metals from its proximate sources, but its trade network expanded towards the southern fringe of the Halys Basin as far as the Taurus Mountains. This presents for the first time a scenario contrary to the previously suggested models of metal production and consumption in north-central Anatolia.

The isotope ratios of Resuloğlu’s metals match the sources from both the north and the south. Out of 42, 22 samples cluster with the central Tauride ores, namely in the Bolkardağ and Aladağ regions. The remaining 20 samples relate to the Yapraklı, Pontic, and Küre groups.

Among the northern sources, 11 samples relate to the Yapraklı group, specifically Ankara, Amasya, and Çorum Üçoluk. Seven metal samples demonstrate Pontic provenance, mostly to Sivas. The Küre group (Kastamonu) is the possible provenance of a pin (Etd_1016) and a dagger (Etd_1031). Giresun-Trabzon, Murgul-Kuvarshan, or Ergani groups are not likely provenances for any objects. This signals that Resuloğlu’s exchange and trade network did not expand to the eastern part of the Black Sea coast.

The isotopically analyzed metals constitute less than a quarter of the whole metal collection. Nonetheless, the results disprove the suggestion that the 3^rd^ millennium BC communities of north-central Anatolia have been benefiting solely from local or proximate sources. Resuloğlu displays an almost equal distribution of metals sourced from both northern and southern sources. Especially for metals provenanced to the central Taurus, a direct or an indirect supply chain must have existed. Resuloğlu’s chief might have controlled to a degree the supply and accumulation of metals. The settlers of the site must have been part of this supply chain by providing grain and possibly textiles.

**Fig 13:**
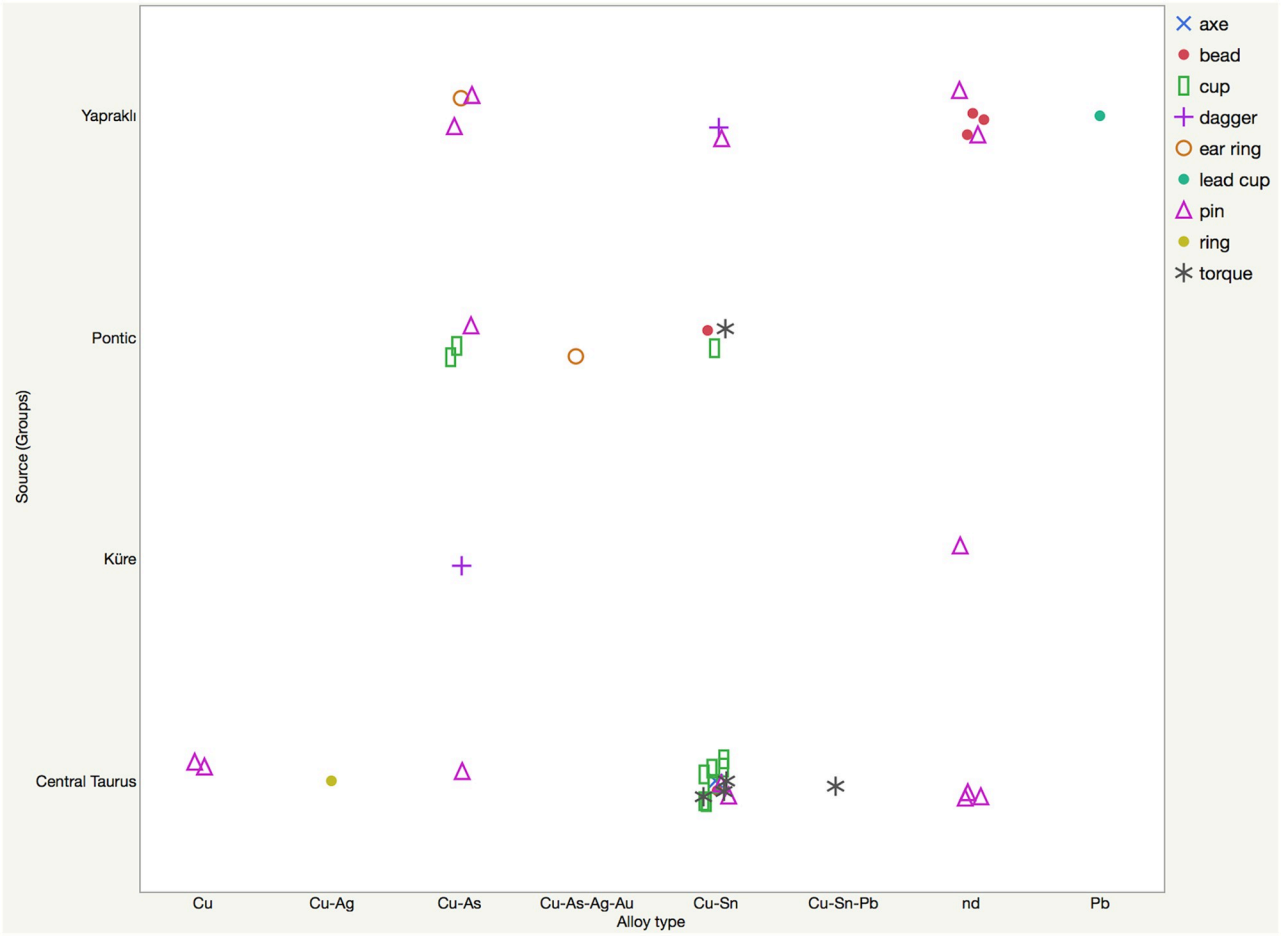
Graphical representation of alloy type versus provenance; object types are provided in the legend.

**Fig 14:**
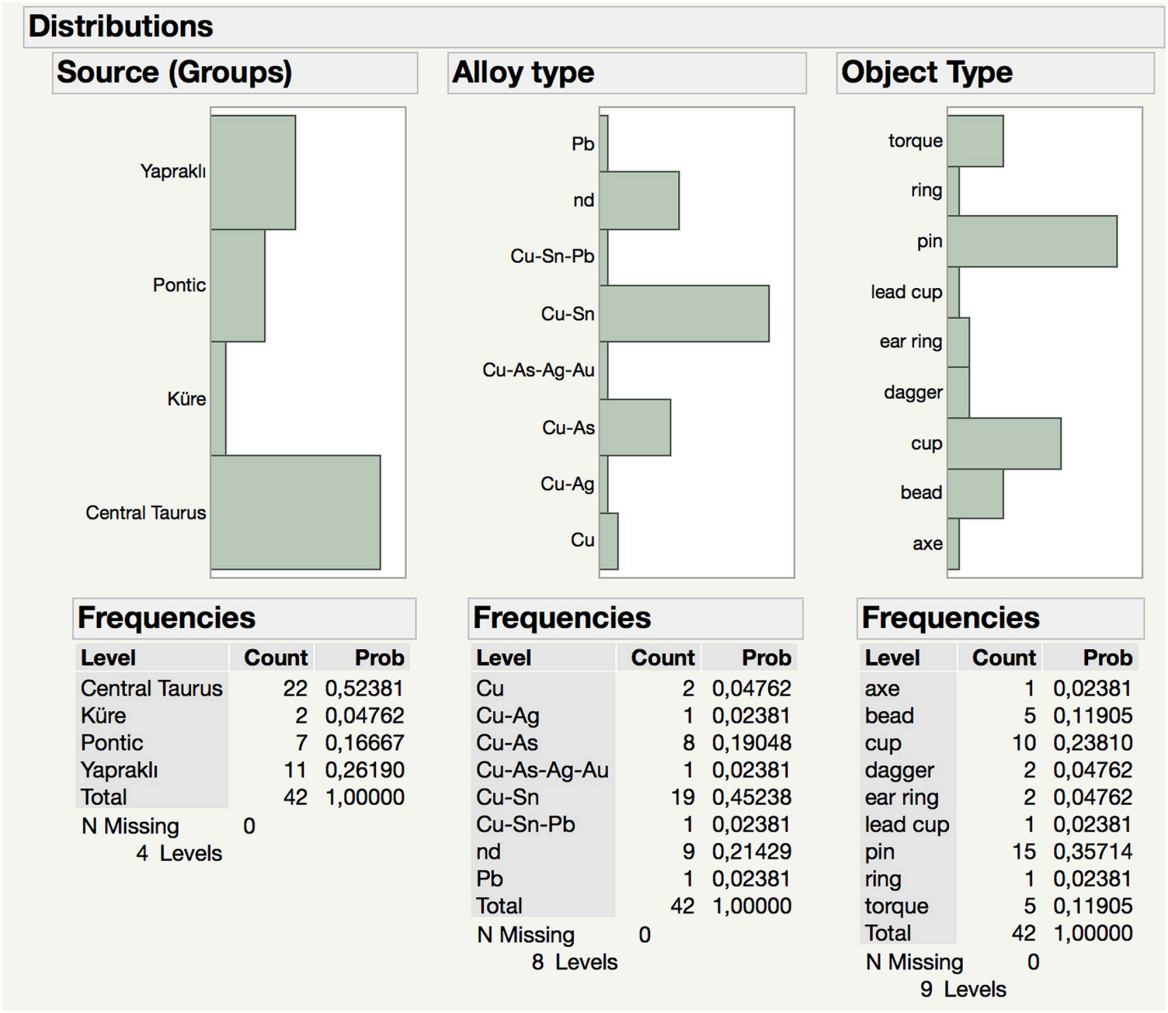
Numerical distribution graphs of alloy types and object types among suggested provenance (source groups) of metal objects.

Suggesting a definite relation between alloy type, object type, and provenance is not easy at Resuloğlu (Figs 13 and 14). For example, the provenances of different arsenical copper objects match those of Yapraklı, Pontic, Küre, and the central Taurus. Two unalloyed pins (M70, Etd_1041) and a Cu-Ag ring (Etd_1011) point to the central Taurus as a possible provenance. A Cu-As-Ag-Au earring (Etd_1195) matches the Pontic group (Sivas). Thus, a comparison at this stage is not possible. The only unalloyed lead object of the Resuloğlu collection, cup M28, falls into the Yapraklı group. The lead isotope ratios of this lead cup match well with the jamesonite (Pb_4_FeSb_6_S_14_) sample from Kızılcahamam, Ankara.

Bronze objects show Yapraklı (2), Pontic (3), and the central Taurus (14) as likely provenances. The number of bronze finds originating from the Bolkardağ and Aladağ zones constitutes approximately 73.4% of the isotopically analyzed samples (Fig 14). While the existence of tin in the Yapraklı and Pontic regions is unclear, tin is present at the Niğde massif (Bolkardağ) of the central Taurus range [72]. For the exploitation of tin during the 3^rd^ millennium BC at the Niğde massif, a multi-tier production model composed of a mining site (Kestel) and its associated specialized mining settlement (Göltepe) was suggested [42, 43, 73].

Lead isotope ratios for the majority of bronze objects from Resuloğlu coincide with the isotopic ratios of Bolkardağ and Aladağ. This supports an operating production zone during the 3^rd^ millennium BC in the central Taurus, for which Kestel-Göltepe appears the most plausible candidate. Some typological similarities between the metals of Resuloğlu and Göltepe are intriguing to note. For example, the majority of the bronze torques from Resuloğlu is provenanced to the central Taurus, where a typological counterpart made out of silver was documented at Göltepe [42: pl.27, M23; 43: p. 206, Fig 23]. The provenance of a Cu-Sn-Pb torque (Etd_1206) from Resuloğlu also falls into the central Taurus group, specifically in the Aladağ zone.

The central Tauride isotopic signatures match well with most of the torques and cups among the Resuloğlu metals. Similar objects with a Pontic ore signature are also available at Resuloğlu. The beads are clustered with Ankara, Amasya, and Üçoluk sources. One bronze bead (Etd_16) falls into Pontic-Sivas cluster while the other (2005/5) aligns well with Bolkardağ ratios. The isotope ratios of the pins align with all four groups.

There are eight axes in the Resuloğlu metal collection, only one of which could be sampled. The lead isotope ratios of this bronze axe (Etd_37) correspond to Aladağ. One of the arsenical copper daggers (Etd 1031) originates from Küre-Kastamonu, whereas the copper of the bronze example (Etd 1112) is more likely to come from the Yapraklı ores, specifically from Ankara. The typologies of both daggers were already identified with the northern styles [30, 74]. This archaeological suggestion is now confirmed with analytical data.

To sum up, any relationship between the object type and the potential source does not exist. For example, there is no evidence to confidently argue that all torques from Resuloğlu originated from the central Taurus or that all pins are from Küre or Yapraklı. This shows that some potential resource areas could not be equated with specialized workshops to produce certain types of metal items. There is decentralization in production and consumption.

## Discussion

The compositional results of the Resuloğlu metals demonstrate significant diversity in metal and alloy consumption in the late 3^rd^ millennium BC north-central Anatolia. While pXRF does not provide information about the bulk compositions of the artifacts, the method successfully presents variety in metal composition. Various binary and ternary alloys of copper were identified among which bronze is the most common. Alloys of copper with silver and gold were revealed. It is hard to draw a line between intentional and unintentional alloying at Resuloğlu, but natural alloys might be deliberately used [cf. 6, 75].

The 3^rd^ millennium BC Anatolian metallurgy has been so far oversimplified to unalloyed copper, arsenical copper, and bronze through interpreting the evidence via normative modes of consumption. This appears as overgeneralizing the data from small, subjectively selected metals, failing to notice diversity. The compositional results of a broad corpus from a single site present such an assortment for the first time.

The lead isotope ratios provide detailed implications of metal transactions at Resuloğlu. The results define a flow of metals to the site from two major locales: 1) relatively proximate sources such as Yapraklı, Küre, and Pontic, among which Yapraklı is the closest network, and 2) macro-regional exchange outside the Halys Basin, i.e., the central Taurus region. This macro-regional metal flow exposes that the 3^rd^ millennium BC societies of north-central Anatolia have indeed imported metals despite being in proximity to deposits. These metals were possibly exchanged in finished forms because any semi-finished fragment or assemblage associated with metalworking such as molds, tuyeres, crucibles, or slags has not been uncovered at the site or in its vicinity. The metal artifacts uncovered in both domestic and burial contexts confirm that the settlers of Resuloğlu were consumers of metal products rather than being producers of them.

The consumer side of the metal flow has been mostly overlooked in Anatolian archaeology [cf. 76: p. 145; 77: 88–89]. Among the metals at Resuloğlu, the most common items are overwhelmingly personal ornaments such as pins and beads; tools are rare. Any regalia like those found in contemporary Alaca Höyük, Horoztepe, Eskiyapar, or Mahmatlar are absent. This signals that the primary consumer demand at Resuloğlu was personal ornamentation, and secondarily for implements. There is no noticeable demand for items related to political or religious ceremonies [cf. 2: p.124].

At Resuloğlu, the archaeological and analytical data demonstrate an excellent case for metal consumption in a middle-range society, where evidence about the existence of elites is absent. Thus, Resuloğlu’s metal artifacts are better identified with valuables [78] than prestige goods [1].

The accumulation of wealth in terms of metals in burial contexts is not a marker of social inequality [79: p.250]. At Resuloğlu, differential wealth is notable with diverse numbers of burial gifts including ceramics, semi-precious stones, faience, or shell; however, a large number of metal objects was not found in a single grave. While this study did not cover the full context of the burials due to a forthcoming publication focusing on the cemetery, 20 years of excavations and studies demonstrate that the archaeological and anthropological data will hardly render a visibly and tangibly stratified community.

According to Hayden [80], wealth accumulation in subsistence economies favors more useful, storable food stocks rather than rare goods. This argument resonates at Resuloğlu, where grain silos had served as food supplies. Valuables like metals, faience, or carnelian have a role in a non-domestic context. Compared to the other 3^rd^ millennium BC sites in the region where social inequality is more apparent from burial goods and practices like Alaca Höyük, we argue that social inequality resonates in different pathways within the same region. There have to be changing relationships of social position, wealth, control, and ritual roles within north-central Anatolian communities [cf. 79: p.234]. On a regional scale, different communities must have been interacting with each other for economic advantage [2].

Resuloğlu did not yield any remains related to metal production. Thus, any discussions at this stage about the context of production based on metals found in a burial context will be misleading. Focusing on the nonstate exchange systems is promising to explore the consumer side of metal economics at Resuloğlu. The diversity in metal compositions and types at Resuloğlu indicates metal acquisition without elite control in the Halys Basin. Nonelite-controlled metal trade is not unique to Anatolia but also documented in various parts of the world like China, Thailand, Spain, and among Asian pastoralist groups [81].

Nonstate economies generally have a variety of exchange and trade systems due to their incorporation into nonmarket economies. In less stratified, middle-range societies like Resuloğlu, reciprocal exchange is one of the pillars of the economic system [82]. At Resuloğlu, a give-and-take system established in alliance networks would have worked not only for valuables but also for basic stuff like ceramics or textiles. The ceramic corpus of the site is local to the Halys Basin, therefore confirming intracommunity exchange. While some products leave little trace in the archaeological record disproportionate to their economic role [2: p.118], the exchange of clothing and other basic organic and inorganic products such as textiles and salt should be encountered within this economic system. Resuloğlu’s metal network extended to the north and the south within Anatolia; however, a direct linkage to the Syro-Mesopotamia was not detected isotopically. Even though some typological similarities such as the Ur type bead (Etd_1156) [30] have been established with Mesopotamia, these must be Anatolian-made copies of imports.

The north-central Anatolian communities have been long identified as part of the Hatti culture that dominated the Halys Basin in the 3^rd^ millennium BC. The region’s metal corpus has also been linked to local and skilled Hattian craftspeople. Hitherto, Resuloğlu as a small, self-sustaining, middle-range society demonstrates compositionally and provenancially a diverse metal collection. The consumption patterns of metals at Resuloğlu do not resonate with technologically deterministic models. The functionally or religio-political display of metals does not seem the case at the site. The existence of bronze objects in most of the burials signifies that elites were not necessary for the consumption of certain metals or valuables.

## Conclusions

This study presents analytical and archaeological information on the metal assemblage of Resuloğlu, which is spatially and temporally the largest fully provenanced and analyzed archaeological sample set from the 3^rd^ millennium BC north-central Anatolia. The comprehensive assessment of the metal collection in its archaeological context with a well-established chronology strengthens the significance of the study. The interdisciplinary approach demonstrates variability over the dimensions of metal and alloy use, typology, and context.

White and Hamilton [81] argue that cherry-picked samples employed in most of the regional ancient metallurgy research overlook important elements of metal collections, thus resulting in prejudiced conclusions regarding the role of metal in ancient societies. This statement is true for archaeometallurgy research in Anatolia, whereby bronze, elites, and long-distance trade have been disproportionately discussed for decades over the possible variable place of metals in everyday society. Trade and control over surplus are important for the emergence of social inequality but not always the reason [79: p. 251].

Agriculture and, to a certain extent, textile production represent the pillars of Resuloğlu’s subsistence economy. Two decades of excavation have yielded no metal production-related archaeological context or remains. The settlers of the site appear as consumers of metal products. Considering the accumulation of metals at the Resuloğlu cemetery, the flow of such commodities and exchange/trade networks should be examined to comprehend the sphere of interaction and socio-economic relations of the site. We argue that there is an intracommunity exchange in the region, for which Resuloğlu provided grain and textiles and in return metal items. The compositional and isotopic analyses support such networks and economic relationships. Additionally, comparing Resuloğlu with contemporaneous sites supports the fact that the metal consumption in all of the 3^rd^ millennium BC communities is not equal.

Resuloğlu presents a high degree of variability in metal types and alloys indicative of community-driven choices and networks that were not under the control of social elites or any top-down political system. The evidence supports a picture of decentralized production and distribution of goods shaped around local preferences. Resuloğlu maintained this system–probably with some adjustments yet unknown–during its lifespan of 400 years.

Archaeological sites in different cultural and environmental settings necessitate new, bottom-up models to comprehensively uncover the role of metals in economics [2, 81, 83]. Developing appropriate fresh models for understanding the 3^rd^ millennium BC Anatolian metallurgy require not only new excavations and archaeometric analysis, i.e., data, but also a new theoretical framework. Resuloğlu as a middle-range society presents clear evidence of nonstate, decentralized consumption of functionally and technologically diverse metal products. We argue that heterarchical local and regional exchange networks operated here. This opens a new discussion in the bronze age metallurgy of Anatolia to rethink models favoring elite-controlled production and consumption of metals.

The concept of middle-range societies is not novel to world archaeology but is here proposed for the first time for the 3^rd^ millennium BC north-central Anatolian communities. We believe that Resuloğlu’s data display an excellent case to push forward the shift from top-down perspectives to community-choice-driven models. Hopefully, future studies in the region explore, test, and develop our arguments to assess the role of metals in the socioeconomics of ancient Anatolia.

## Supporting information

S1 Table pXRF analysis on the Resuloğlu metal assemblage. The inventory numbers with an asterisk (*) designate artifacts with lead isotope analysis results.
(DOCX)

S2 Table Lead isotope ratios of Resuloğlu metal objects and copper ores collected from the DVS area. Objects with an asterisk (*) also have a pXRF analysis.
(DOCX)

S1 Fig Native copper samples collected from the Bakırçay region during the Delice Valley Survey (image ©Dardeniz). (JPG)

S2 Fig Copper ore samples collected from the Bakırdere region during the Delice Valley Survey (image ©Dardeniz). (JPG)

S3 Fig Swastika-shaped beads (7908_1, 7908_3) made of gold-silver-arsenic alloy (image ©Resuloğlu excavations archive). (TIF)
