# Supplementary material for: Metal consumption of a middle-range society in the late 3rd millennium BC Anatolia: A new socioeconomic approach
Source: PLoS One. 2022 Jun 3;17(6):e0269189. doi: 10.1371/journal.pone.0269189 (PMC9165867; doi:10.1371/journal.pone.0269189)
Supplement: S1 Table — The inventory numbers with an asterisk (*) designate artifacts with lead isotope analysis results. (DOCX) [file pone.0269189.s001.docx]

| ***Museum Inventory number*** | ***Alloy type*** | ***Cu (wt%)*** | ***Sn***  ***(wt%)*** | ***As (wt%)*** | ***Sb (wt%)*** | ***Pb (wt%)*** | ***Ni***  ***(wt%)*** | ***Co***  ***(wt%)*** | ***Fe***  ***(wt%)*** | ***Zn***  ***(wt%)*** | ***Bi***  ***(wt%)*** | ***Ag***  ***(wt%)*** | ***Au (wt%)*** | ***Total (wt%)*** |
| --- | --- | --- | --- | --- | --- | --- | --- | --- | --- | --- | --- | --- | --- | --- |
| 2017 | Cu-As | 87.24 | - | 9.25 | 0.01 | 0.07 | - | - | 0.28 | - | 0.01 | 0.05 | - | 96.91 |
| 7516 | Cu-Pb | 75.92 | - | 0.51 | - | 21.32 | 0.11 | - | 0.08 | - | 0.23 | 0.07 | 0.02 | 98.26 |
| 7703 | Cu-Sn | 97.07 | 3.01 | - | - | - | 0.13 | - | 0.11 | - | - | 0.03 | 0.01 | 100.36 |
| 7405 | Cu-As | 95.0 | - | 2.71 | - | 0.25 | - | - | 1.53 | - | - | - | - | 99.49 |
| 7414 | Cu-As-Sn | 91.69 | 7.36 | 1.13 | - | - | - | - | 0.16 | - | - | - | - | 100.34 |
| 7415 | Cu-Sn | 91.67 | 7.2 | 0.86 | - | 0.1 | - | - | 0.14 | - | - | 0.01 | - | 99.98 |
| 1064_1 | Cu | 97.56 | 0.28 | - | 0.02 | 0.03 | 0.16 | - | 0.18 | - | - | 0.029 | 0.06 | 98.319 |
| 1064_2 | Cu-Sn | 96.03 | 3.97 | - | - | 0.08 | 0.14 | - | 0.23 | - | - | 0.14 | 0.03 | 100.62 |
| 8655 | Cu-As | 93.04 | - | 3.98 | - | 0.34 | - | - | 0.62 | - | - | - | - | 97.98 |
| 8656 | Cu-As | 90.38 | - | 7.52 | - | 0.11 | - | - | 1.56 | - | - | - | - | 99.57 |
| 8908 | Cu-Sn | 93.78 | 5.2 | 0.18 | - | 1.6 | - | - | 0.07 | - | - | - | - | 100.83 |
| 8657 | Cu-Sn | 96.41 | 3.92 | 0.42 | - | 0.05 | - | - | 0.14 | - | - | - | - | 100.94 |
| 8911_1 | Cu-Sn | 93.92 | 6.26 | 0.13 | - |  | - | - | 0.28 | - | - | - | - | 100.59 |
| 8911_2 | Cu-Sn | 82.27 | 18.13 | 0.14 | - |  | - | - | 0.16 | - | - | - | - | 100.7 |
| 8660_1 | Cu-Sn | 80.71 | 18.1 | 0.19 | - | 0.88 | - | - | 0.25 | - | - | - | - | 100.13 |
| 8660_2 | Cu-Sn | 82.73 | 16.46 | 0.13 | - | 0.22 | - | - | 0.2 | - | - | - | - | 99.74 |
| 8660_3 | Cu-Sn | 97.24 | 1.95 | 0.08 | - | 0.12 | - | - | 0.42 | - | - | - | - | 99.81 |
| 8614_1 | Cu-Ag-Au-Sb | 27.3 | - | - | 4.22 |  | - | - | - | - | - | 51.96 | 16.6 | 100.08 |
| 8614_2 | Cu-Ag-Au | 45.45 | - | - | 0.78 |  | - | - | - | - | - | 23.12 | 30.64 | 99.99 |
| 8625 | Au-Ag | 0.91 | - | - | - |  | - | - | 0.35 | - | - | 3.47 | 95.36 | 100.09 |
| 8638 | Cu-As-Sn | 90.8 | 5.74 | 1.44 | - | 4.36 | - | - | 0.46 | - | - | - | - | 102.8 |
| 8639 | Cu-Sn | 91.11 | 8.14 | 0.28 | - | 0.76 | - | - | 0.07 | - | - | - | - | 100.36 |
| 8616 | Cu-Sn | 95.77 | 1.18 | 0.22 | - | 1.91 | - | - | 0.11 | - | - | - | - | 99.19 |
| 8616 | Cu-Sn-Pb | 82.79 | 5.8 | 0.62 | - | 10.1 | - | - | 0.07 | - | - | - | - | 99.38 |
| 8617 | Cu-As | 97.14 | - | 2.73 | 0.13 | 0.03 | - | 0.02 | 0.08 | 0.018 | 0.01 | 0.06 | - | 100.218 |
| 7512_1 | Cu-Sn | 94.55 | 4.9 | 0.6 | - | 0.21 | - | - | 0.14 | - | - | - | - | 100.4 |
| 7512_2 | Cu-Sn | 95.49 | 5.02 | 0.16 | - | 0.07 | - | - | 0.01 | - | - | - | - | 100.75 |
| 7513 | Cu | 99.13 | - | 0.49 | 0.07 | 0.05 | - | 0.01 | 0.13 | - | - | - | - | 99.88 |
| 7513 | Cu-As-Sn | 80.66 | 18.52 | 1.18 | 0.19 | 0.15 | - | 0.01 | 0.05 | - | - | - | - | 100.76 |
| 7518 | Cu-Sn | 84.7 | 14.79 | 0.42 | 0.08 | 0.31 | - | 0.02 | 0.1 | 0.18 | 0.06 | 0.04 | - | 100.7 |
| 7519 | Cu-Sn | 94.51 | 5.1 | 0.16 | 0.03 | 0.01 | - | - | 0.1 | - | - | - | - | 99.91 |
| 7522_1 | Cu-Sn | 84.39 | 15.71 | 0.28 | - | - | - | - | 0.26 | - | - | - | - | 100.64 |
| 7522_2 | Cu-Sn | 82.52 | 16.88 | 0.7 | - | - | - | - | 0.36 | - | - | - | - | 100.46 |
| 7525 | Cu-Sn | 94.76 | 4.72 | 0.26 | - | 0.03 | - | - | 0.22 | 0.37 | - | - | - | 100.36 |
| 7527 | Cu-Sn | 95.22 | 4.68 | 0.19 | - | 0.2 | - | 0.01 | 0.07 | 0.19 | - | - | - | 100.56 |
| 7527 | Cu | 97.09 | 0.93 | 0.15 | - | 0.1 | - | 0.15 | 0.27 | 0.24 | - | - | - | 98.93 |
| 7505_1 | Cu-Sn | 83.74 | 15.86 | 0.19 | - | 0.18 | - | - | 0.19 | - | - | - | - | 100.16 |
| 7505_2 | Cu-Sn | 93.31 | 5.91 | 0.91 | - | 0.15 | - | - | 0.01 | - | - | - | - | 100.29 |
| 7505_3 | Cu-Sn | 97.49 | 2.75 | 0.16 | - | 0.09 | - | - | 0.09 | - | - | - | - | 100.58 |
| 7505_4 | Cu-Sn | 85.71 | 14.49 | 0.2 | - | 0.32 | - | - | 0.19 | - | - | - | - | 100.91 |
| 7509 | Cu-As | 96.9 | - | 2.46 | - | 0.16 | - | - | 0.91 | - | - | - | - | 100.43 |
| 7521 | Cu-Sn | 92.82 | 7.33 | 0.07 | - | 0.04 | 0.16 | - | 0.12 | - | - | 0.04 | - | 100.58 |
| 7530 | Cu-As | 79.08 | - | 21.12 | - | 0.06 | 0.15 | - | 0.05 | - | 0.02 | 0.05 | - | 100.53 |
| 7602 | Cu-Sn | 94.72 | 5.2 | 0.05 | - | 0.19 | 0.15 | - | 0.02 | - | - | 0.05 | - | 100.38 |
| 7682 | Cu-Sn | 93.08 | 6.48 | 0.28 | - | 0.01 | 0.15 | - | 0.08 | - | - | 0.04 | - | 100.12 |
| 7684 | Cu-As | 96.59 | - | 3.59 | - | 0.05 | 0.14 | - | 0.1 | - | - | 0.05 | - | 100.52 |
| 7897_1 | Cu-Ag-Au | 25.87 | - | 0.09 | - | 0.24 | 0.02 | - | 0.19 | - | 0.05 | 65.41 | 3.87 | 95.74 |
| 7897_2 | Cu-Ag-Au | 25.73 | - | 0.11 | - | 0.23 | 0.02 | - | 0.2 | - | 0.06 | 62.25 | 5.12 | 93.72 |
| 7900 | Cu-As-Sn | 90.53 | 6.25 | 2.35 | - | 0.18 | 0.15 | - | 0.72 | - | - | 0.05 | 0.02 | 100.25 |
| 7902 | Cu-Ag | 65.7 | 0.04 | - | 0.02 | 0.16 | 0.1 | - | 0.55 | - | - | 25.37 | 0.65 | 92.59 |
| 7908_1 | Au-Ag-As | 0.72 | - | 1.25 | - | 0.02 | - | - | 0.09 | - | 0.1 | 1.47 | 93.23 | 96.88 |
| 7908_2 | Cu-Ag-Au | 5.24 | - | 0.86 | - | 0.11 | - | - | 0.22 | - | 0.06 | 1.58 | 88.16 | 96.23 |
| 7908_3 | Au-Ag-As | 0.66 | - | 1.46 | - | 0.02 | - | - | 0.09 | - | 0.1 | 1.4 | 93.35 | 97.08 |
| 7908_4 | Cu-Ag-Au | 8.65 | - | 0.88 | - | 0.01 | - | - | 0.14 | - | 0.06 | 1.35 | 85.7 | 96.79 |
| 7693 | Cu-As | 93.48 | - | 6.48 | 0.04 |  | - | - | 0.11 | 0.19 | 0.02 | 0.03 | - | 100.35 |
| 7697_1 | Cu-Sn | 94.18 | 5.9 | 0.45 | - | 0.02 | - | - | 0.05 | - | - | - | - | 100.6 |
| 7697_2 | Cu-Sn-Pb | 75.74 | 16.34 | 0.59 | - | 7.12 | - | - | 0.14 | - | - | - | - | 99.93 |
| 7699_1 | Cu-Ag | 54.58 | - | - | - | - | - | - | - | - | - | 34.46 | 0.88 | 89.92 |
| 7699_2 | Cu-Ag | 45.13 | - | - | - | - | - | - | - | - | - | 40.46 | 1.49 | 87.08 |
| 7701 | Cu-As-Ag | 92.76 | - | 4.17 | - | - | - | - | 0.6 | - | - | 1.9 |  | 99.43 |
| 7704_1 | Cu-Ag | 94.89 | - | - | - | 0.12 | - | - | 1.22 | - | - | 2.62 | 0.02 | 98.87 |
| 7704_2 | Cu-Ag | 91.83 | - | - | - | 0.07 | - | - | 0.1 | - | - | 4.9 | 0.02 | 96.92 |
| 7680_1 | Cu-Sn | 81.32 | 18.53 | 0.14 | - | 0.08 | - | - | 0.2 | - | - | - | - | 100.27 |
| 7680_2 | Cu-Sn | 92.15 | 8.51 | 0.08 | - | 0.02 | - | - | 0.22 | - | - | - | - | 100.98 |
| 7681 | Cu-As | 92.31 | - | 7.19 | 0.03 | - | 0.02 | - | 0.06 | 0.19 | 0.01 | - | - | 99.81 |
| 7681 | Cu-Sn | 89.9 | 8.53 | 0.44 | 0.09 | 0.08 | - | - | 0.07 | 0.2 | 0.01 | - | - | 99.32 |
| 7901 | Cu | 98.94 | - | 0.78 | 0.54 | - | - | - | - | - | - | - | - | 100.26 |
| 7910 | Cu-Sn | 91.49 | 7.85 | 0.2 | - | 0.45 | - | - | 0.06 | - | - | - | - | 100.05 |
| 9104 | Cu-As | 97.3 | - | 1.04 | 0.05 | 0.02 | - | 0.03 | 0.87 | 0.33 | 0.01 | - | - | 99.65 |
| 9104 | Cu | 98.21 | - | 0.26 | 0.1 | 0.02 | - | 0.03 | 1.08 | 0.34 | - | - | - | 100.04 |
| 9105 | Cu | 99.07 | - | 0.94 | - | - | - | - | 0.32 | - | - | - | - | 100.33 |
| 9105 | Cu-As | 93.5 | - | 6.85 | - | - | - | - | 0.08 | - | - | - | - | 100.43 |
| 7892 | Cu-As | 97.26 | 0.35 | 3.39 | 0.23 | 0.06 | - | 0.02 | 0.35 | - | 0.02 | - | - | 101.68 |
| 7892 | Cu-As-Sn | 91.53 | 2.11 | 6.48 | 0.4 | 0.42 | - | 0.02 | 0.09 | - | 0.02 |  |  | 101.07 |
| 9107 | Cu-As | 91.88 | 0.64 | 7.71 | 0.1 | 0.23 | 0.16 | 0.07 | 2.66 | 0.18 | - | 0.03 | - | 103.66 |
| Etd_1 | Cu-Sn | 96.18 | 2.54 | - | - | 0.43 | 0.13 | - | 0.38 | - | - | 0.04 | 0.03 | 99.73 |
| * Etd_1002 | Cu-As | 95.67 | - | 5.0 | - | - | - | - | 0.06 | - | - | 0.03 | - | 100.76 |
| Etd_1003 | Cu-Sn | 78.91 | 10.51 | 0.24 | - | - | - | - | 0.5 | - | - | - | - | 90.16 |
| Etd_1004 | Cu-Sn | 95.17 | 5.44 | 0.1 | - | 0.02 | - | - | 0.08 | - | - | - | - | 100.81 |
| Etd_1005 | Cu-As | 93.16 | - | 3.2 | - | 0.24 | - | - | 0.79 | - | - | - | - | 97.39 |
| Etd_1006 | Cu-Pb | 93.6 | - | - | 0.01 | 5.65 | - | - | 0.36 | - | - | 0.02 | - | 99.64 |
| Etd_1007 | Cu-Sn | 90.04 | 9.81 | 0.37 | - | 0.23 | - | - | 0.19 | - | - | - | - | 100.64 |
| Etd_1008 | Cu-Sn | 91.53 | 8.18 | 0.14 | - | - | 0.46 | - | 0.4 | - | - | - | - | 100.71 |
| Etd_1009 | Cu-As | 97 | - | 2.64 | - | 0.02 | 0.16 | - | 0.32 | - | - | 0.02 | - | 100.16 |
| * Etd_1011 | Cu-Ag | 4.93 | - | 0.07 | - | - | - | - | 1.63 | - | - | 27.08 | - | 33.71 |
| Etd_1013 | Cu-Sn | 97.97 | 1.78 | 0.11 | 0.01 | - | - | - | 0.21 | - | - | 0.02 | - | 100.1 |
| Etd_1015 | Cu-Sn | 95 | 4.62 | 0.7 | 0.07 | - | - | - | 0.17 | - | - | - | - | 100.56 |
| * Etd_1017 | Cu-Sn | 86.76 | 9.56 | 0.21 | - | 4.15 | - | - | 0.08 | - | - | - | - | 100.76 |
| Etd_1018 | Cu-As-Ag-Au | 2.52 | - | 1.79 | - | 0.11 | - | - | 0.24 | - | - | 3.21 | 89.61 | 97.48 |
| Etd_1020 | Cu-Sn | 88.55 | 11.04 | 0.63 | - | - | - | - | 0.14 | - | - | 0.03 |  | 100.39 |
| Etd_1021 | Cu-Sn | 96.34 | 2.93 | 0.73 | 0.01 | - | - | - | 0.31 | - | - | 0.01 | 0.05 | 100.38 |
| * Etd_1022 | Cu-Sn | 71.78 | 28.56 | - | - | - | - | - | 0.46 | - | - | - | - | 100.8 |
| Etd_1023 | Cu-Sn | 93.73 | 6.04 | 0.11 | - | - | - | - | 0.28 | - | - | 0.01 | - | 100.17 |
| * Etd_1025 | Cu-Sn | 93.65 | 6.46 | 0.08 | - | 0.02 | - | - | 0.86 | - | - | - | - | 101.07 |
| Etd_1026 | Cu-Sn | 95.66 | 4.8 | 0.07 | - | 0.01 | - | - | 0.17 | - | - | - | - | 100.71 |
| Etd_1027_1 | Cu-Sn | 92.11 | 7.93 | 0.08 | - | 0.05 | - | - | 0.42 | - | - | - | - | 100.59 |
| Etd_1027_2 | Cu-Sn | 91.63 | 9.07 | 0.09 | - | 0.01 | - | - | 0.19 | - | - | - | - | 100.99 |
| Etd_1028 | Cu-Sn | 91.13 | 1.69 | 0.07 | - | 0.02 | - | - | 0.26 | 1.95 | - | - | - | 95.12 |
| Etd_1030 | Cu-Sn | 94.83 | 4.62 | 0.08 | - | 0.01 | - | - | 0.5 | - | - | - | - | 100.04 |
| * Etd_1031 | Cu-As | 92.54 | - | 8.1 | - | 0.01 | - | - | 0.26 | - | - | 0.01 |  | 100.92 |
| Etd_1033 | Cu | 96.32 | - | - | - | 2.75 | - | - | 0.45 | - | - | - | - | 99.52 |
| Etd_1079 | Cu-Sn | 90.12 | 9.06 | 0.15 | - | 0.07 | 0.14 | - | 0.4 | - | - | 0.06 | 0.01 | 100.01 |
| Etd_1080_1 | Cu | 97.77 | 0.93 | 0.4 | - | 0.02 | - | - | 1.03 | - | - | 0.05 | 0.03 | 100.23 |
| Etd_1080_2 | Cu-Sn | 94.95 | 4.49 | 0.7 | 0.04 | 0.04 | 0.14 | - | 0.37 | - | - | 0.05 | 0.06 | 100.84 |
| Etd_1081 | Cu-Sn | 68.69 | 30.94 | 0.2 | 0.01 | 0.83 | 0.16 | - | 0.21 | - | 0.01 | 0.04 | 0.14 | 101.23 |
| Etd_1082 | Cu-As | 83.8 | - | 16.32 | 0.01 | 0.01 | 0.15 | - | 0.04 | - | 0.01 | 0.03 | - | 100.37 |
| Etd_1083 | Cu-As-Ag | 90.03 | - | 3.51 | - | 0.18 | 0.13 | - | 0.58 | - | - | 4.75 | 0.15 | 99.33 |
| Etd_1084 | Cu-As | 92.49 | - | 7.5 | - | 0.01 | 0.17 | - | 0.16 | - | - | 0.04 | - | 100.37 |
| Etd_1085 | Cu-Sn | 96.62 | 3.14 | 0.53 | - | 0.01 | 0.16 | - | 0.39 | - | - | 0.04 | - | 100.89 |
| Etd_1086 | Cu-As | 88.96 | - | 10.81 | - | - | 0.15 | - | 0.28 | - | - | 0.05 | 0.02 | 100.27 |
| Etd_1087 | Cu-Sn | 84.62 | 13.57 | 0.18 | - | 0.18 | 1.6 | - | 0.24 | - | - | 0.03 | - | 100.42 |
| Etd_1088_1 | Cu-Sn | 55.09 | 39.82 | 0.13 | 0.01 | 4.93 | 0.78 | - | 0.16 | - | 0.07 | 0.02 | - | 101.01 |
| Etd_1088_2 | Cu-As-Sb | 96.37 | - | 1.57 | 2.2 | 0.05 | 0.15 | - | 0.08 | - | - | 0.09 | 0.01 | 100.52 |
| Etd_1089 | Cu-Sn | 93.38 | 7.31 | 0.23 | - | 0.03 | 0.14 | - | 0.21 | - | - | 0.04 | 0.04 | 101.38 |
| Etd_1091 | Cu-Sn | 84.95 | 14.67 | 0.23 | - | 0.04 | 0.15 | - | 0.13 | - | - | 0.04 | 0.02 | 100.23 |
| Etd_1092 | Cu-Sn | 89.72 | 10 | 0.11 | - | 0.57 | 0.18 | - | - | - | - | 0.05 | 0.02 | 100.65 |
| Etd_1093 | Cu-Sn | 96.75 | 2.15 | - | - | 0.38 | 0.13 | - | 0.53 | - | - | 0.05 | 0.01 | 100 |
| Etd_1094 | Cu-Sn | 92.81 | 6.38 | 0.14 | - | 0.37 | 0.13 | - | 0.68 | - | - | 0.06 | 0.02 | 100.59 |
| Etd_1095 | Cu-Sn | 90.54 | 9.62 | 0.11 | - | 0.02 | 0.14 | - | 0.31 | - | - | 0.05 | 0.02 | 100.81 |
| Etd_1096_1 | Cu-Sn | 92.5 | 6.34 | - | - | 0.62 | 0.12 | - | 0.36 | - | - | 0.06 | 0.03 | 100.03 |
| Etd_1096_2 | Cu-Sn | 75.41 | 23.37 | 0.59 | 0.02 | 0.77 | 0.2 | - | 0.15 | - | 0.02 | 0.13 | 0.26 | 100.92 |
| Etd_1096_3 | Cu-Sb | 96.29 | - | 0.86 | 1.44 | 0.09 | 0.17 | - | 0.17 | - | - | 0.22 | 0.18 | 99.42 |
| Etd_1096_4 | Cu-Sn | 80.27 | 17.61 | - | - | 1.72 | 0.16 | - | 0.25 | - | - | 0.03 | 0.02 | 100.06 |
| Etd_1097 | Cu-Sn | 94.82 | 5.12 | 0.14 | - | 0.02 | 0.16 | - | 0.14 | - | - | 0.04 | 0.02 | 100.46 |
| Etd_1098 | Cu-Sn | 97.48 | 2.31 | 0.25 | 0.01 | 0.02 | 0.15 | - | 0.26 | - | - | 0.05 | 0.04 | 100.57 |
| Etd_1099 | Cu-Sn | 92.71 | 7.39 | 0.29 | - | 0.02 | 0.15 | - | 0.18 | - | - | 0.06 | 0.02 | 100.82 |
| Etd_11 | Cu-Sn | 91.07 | 9.13 | 0.18 | - | 0.02 | 0.16 | - | 0.08 | - | - | 0.03 | - | 100.67 |
| * Etd_1100 | Cu-Sn | 95.49 | 2.91 | 0.08 | - | 0.99 | 0.13 | - | 0.45 | - | 0.01 | 0.08 | 0.02 | 100.16 |
| Etd_1101 | Cu-Sn | 93.78 | 5.79 | - | - | 0.13 | 0.14 | - | 0.31 | - | - | 0.04 | 0.01 | 100.2 |
| * Etd_1102 | Cu | 98.38 | 0.32 | - | - | 0.08 | 0.13 | - | 0.27 | - | - | 0.11 | 0.01 | 99.3 |
| Etd_1103 | Cu-Sn | 99.15 | 1.14 | 0.26 | - | 0.04 | 0.15 | - | 0.13 | - | - | 0.03 | 0.03 | 100.93 |
| Etd_1105 | Cu-Sn | 94.91 | 4.84 | 0.2 | - | 0.23 | 0.15 | - | 0.45 | - | - | 0.04 | 0.04 | 100.86 |
| Etd_1106 | Cu-Sn | 95.03 | 4.91 | 0.01 | - | 0.04 | 0.13 | - | 0.27 | - | - | 0.01 | 0.01 | 100.41 |
| Etd_1107 | Cu-Sn | 95.85 | 4.03 | - | - | - | 0.18 | - | 0.34 | - | - | 0.05 | 0.02 | 100.47 |
| Etd_1108 | Cu-As-Sn | 90.84 | 5.02 | 1.92 | 0.03 | 2.45 | 0.15 | - | 0.06 | - | 0.02 | 0.05 | 0.01 | 100.55 |
| Etd_1110 | Cu-Sn | 97.48 | 2.02 | 0.33 | 0.02 | 0.03 | 0.16 | - | 0.16 | - | - | 0.05 | 0.01 | 100.26 |
| Etd_1111 | Cu-As | 97.23 | - | 1.24 | - | 0.15 | 0.18 | - | 0.79 | - | - | 0.05 | 0.02 | 99.66 |
| Etd_1112 | Cu-Sn | 91.32 | 8.65 | - | - | 0.03 | 0.15 | - | 0.42 | - | - | 0.04 | 0.02 | 100.63 |
| Etd_1113 | Cu-Sn | 97.06 | 2.43 | - | - | 0.06 | 0.21 | - | 0.65 | - | - | 0.09 | 0.06 | 100.56 |
| Etd_1114 | Cu-Sn | 84.97 | 14.01 | - | - | 1.02 | 0.15 | - | 0.25 | - | - | 0.04 | 0.02 | 100.46 |
| Etd_1115 | Cu-Sn | 88.71 | 11.49 | 0.14 | - | 0.02 | 0.14 | - | 0.07 | - | - | 0.02 | 0.01 | 100.6 |
| Etd_1116 | Cu-Sn | 73.84 | 25.86 | 0.54 | - | 3.09 | 0.14 | - | 0.08 | - | 0.06 | 0.05 | 0.02 | 103.68 |
| Etd_1117 | Cu-As | 96.8 | - | 3.74 | - | 0.03 | 0.15 | - | 0.1 | - | - | 0.05 | - | 100.87 |
| Etd_1118 | Cu-As | 91.9 | - | 8.74 | - | 0.03 | 0.13 | - | 0.07 | - | - | 0.05 | 0.03 | 100.95 |
| Etd_1119 | Cu-Sn | 92.8 | 6.96 | - | - | 0.7 | 0.16 | - | 0.28 | - | - | 0.05 | 0.04 | 100.99 |
| Etd_1120 | Cu-Sn | 95.03 | 4.1 | 0.16 | 0.05 | 0.03 | 0.15 | - | 0.71 | - | - | 0.06 | 0.06 | 100.35 |
| Etd_1121 | Cu-As-Sn | 83.73 | 15.31 | 1.43 | 0.06 | 0.04 | 0.15 | - | 0.21 | - | - | 0.04 | 0.02 | 100.99 |
| Etd_1126_1 | Cu-Sn | 96.01 | 3.68 | 0.16 | - | 0.07 | 0.14 | - | 0.15 | - | - | 0.03 | - | 100.24 |
| Etd_1126_2 | Cu-Sn | 96.74 | 4.04 | 0.15 | - | 0.16 | 0.14 | - | 0.1 | - | - | 0.03 | - | 101.36 |
| Etd_1127_1 | Cu-Sn | 97.61 | 2.46 | 0.2 | - | 0.17 | 0.14 | - | 0.13 | - | - | 0.03 | - | 100.74 |
| Etd_1127_2 | Cu-Sn | 93.28 | 6.67 | 0.07 | - | 0.38 | 0.16 | - | 0.11 | - | - | 0.05 | - | 100.72 |
| Etd_1128_1 | Cu | 99.29 | 0.37 | 0.07 | - | 0.19 | 0.16 | - | 0.2 | - | - | 0.04 | - | 100.32 |
| Etd_1128_1 | Cu-Sn | 98.33 | 2.07 | 0.12 | - | 0.03 | 0.14 | - | 0.09 | - | - | 0.04 | - | 100.82 |
| Etd_1128_2 | Cu-Sn | 94.87 | 5.05 | 0.12 | - | 0.06 | 0.14 | - | 0.07 | - | - | 0.04 | - | 100.35 |
| Etd_1128_3 | Cu-Sn | 93.87 | 5.6 | 0.35 | - | 0.1 | 0.13 | - | 0.06 | - | - | 0.05 | - | 100.16 |
| Etd_1129 | Cu-As | 76.51 | - | 23.54 | - | 0.01 | 0.25 | - | 0.07 | - | - | 0.01 | 0.01 | 100.4 |
| Etd_1130 | Cu-As | 94.76 | - | 4.74 | - | 0.01 | 0.14 | - | 0.36 | - | - | 0.05 | - | 100.06 |
| Etd_1131 | Cu-Sn | 94.82 | 4.65 | 0.33 | - | 0.03 | 0.15 | - | 0.33 | - | - | 0.05 | - | 100.36 |
| Etd_1132 | Cu-Sn | 72.62 | 24.71 | 0.6 | - | 1.77 | 0.14 | - | 0.84 | - | - | 0.06 | - | 100.74 |
| Etd_1133 | Cu-Sn | 96.1 | 4.15 | 0.29 | - | 0.05 | 0.23 | - | 0.11 | - | - | 0.05 | - | 100.98 |
| Etd_1134 | Cu-As | 97.61 | - | 1.69 | - | 0.02 | 0.14 | - | 1.32 | - | - | 0.04 | - | 100.82 |
| Etd_1135 | Cu-Sn | 84.78 | 14.64 | 0.56 | - | 0.12 | 0.15 | - | 0.34 | - | - | 0.05 | - | 100.64 |
| Etd_1136 | Cu-Sn | 98.94 | 1.69 | - | - | 0.24 | 0.16 | - | 0.1 | - | - | 0.05 | - | 101.18 |
| Etd_1137 | Cu-Sn | 80.92 | 18.93 | 0.56 | - | 0.06 | 0.13 | - | 0.14 | - | - | 0.04 | 0.01 | 100.79 |
| Etd_1138 | Cu-Sn | 97.15 | 2.85 | 0.1 | - | 0.01 | 0.13 | - | 0.15 | - | - | 0.05 | - | 100.44 |
| Etd_1139 | Cu-Sn | 93.95 | 5.32 | - | - | 0.86 | 0.15 | - | 0.1 | - | - | 0.04 | - | 100.42 |
| Etd_1140 | Cu-Sn | 90.92 | 9.39 | 0.17 | 0.03 | 0.04 | 0.15 | - | 0.18 | - | - | 0.05 | - | 100.93 |
| Etd_1141 | Cu-As | 91.44 | - | 8.72 | - | 0.02 | 0.15 | - | 0.1 | - | - | 0.05 | - | 100.48 |
| Etd_1142 | Cu-Sn | 98.92 | 2.09 | - | - | 0.02 | 0.17 | - | 0.2 | - | - | 0.05 | - | 101.45 |
| * Etd_1143 | Cu-Sn | 85.54 | 16.35 | 0.12 | - | 0.12 | 0.14 | - | 0.3 | - | - | 0.04 | - | 102.61 |
| Etd_1144 | Cu-Sn | 95.83 | 4.07 | 0.14 | - | 0.09 | 0.15 | - | 0.35 | - | - | 0.05 | - | 100.68 |
| Etd_1145 | Cu-Sn | 78.82 | 9.73 | 0.19 | - | 11.27 | 0.13 | - | 0.29 | - | 0.1 | 0.1 | 0.02 | 100.65 |
| Etd_1146 | Cu-As | 82.74 | 0.72 | 16.78 | - | 0.02 | 0.15 | - | 0.12 | - | - | 0.03 | - | 100.56 |
| Etd_1147 | Cu-As | 95.05 | - | 5.56 | - | 0.01 | 0.18 | - | 0.07 | - | - | 0.04 | - | 100.91 |
| Etd_1148 | Cu-Sn | 98.24 | 1.3 | 0.07 | - | 0.03 | 0.14 | - | 0.4 | - | - | 0.05 | - | 100.23 |
| Etd_1149 | Cu-Sn | 88.0 | 11.45 | 0.19 | - | 0.05 | 0.14 | - | 0.24 | - | - | 0.05 | 0.01 | 100.13 |
| Etd_1150 | Cu | 98.51 | 0.63 | 0.11 | - | 0.05 | 0.17 | - | 0.44 | - | - | 0.04 | - | 99.95 |
| Etd_1151_1 | Ag | 0.79 | - | - | - | 0.02 | - | - | 0.37 | - | - | 41.51 | 0.04 | 42.73 |
| Etd_1151_2 | Cu-Sn | 88.59 | 11.02 | 0.14 | - | 0.12 | 0.14 | - | 0.26 | - | - | 0.04 | - | 100.31 |
| Etd_1152 | Cu-As | 95.35 | - | 2.03 | - | 0.03 | 0.32 | - | 2.09 | - | - | 0.04 | - | 99.86 |
| Etd_1153_1 | Cu-Sn | 89.68 | 10.28 | 0.16 | - | 0.12 | 0.15 | - | 0.17 | - | - | 0.04 | - | 100.6 |
| Etd_1153_2 | Cu-Sn | 89.41 | 9.78 | 0.49 | 0.02 | 0.18 | 0.16 | - | 0.09 | - | - | 0.05 | - | 100.18 |
| Etd_1153_3 | Cu-Sn | 93.36 | 7.79 | 0.16 | - | 0.08 | 0.18 | - | 0.27 | - | - | 0.07 | - | 101.91 |
| Etd_1154 | Cu-Sn | 85.28 | 14.47 | 0.24 | - | 0.03 | 0.15 | - | 0.25 | - | - | 0.06 | - | 100.48 |
| Etd_1155 | Cu-As-Ag-Au | 1.91 | - | 1.91 | - | 0.02 | - | - | 0.04 | - | - | 2.41 | 91.21 | 97.5 |
| Etd_1155 | Cu-As-Au | 89.86 | - | 2.5 | - | - | 0.13 | - | - | - | 0.01 | 0.55 | 6.52 | 99.57 |
| Etd_1156 | Cu-Ag-Au | 10.94 | - | 0.15 | - | 0.05 | 0.04 | - | 2.38 | - | - | 37.71 | 4.82 | 56.09 |
| Etd_1172 | Cu-As | 97.03 | - | 1.96 | - | 0.02 | 0.15 | - | 1.35 | - | - | 0.05 | - | 100.56 |
| Etd_1173 | Cu-Sn | 82.46 | 16.6 | 0.71 | - | 0.07 | 0.14 | - | 0.6 | - | - | 0.06 | 0.01 | 100.65 |
| Etd_1194 | Au-As | 0.35 | - | 2.09 | - | 0.02 | - | - | 0.04 | - | 0.11 | 0.93 | 94.23 | 97.77 |
| * Etd_1195 | Cu-As-Ag-Au | 1.89 | - | 2.0 | - | 0.1 | - | - | 0.13 | - | 0.09 | 2.94 | 90.52 | 97.67 |
| Etd_1196 | Cu-As-Sn | 93.22 | 3.66 | 3.11 | 0.05 | 0.19 | 0.15 | - | 0.06 | - | - | 0.05 | - | 100.49 |
| Etd_1198 | Cu-Sn | 91.59 | 7.83 | 0.17 | 0.15 | 0.02 | 0.12 | - | 0.38 | - | - | 0.05 | - | 100.31 |
| Etd_1199 | Cu-As | 80.22 | - | 20.45 | - | - | - | - | 0.12 | - | - | 0.03 | 0.01 | 100.83 |
| Etd_12 | Cu | 97.7 | - | 0.68 | - | 0.03 | 0.16 | - | 1.23 | - | - | 0.06 | 0.02 | 99.88 |
| Etd_1200 | Cu-Sn | 87.89 | 11.35 | 0.1 | - | 0.02 | 0.16 | - | 0.76 | - | - | 0.05 | - | 100.33 |
| Etd_1201 | Cu-Sn | 94.83 | 4.64 | 0.14 | - | 0.02 | 0.16 | - | 0.3 | - | - | 0.04 | - | 100.13 |
| Etd_1202 | Cu-Sn | 95.28 | 4.27 | 0.22 | 0.02 | 0.03 | 0.16 | - | 0.15 | - | - | 0.05 | 0.01 | 100.19 |
| Etd_1203 | Cu-Sn | 97.87 | 12.31 | 0.19 | - | 0.06 | 0.12 | - | 0.39 | - | - | 0.06 | - | 111.0 |
| Etd_1204 | Cu | 34.04 | 0.66 | - | 0.03 | 0.25 | 0.1 | - | 2.1 | - | - | 0.48 | 0.02 | 37.68 |
| Etd_1205 | Cu-Sn | 85.76 | 14.41 | 0.15 | - | 0.31 | 0.12 | - | 0.15 | - | - | 0.05 | - | 100.95 |
| Etd_1206_1 | Cu-Sn | 93.68 | 5.8 | 0.09 | - | 0.35 | 0.15 | - | 0.05 | - | - | 0.05 | - | 100.17 |
| * Etd_1206_2 | Cu-Sn-Pb | 58.05 | 26.27 | 0.26 | - | 15.67 | 0.1 | - | 0.19 | - | 0.16 | 0.08 | 0.01 | 100.79 |
| Etd_1207 | Cu-Sn | 99.1 | 1.31 | - | - | 0.11 | 0.13 | - | 0.16 | - | - | 0.05 | - | 100.86 |
| Etd_1208 | Cu-As | 96.08 | - | 3.78 | - | - | 0.15 | - | 0.14 | - | - | 0.04 | - | 100.19 |
| Etd_1209 | Cu-As | 95.04 | - | 4.78 | - | - | 0.16 | - | 0.12 | - | 0.02 | 0.02 | - | 100.14 |
| Etd_1211 | Cu-As | 91.71 | - | 8.45 | - | 0.02 | 0.15 | - | 0.06 | - | - | 0.06 | - | 100.45 |
| Etd_1212 | Cu-As-Sn | 76.82 | 21.16 | 1.24 | - | 0.12 | 0.22 | - | 0.47 | - | - | 0.04 | - | 100.07 |
| Etd_1213 | Cu-As-Sn | 95.63 | 3.21 | 1.27 | 0.02 | 0.2 | 0.15 | - | 0.22 | - | - | 0.04 | - | 100.74 |
| Etd_1214 | Cu-As | 97.12 | - | 1.41 | - | 0.15 | 0.14 | - | 1.53 | - | - | 0.05 | - | 100.4 |
| Etd_1215 | Cu-As | 94.5 | 0.74 | 1.86 | - | 0.23 | 0.24 | - | 3.18 | - | - | 0.06 | - | 100.81 |
| Etd_1216 | Cu-As-Sn | 96.43 | 1.53 | 1.64 | - | 0.2 | 0.15 | - | 0.3 | - | - | 0.05 | - | 100.3 |
| Etd_1218 | Cu-Sn | 93.55 | 5.65 | 0.41 | 0.04 | 0.27 | 0.15 | - | 0.24 | - | - | 0.05 | - | 100.36 |
| Etd_1219 | Cu-Sn | 83.65 | 15.36 | 0.1 | - | 1.12 | 0.15 | - | 0.28 | - | - | 0.06 | - | 100.72 |
| Etd_1235 | Cu-As-Sn | 91.22 | 3.94 | 3.53 | - | 0.03 | 0.14 | - | 0.91 | - | - | 0.05 | - | 99.82 |
| Etd_1236 | Cu-Sn | 90.4 | 10.38 | 0.1 | - | 0.02 | 0.14 | - | 0.46 | - | - | 0.05 | - | 101.55 |
| * Etd_1237 | Cu-Sn | 81.66 | 17.81 | 0.14 | - | 0.03 | 0.15 | - | 0.58 | - | - | 0.05 | - | 100.42 |
| * Etd_1238 | Cu-Sn | 92.55 | 7.35 | 0.1 | - | 0.4 | 0.16 | - | 0.12 | - | - | 0.05 | - | 100.73 |
| Etd_13 | Cu-Sn | 97.8 | 2.63 | - | - | 0.03 | 0.14 | - | 0.1 | - | - | 0.05 | 0.01 | 100.76 |
| Etd_14 | Cu-Sn | 93.78 | 6.24 | 0.26 | - | 0.03 | 0.13 | - | 0.24 | - | - | 0.05 | 0.05 | 100.78 |
| Etd_15 | Cu-Sn | 84.52 | 2.04 | 0.17 | - | 0.37 | 0.14 | - | 0.6 | - | - | 0.05 | 0.14 | 88.03 |
| * Etd_16 | Cu-Sn | 85.72 | 14.09 | - | - | 0.13 | 0.14 | - | 0.19 | - | - | 0.05 | 0.01 | 100.33 |
| Etd_17 | Cu | 97.92 | 0.91 | - | - | 0.02 | 0.16 | - | 0.46 | - | - | 0.06 | 0.02 | 99.55 |
| Etd_18 | Cu-As-Sn | 84.35 | 9.18 | 4.03 | 0.02 | 2.16 | 0.15 | - | 0.2 | - | 0.01 | 0.03 | 0.01 | 100.14 |
| Etd_2 | Cu-Sn | 97.46 | 2.53 | 0.09 | - | 0.04 | 0.16 | - | 0.38 | - | - | 0.05 | 0.03 | 100.74 |
| Etd_20 | Cu-Sn | 91.63 | 7.33 | - | - | 0.24 | 0.14 | - | 0.5 | - | - | 0.06 | 0.1 | 100.0 |
| * Etd_2003_1 | Cu-As | 97.69 | - | 2.67 | - | - | - | - | 0.16 | - | - | - | - | 100.52 |
| * Etd_2003_11 | Cu-Sn | 90.94 | 9.39 | 0.57 | - | - | - | - | 0.24 | - | - | - | - | 101.14 |
| Etd_2003_12 | Cu-Sn | 97.85 | 1.78 | 0.11 | - | - | - | - |  | - | - | - | - | 99.74 |
| Etd_2003_14 | Cu-Sn-Pb | 90.09 | 4.19 | 0.17 | - | 5.3 | - | - | 0.7 | - | - | - | - | 100.45 |
| Etd_2003_2 | Cu-Sn | 95.36 | 4.3 | 0.14 | - | - | - | - | 0.25 | - | - | - | - | 100.05 |
| * Etd_2003_4 | Cu-As | 95.94 | - | 3.88 | - | - | - | - | 0.67 | - | - | - | - | 100.49 |
| Etd_2003_5 | Cu-Sn | 93.89 | 6.26 | 0.12 | - | - | - | - | 0.17 | - | - | - | - | 100.44 |
| Etd_2003_6 | Cu-Sn | 96.04 | 3.7 | 0.09 | - | - | - | - | 0.33 | - | - | - | - | 100.16 |
| Etd_2003_7 | Cu-Sn | 92.5 | 7.58 | 0.38 | - | - | - | - | 0.1 | - | - | - | - | 100.56 |
| * Etd_2003_8 | Cu-Sn | 97.13 | 1.59 | 0.11 | - | - | - | - | 0.67 | - | - | - | - | 99.5 |
| Etd_21 | Cu-Sn | 92.16 | 6.69 | - | - | 0.8 | 0.15 | - | 0.13 | - | - | 0.05 | 0.04 | 100.02 |
| Etd_23 | Cu-Sn | 90.08 | 10.24 | - | - | 0.08 | 0.18 | - | 0.21 | - | - | 0.05 | 0.04 | 100.88 |
| Etd_24 | Cu-Sn | 84.54 | 20.58 | - | 0.02 | 0.55 | 0.15 | - | 0.19 | - | - | 0.04 | 0.03 | 106.1 |
| Etd_25 | Cu-Sn-Sb | 87.69 | 9.45 | - | - | 2.8 | 0.15 | - | 0.12 | - | - | 0.04 | 0.01 | 100.26 |
| Etd_26 | Cu-As | 93.6 | - | 2.36 | - | 0.38 | 0.13 | - | 0.28 | - | 0.01 | 0.06 | 0.07 | 96.89 |
| Etd_27 | Cu-As-Ag-Au | 20.14 | - | 1.66 | - | 0.04 | - | - | 0.09 | - | 0.07 | 1.84 | 74.08 | 97.92 |
| Etd_28 | Cu-Sn | 93 | 6.04 | - | - | 0.46 | 0.12 | - | 0.29 | - | - | 0.04 | 0.07 | 100.02 |
| Etd_29 | Cu-Sn | 98.07 | 1.69 | - | - | - | 0.16 | - | 0.31 | - | - | - | - | 100.23 |
| Etd_3 | Cu-Sn | 94.42 | 5.99 | 0.09 | - | 0.02 | 0.16 | - | 0.13 | - | - | 0.05 | 0.01 | 100.87 |
| Etd_31 | Cu-Sn | 93.08 | 6.57 | 0.4 | - | 0.47 | 0.16 | - | 0.08 | - | - | 0.04 | 0.05 | 100.85 |
| * Etd_32 | Cu-As | 93.08 | 0.4 | 6.57 | - | 0.48 | 0.16 | - | 0.08 | - | - | 0.04 | 0.05 | 100.86 |
| Etd_35 | Cu-Sn | 82.14 | 17.97 | 0.16 | - | 0.04 | 0.14 | - | 0.11 | - | - | 0.06 | 0.04 | 100.66 |
| * Etd_37 | Cu-Sn | 90.03 | 9.18 | - | - | 0.96 | 0.14 | - | 0.25 | - | - | 0.03 | 0.03 | 100.62 |
| * Etd_4 | Cu-Sn | 97.52 | 2.47 | - | - | 0.02 | 0.15 | - | 0.45 | - | - | 0.05 | 0.03 | 100.69 |
| Etd_41 | Cu-As | 86.62 | - | 9.38 | - | 0.02 | 0.13 | - | 0.3 | - | - | 0.06 | 0.05 | 96.56 |
| Etd_5 | Cu | 98.09 | 0.86 | 0.33 | - | 0.02 | 0.16 | - | 0.37 | - | - | 0.05 | 0.02 | 99.9 |
| Etd_6 | Cu-As-Sn | 88.78 | 10 | 1.58 | - | 0.1 | 0.15 | - | 0.1 | - | - | 0.04 | 0.02 | 100.77 |
| * Etd_1041 | Cu | 97.76 | 0.86 | 0.2 | - | 0.03 | 0.14 | - | 0.44 | - | - | 0.05 | 0.03 | 99.51 |
| Etd_1043 | Cu-As | 96.79 | - | 3.31 | - | 0.02 | 0.17 | - | 0.14 | - | - | 0.04 | - | 100.47 |
| Etd_980 | Cu-Ag | 61.68 | - | 0.2 | 0.01 | 0.2 | 0.14 | - | 0.53 | - | 0.01 | 21.61 | 0.17 | 84.55 |
| Etd_981 | Cu-Sn | 94.55 | 5.99 | 0.33 | - | - | - | - | 0.12 | - | - | - | - | 100.99 |
| * Etd_982 | Cu-As | 92.08 | - | 7.68 | - | - | - | - | 0.11 | - | - | 0.03 | 0.03 | 99.93 |
| Etd_983 | Cu-Sn | 90.11 | 9.53 | 0.44 | - | 0.15 | - | - | 0.08 | - | - | - | - | 100.31 |
| Etd_984 | Cu-As | 98.92 | 0.12 | 1.18 | - | - | 0.16 | - | 0.41 | - | - | - | - | 100.79 |
| Etd_985 | Cu-Sn | 82.06 | 14.11 | 0.46 | - | 3.84 | - | - | 0.09 | - | - | - | - | 100.56 |
| Etd_986 | Cu-As | 50.74 | - | 53.04 | - |  | 0.11 | 0.01 | 0.05 | - | - | - | 0.04 | 103.99 |
| Etd_988 | Cu-Sn | 97.76 | 2.03 | 0.61 | 0.02 | - | - | - | 0.09 | - | - | - | - | 100.51 |
| Etd_989 | Cu-Sn | 95.74 | 4.9 | 0.14 | - | - | - | - | 0.18 | - | - | - | - | 100.96 |
| Etd_990 | Cu-Au | 3.91 | - | - | - | 2.52 | - | - | 0.08 | - | - | - | 85.92 | 92.43 |
| Etd_991 | Cu-As-Sn | 71.7 | 24.88 | 2.29 | 0.02 | - | - | - | 0.1 | - | - | - | - | 98.99 |
| * Etd_992 | Cu-Sn | 89.47 | 10.8 | 0.26 | - | 0.02 | - | - | 0.23 | - | - | - | - | 100.78 |
| Etd_993 | Cu-Sn | 93.73 | 5.89 | 0.12 | - | 0.06 | - | - | 0.49 | - | - | - | - | 100.29 |
| Etd_994 | Cu-Sn | 79.53 | 20.26 | 0.53 | 0.02 | - | - | - | 0.12 | - | - | 0.08 | - | 100.54 |
| Etd_995 | Cu-Sn | 98.96 | 1.67 | 0.52 | - | 0.17 | - | - | 0.31 | - | - | - | - | 101.63 |
| Etd_996 | Cu-Sn | 69.93 | 30.13 | 0.45 | - | 0.03 | - | - | 0.14 | - | - | - | - | 100.68 |
| * Etd_998 | Cu-Sn | 77.66 | 22.3 | 0.34 | - | 0.03 | - | - | 0.26 | - | - | 0.01 | - | 100.6 |
| * Etd_999 | Cu-Sn | 87.41 | 10.93 | 0.08 | - |  | - | - | 0.72 | - | - | - | - | 99.14 |
| * no inventory bag/M28 | Pb | 0.08 | 0.04 | - | - | 94.7 | - | - | 0.07 | - | - | - | - | 94.89 |
| no inventory bag/Mo.04_70 | Cu | 98.81 | 0.12 | 0.14 | - | 0.16 | - | - | 0.44 | - | - | - | - | 99.67 |
| * no inventory bag/Mo.04_80 | Cu-Sn | 87.56 | 11.91 | 0.18 | - | - | - | - | 0.56 | - | - | - | - | 100.21 |
| no inventory bag/ Mo.04_82 | Cu-Ag | 1.83 | - | 0.11 | - | - | - | - | - | - | - | 51.53 | 0.03 | 53.5 |
| no inventory bag/Mo06_19_M140 | Cu-As | 96.79 | - | 3.3 | - | 0.02 | 0.17 | - | 0.14 | - | - | 0.05 | - | 100.47 |
| 9104 | Cu | 98.29 | - | 0.41 | 0.08 | 0.02 | - | 0.03 | 0.77 | - | - | - | - | 99.6 |
| 9104 | Cu-Sb | 96.27 | - | 0.24 | 2.05 | 0.15 | 0.01 | 0.03 | 0.73 | - | - | - | - | 99.48 |
| 9106 | Cu | 99.02 | 0.02 | 0.11 | 0.1 | 0.25 | 0.05 | 0.05 | 0.13 | - | - | 0.04 | - | 99.77 |
| 9106 | Cu-As | 86.57 | 0.02 | 8.36 | 0.24 | 0.25 | 0.14 | 0.1 | 4.03 | - | - | 0.05 | 0.01 | 99.77 |
| Etd_1243 | Cu-Sb | 95.5 | 0.03 | 0.9 | 1.19 | 0.16 | 0.01 | 0.03 | 1.74 | - | - | 0.05 | - | 99.61 |
| Etd_1243 | Cu-As | 93.77 | - | 5.11 | 0.5 | 0.04 | - | 0.01 | 0.24 | - | - | 0.07 | - | 99.74 |
| Etd_1244 | Cu-Sb | 98.26 | 0.25 | 0.02 | 1.06 | 0.2 | - | 0.02 | 0.27 | - | - | 0.05 | - | 100.13 |
| 9651 | Cu-As | 95.41 | 3.9 | 0.1 | 0.02 | - | 0.01 | 0.3 | - | - | - | 0.04 | - | 99.78 |
| Etd_1248 | Cu-Sb | 95.74 | 0.78 | 0.73 | 1.1 | 0.65 | - | 0.03 | 0.73 | - | - | 0.03 | 0.01 | 99.8 |
| Etd_1248 | Cu | 97.43 | 0.67 | 0.83 | 0.23 | 0.45 | 0.01 | 0.02 | 0.11 | - | - | 0.04 | - | 99.79 |
| Etd_1260 | Cu-As | 91.78 | - | 5.24 | 0.11 | 0.02 | 0.04 | 0.04 | 2.39 | - | - | 0.04 | 0.01 | 99.67 |
| Etd_1260 | Cu-As-Sb | 91.02 | 0.01 | 1.76 | 1.45 | 0.02 | - | 0.06 | 4.8 | - | - | 0.09 | 0.03 | 99.24 |
| Etd_1261 | Cu-As | 97.5 | - | 1.27 | 0.2 | 0.2 | 0.01 | 0.03 | 0.5 | - | - | 0.05 | - | 99.76 |
| Etd_1276 | Cu-Sb | 97.39 | 0.01 | 0.35 | 1.22 | 0.01 | - | 0.01 | 0.57 | - | - | 0.08 | 0.01 | 99.65 |
| Etd_1290 | Cu | 98.8 | 0.39 | 0.02 | 0.19 | 0.14 | 0.02 | 0.3 | - | - | - | 0.03 | - | 99.89 |
| Etd_1303 | Cu | 99.01 | 0.48 | 0.05 | 0.04 | 0.01 | - | 0.01 | 0.1 | - | - | 0.04 | - | 99.74 |
| Etd_1304 | Cu-As | 97.98 | - | 1.51 | 0.11 | 0.02 | - | 0.01 | 0.09 | - | - | 0.03 | - | 99.75 |
| Etd_1305_1 | Cu | 96.87 | 0.81 | 0.07 | 0.26 | 0.88 | - | 0.03 | - | - | - | 0.02 | 0.01 | 98.95 |
| Etd_1305_1 | Cu-Sb | 94.64 | 0.94 | 0.05 | 2.5 | 0.55 | - | 0.04 | - | - | - | 0.16 | 0.03 | 98.91 |
| Etd_1305_2 | Cu | 98.76 | 0.27 | 0.03 | 0.09 | 0.31 | - | 0.02 | - | - | - | 0.03 | - | 99.51 |
| Etd_1305_2 | Cu-Sn-Sb | 89.18 | 1.45 | 0.03 | 5.77 | 1.12 | - | 0.04 | - | - | - | 0.43 | 0.13 | 98.15 |
| Etd_1306_1 | Cu | 98.88 | 0.3 | - | 0.24 | 0.07 | - | 0.02 | 0.18 | - | - | 0.09 | 0.01 | 99.79 |
| Etd_1306_2 | Cu | 98.73 | 0.62 | 0.13 | 0.1 | 0.04 | - | 0.02 | 0.14 | - | - | 0.04 | - | 99.82 |
| Etd_1306_3 | Cu | 98.87 | 0.75 | 0.03 | 0.01 | 0.04 | 0.02 | 0.01 | 0.07 | - | - | 0.02 | - | 99.82 |
| Etd_1306_4 | Cu-Sn | 98.44 | 1.1 | 0.07 | 0.03 | 0.01 | - | 0.01 | 0.07 | - | - | 0.02 | - | 99.75 |
| Etd_1320 | Cu-As | 97.09 | - | 1.2 | 0.24 | 0.05 | 0.01 | 0.03 | 1.0 | - | - | 0.05 | 0.01 | 99.68 |
| Etd_1356 | Cu | 98.29 | 0.11 | - | 0.09 | 0.01 | - | 0.02 | 0.22 | - | - | 0.04 | 0.02 | 98.8 |
| Etd_1357 | Cu-As | 97.58 | - | 1.5 | 0.13 | 0.02 | - | 0.02 | 0.47 | - | - | - | - | 99.72 |
| Etd_1362 | Cu | 97.23 | 0.01 | 0.46 | 0.2 | 0.07 | - | 0.04 | 1.26 | 2.08 | - | 0.07 | 0.07 | 101.49 |

**Table 3:** pXRF analysis on the Resuloğlu metal assemblage. The inventory numbers with an asterisk (*) designate artifacts with lead isotope analysis results.
