# Supplementary material for: Metal consumption of a middle-range society in the late 3rd millennium BC Anatolia: A new socioeconomic approach
Source: PLoS One. 2022 Jun 3;17(6):e0269189. doi: 10.1371/journal.pone.0269189 (PMC9165867; doi:10.1371/journal.pone.0269189)
Supplement: S2 Table — Objects with an asterisk (*) also have a pXRF analysis. (DOCX) [file pone.0269189.s002.docx]

| ***Museum No*** | ***Lab Code*** | ***LIA results*** | | | | | | ***Standard Errors*** | | | | | |
| --- | --- | --- | --- | --- | --- | --- | --- | --- | --- | --- | --- | --- | --- |
|  |  | 206Pb/204Pb | 207Pb/204Pb | 208Pb/204Pb | 207Pb/206Pb | 208Pb/206Pb | 208Pb/207Pb | 206Pb/204Pb | 207Pb/204Pb | 208Pb/204Pb | 207Pb/206Pb | 208Pb/206Pb | 208Pb/207Pb |
| *M28 | 1 | 187411 | 15.7003 | 38.9667 | 0.8377 | 2.0792 | 2.4819 | 0.0008 | 0.0007 | 0.0019 | 0.0000 | 0.0000 | 0.0000 |
| Etd 1016 | 2 | 18.7493 | 15.8742 | 39.2527 | 0.8466 | 2.0935 | 2.4727 | 0.0173 | 0.0146 | 0.0365 | 0.0001 | 0.0001 | 0.0002 |
| Etd 14 | 3 | 18.8482 | 15.7621 | 39.1462 | 0.8362 | 2.0769 | 2.4836 | 0.0093 | 0.0079 | 0.0197 | 0.0000 | 0.0001 | 0.0001 |
| Etd 1109 | 4 | 19.1128 | 15.8446 | 39.5044 | 0.8290 | 2.0669 | 2.4933 | 0.0163 | 0.0131 | 0.0332 | 0.0001 | 0.0001 | 0.0001 |
| *Etd 1002 | 5 | 18.9538 | 15.7631 | 39.1723 | 0.8317 | 2.0680 | 2.4864 | 0.0173 | 0.0146 | 0.0348 | 0.0001 | 0.0001 | 0.0001 |
| *Etd 1112 | 6 | 18.9329 | 15.8010 | 39.2184 | 0.8347 | 2.0713 | 2.4815 | 0.0186 | 0.0156 | 0.0388 | 0.0001 | 0.0001 | 0.0002 |
| *Etd 1100 | 7 | 18.8918 | 15.6857 | 39.0332 | 0.8303 | 2.0664 | 2.4886 | 0.0026 | 0.0024 | 0.0063 | 0.0000 | 0.0001 | 0.0001 |
| *Etd 1143 | 8 | 18.7494 | 15.5443 | 38.6557 | 0.8290 | 2.0616 | 2.4868 | 0.0100 | 0.0084 | 0.0204 | 0.0000 | 0.0001 | 0.0001 |
| *Etd 1237 | 9 | 19.0436 | 16.0261 | 39.5780 | 0.8415 | 2.0783 | 2.4704 | 0.0037 | 0.0039 | 0.0076 | 0.0001 | 0.0000 | 0.0003 |
| *Etd 1195 | 10 | 18.6579 | 15.6392 | 38.6935 | 0.8382 | 2.0738 | 2.4742 | 0.0004 | 0.0003 | 0.0009 | 0.0000 | 0.0000 | 0.0000 |
| *Etd 1206_2 | 11 | 18.9064 | 15.6768 | 39.0901 | 0.8292 | 2.0675 | 2.4935 | 0.0006 | 0.0006 | 0.0017 | 0.0000 | 0.0000 | 0.0000 |
| *Etd 1238 | 12 | 18.7059 | 15.7109 | 38.8872 | 0.8399 | 2.0789 | 2.4752 | 0.0009 | 0.0011 | 0.0034 | 0.0000 | 0.0001 | 0.0001 |
| *Etd 999 | 13 | 18.9863 | 15.8199 | 39.3745 | 0.8333 | 2.0738 | 2.4889 | 0.0197 | 0.0165 | 0.0413 | 0.0001 | 0.0002 | 0.0002 |
| *M70 | 14 | 18.9080 | 15.6784 | 39.0468 | 0.8302 | 2.0677 | 2.4932 | 0.0206 | 0.0169 | 0.0419 | 0.0001 | 0.0001 | 0.0002 |
| *Etd 982 | 15 | 18.8485 | 15.6647 | 38.8897 | 0.8310 | 2.0636 | 2.4831 | 0.0100 | 0.0083 | 0.0210 | 0.0000 | 0.0001 | 0.0001 |
| *2003/11 | 16 | 19.0436 | 16.0261 | 39.5780 | 0.8415 | 2.0783 | 2.4704 | 0.0037 | 0.0039 | 0.0076 | 0.0001 | 0.0000 | 0.0003 |
| *2003/4 | 17 | 18.8194 | 15.6739 | 38.9789 | 0.8328 | 2.0713 | 2.4869 | 0.0023 | 0.0020 | 0.0052 | 0.0000 | 0.0001 | 0.0001 |
| *2003/1 | 18 | 18.7538 | 15.6850 | 38.8814 | 0.8363 | 2.0732 | 2.4789 | 0.0091 | 0.0073 | 0.0183 | 0.0000 | 0.0001 | 0.0001 |
| *Etd 992 | 19 | 19.0318 | 15.7664 | 39.2689 | 0.8284 | 2.0633 | 2.4907 | 0.0022 | 0.0019 | 0.0047 | 0.0000 | 0.0000 | 0.0000 |
| *Etd 992 | 19(2) | 19.0241 | 15.7489 | 39.2028 | 0.8278 | 2.0606 | 2.4893 | 0.0033 | 0.0028 | 0.0073 | 0.0000 | 0.0001 | 0.0001 |
| *Etd 1017 | 20 | 19.0105 | 15.7055 | 39.0780 | 0.8261 | 2.0555 | 2.4882 | 0.0016 | 0.0015 | 0.0041 | 0.0000 | 0.0001 | 0.0000 |
| *Etd 1017 | 20(2) | 19.0434 | 15.7198 | 39.0823 | 0.8254 | 2.0520 | 2.4861 | 0.0042 | 0.0035 | 0.0089 | 0.0000 | 0.0001 | 0.0001 |
| *Etd 1031 | 21 | 18.4148 | 15.6264 | 38.4534 | 0.8485 | 2.0882 | 2.4611 | 0.0059 | 0.0052 | 0.0124 | 0.0000 | 0.0001 | 0.0001 |
| *Etd 1025 | 22 | 18.9598 | 15.6961 | 39.0615 | 0.8279 | 2.0601 | 2.4886 | 0.0066 | 0.0055 | 0.0137 | 0.0000 | 0.0001 | 0.0001 |
| Etd 39 | 23 | 18.9061 | 15.6826 | 38.9049 | 0.8296 | 2.0577 | 2.4805 | 0.0036 | 0.0029 | 0.0075 | 0.0000 | 0.0000 | 0.0000 |
| *Etd 998 | 24 | 18.8947 | 15.7058 | 39.0235 | 0.8312 | 2.0652 | 2.4846 | 0.0023 | 0.0019 | 0.0050 | 0.0000 | 0.0001 | 0.0000 |
| 2005/5 | 25 | 18.9615 | 15.7082 | 39.1143 | 0.8284 | 2.0627 | 2.4901 | 0.0037 | 0.0031 | 0.0077 | 0.0000 | 0.0000 | 0.0000 |
| 2003/13, M30 | 26 | 18.9434 | 15.7472 | 39.2208 | 0.8312 | 2.0705 | 2.4908 | 0.0042 | 0.0035 | 0.0086 | 0.0000 | 0.0001 | 0.0001 |
| 2003/13, M26 | 27 | 18.9085 | 15.8053 | 39.2556 | 0.8358 | 2.0759 | 2.4838 | 0.0134 | 0.0119 | 0.0292 | 0.0001 | 0.0001 | 0.0002 |
| Etd 38 | 28 | 18.8635 | 15.6542 | 39.0385 | 0.8298 | 2.0694 | 2.4938 | 0.0006 | 0.0006 | 0.0020 | 0.0000 | 0.0001 | 0.0000 |
| *Etd 1041 | 29 | 19.0514 | 15.7640 | 39.2357 | 0.8274 | 2.0594 | 2.4891 | 0.0066 | 0.0057 | 0.0148 | 0.0000 | 0.0001 | 0.0001 |
| *2003/08. M9 | 30 | 18.7520 | 15.6385 | 38.8027 | 0.8340 | 2.0693 | 2.4813 | 0.0005 | 0.0005 | 0.0016 | 0.0000 | 0.0000 | 0.0000 |
| 2003/10. M28 | 31 | 18.6376 | 15.5830 | 38.5716 | 0.8361 | 2.0695 | 2.4752 | 0.0004 | 0.0004 | 0.0010 | 0.0000 | 0.0000 | 0.0000 |
| 2003/09. M26 | 32 | 19.0439 | 15.7603 | 39.1583 | 0.8275 | 2.0562 | 2.4849 | 0.0088 | 0.0072 | 0.0184 | 0.0000 | 0.0001 | 0.0001 |
| Etd 1014 | 33 | 18.8119 | 15.7128 | 38.9480 | 0.8353 | 2.0704 | 2.4788 | 0.0010 | 0.0010 | 0.0033 | 0.0000 | 0.0001 | 0.0000 |
| Etd 1024 | 34 | 18.7149 | 15.6182 | 38.6880 | 0.8345 | 2.0672 | 2.4771 | 0.0002 | 0.0002 | 0.0005 | 0.0000 | 0.0000 | 0.0000 |
| *Etd 16 | 35 | 18.8206 | 15.6136 | 38.7968 | 0.8296 | 2.0614 | 2.4849 | 0.0008 | 0.0007 | 0.0017 | 0.0000 | 0.0000 | 0.0000 |
| *Etd 4 | 36 | 18.8456 | 15.6490 | 38.9985 | 0.8303 | 2.0692 | 2.4920 | 0.0008 | 0.0008 | 0.0023 | 0.0000 | 0.0001 | 0.0001 |
| *Etd 37 | 37 | 18.8684 | 15.6532 | 39.0091 | 0.8296 | 2.0673 | 2.4921 | 0.0037 | 0.0031 | 0.0078 | 0.0000 | 0.0000 | 0.0000 |
| *Etd 32 | 38 | 18.7366 | 15.6751 | 38.8513 | 0.8366 | 2.0736 | 2.4785 | 0.0033 | 0.0028 | 0.0072 | 0.0000 | 0.0001 | 0.0001 |
| *Etd 1011 | 39 | 18.8609 | 15.6565 | 38.8396 | 0.8300 | 2.0592 | 2.4810 | 0.0076 | 0.0062 | 0.0155 | 0.0000 | 0.0001 | 0.0001 |
| *Etd 1022 | 40 | 19.4656 | 15.9163 | 39.6846 | 0.8175 | 2.0386 | 2.4937 | 0.0197 | 0.0163 | 0.0402 | 0.0001 | 0.0001 | 0.0002 |
| Killik Tepe yolu, ore | 42 | 18.4365 | 15.5810 | 38.1240 | 0.8451 | 2.0678 | 2.4468 | 0.0021 | 0.0018 | 0.0044 | 0.0000 | 0.0000 | 0.0000 |
| Üçoluk gallery, ore | 43 | 18.6654 | 15.5800 | 38.6367 | 0.8347 | 2.0699 | 2.4799 | 0.0004 | 0.0003 | 0.0008 | 0.0000 | 0.0000 | 0.0000 |
| Öksen deresi, ore | 44 | 18.6283 | 15.5832 | 38.6217 | 0.8365 | 2.0732 | 2.4785 | 0.0003 | 0.0003 | 0.0007 | 0.0000 | 0.0000 | 0.0000 |
| Bakırçay, ore | 45 | 18.6894 | 15.6453 | 38.8231 | 0.8371 | 2.0772 | 2.4815 | 0.0015 | 0.0014 | 0.0041 | 0.0000 | 0.0001 | 0.0001 |
| Karaevliya village entrance/  Üçoluk deresi, ore | 46 | 18.5812 | 15.6553 | 38.6543 | 0.8425 | 2.0799 | 2.4690 | 0.0048 | 0.0040 | 0.0103 | 0.0000 | 0.0001 | 0.0001 |

**Table 4:** Lead isotope ratios of Resuloğlu metal objects and copper ores collected from the Delice Valley Survey area. Objects with an asterisk (*) also have pXRF analysis.
