# Supplementary figures and images for: Metal consumption of a middle-range society in the late 3rd millennium BC Anatolia: A new socioeconomic approach
Source: PLoS One. 2022 Jun 3;17(6):e0269189. doi: 10.1371/journal.pone.0269189 (PMC9165867; doi:10.1371/journal.pone.0269189)

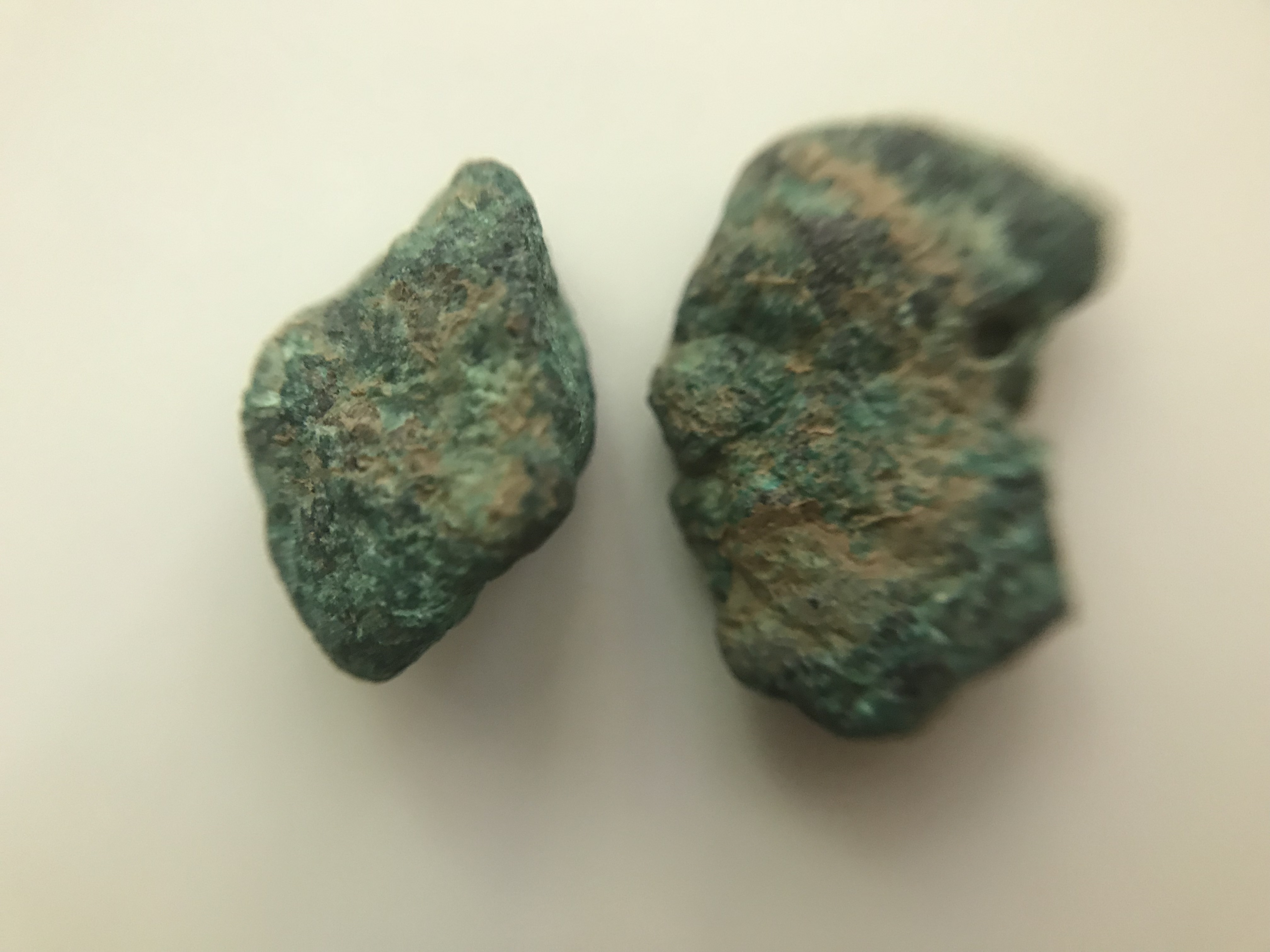

Supplement: S1 Fig — (JPG) [file pone.0269189.s003.jpg]

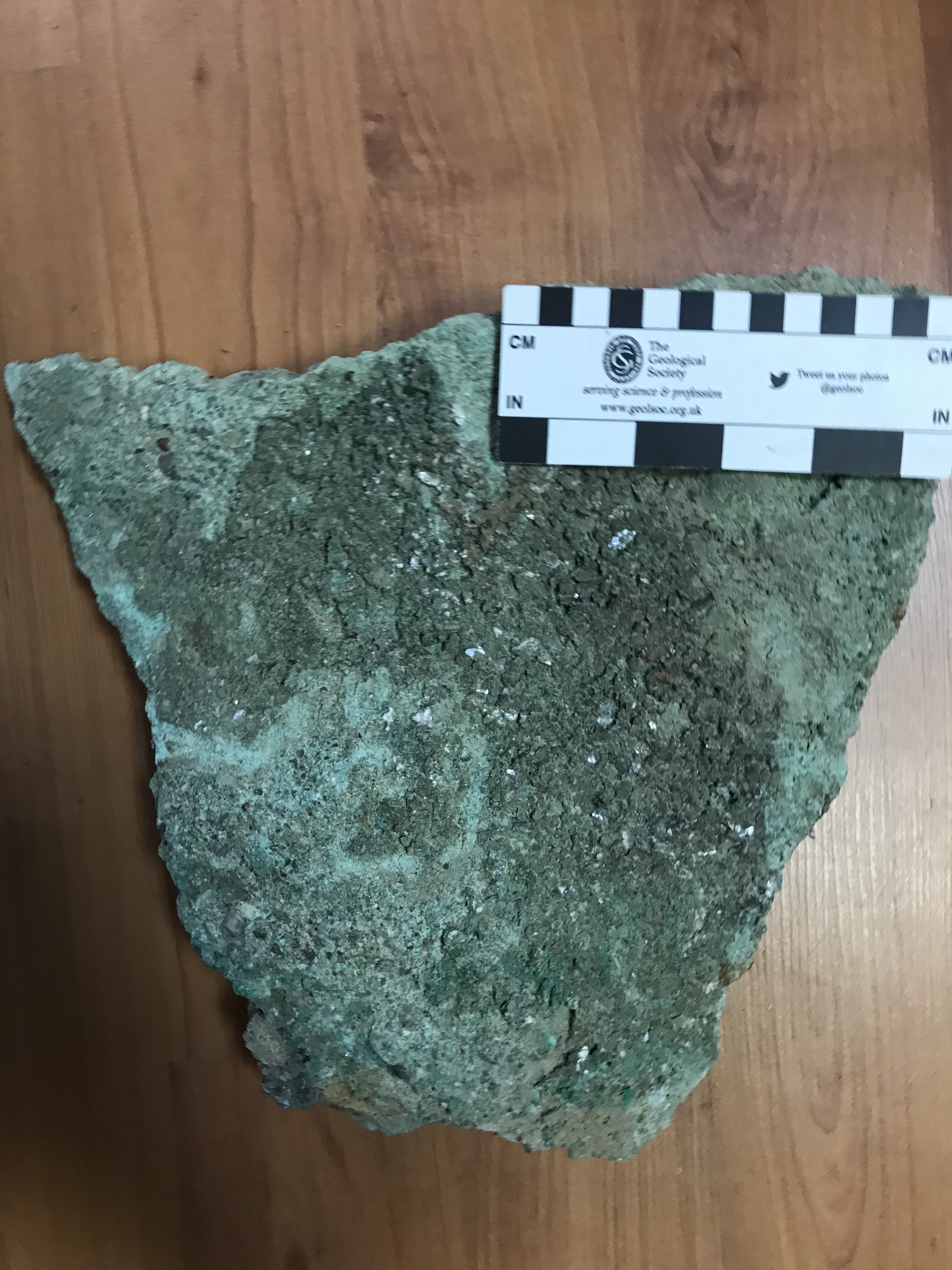

Supplement: S2 Fig — (JPG) [file pone.0269189.s004.jpg]

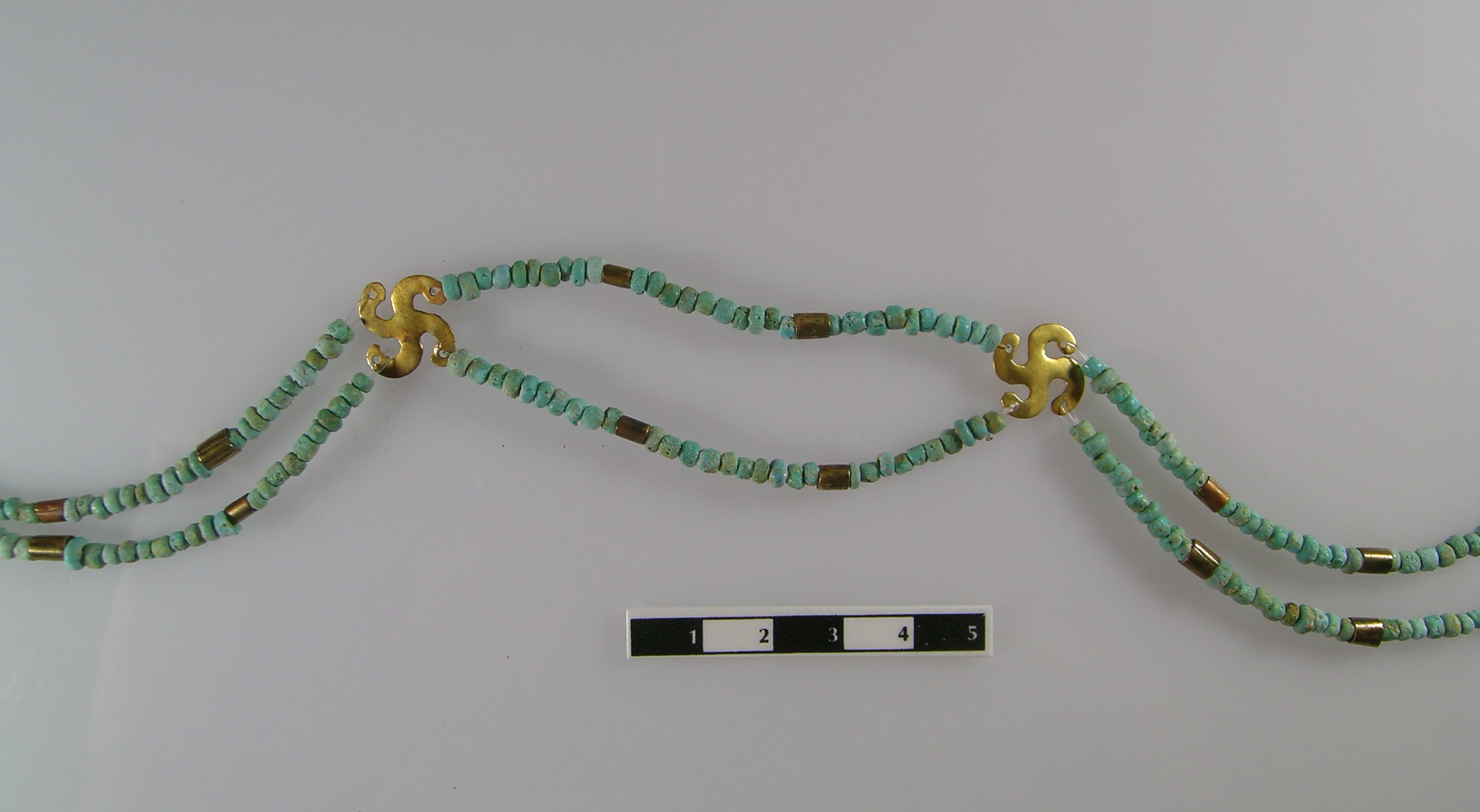

Supplement: S3 Fig — (TIF) [file pone.0269189.s005.tif]
